# Indolo[2,3-*b*]quinoxaline as a Low Reduction Potential and High Stability Anolyte Scaffold for Nonaqueous Redox Flow Batteries

**DOI:** 10.1021/jacs.3c05210

**Published:** 2023-08-16

**Authors:** Wenhao Zhang, Ryan Walser-Kuntz, Jacob S. Tracy, Tim K. Schramm, James Shee, Martin Head-Gordon, Gan Chen, Brett A. Helms, Melanie S. Sanford, F. Dean Toste

**Affiliations:** † Chemical Science Division, Lawrence Berkeley National Laboratory, 1 Cyclotron Road, Berkeley, California 94720, United States; ‡ Department of Chemistry, University of California, Berkeley, California 94720, United States; ∥ Department of Chemistry, University of Michigan, Ann Arbor, Michigan 48109, United States; § Joint Center for Energy Storage Research (JCESR), 9700 South Cass Avenue, Argonne, Illinois 60439, United States; ¶ Department of Chemistry, RWTH Aachen University, Landoltweg 1, Aachen 52074, Germany

## Abstract

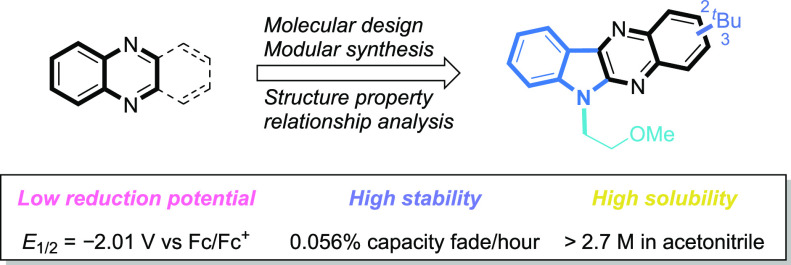

Redox flow batteries (RFBs) are a promising stationary energy storage technology for leveling power supply from intermittent renewable energy sources with demand. A central objective for the development of practical, scalable RFBs is to identify affordable and high-performance redox-active molecules as storage materials. Herein, we report the design, synthesis, and evaluation of a new organic scaffold, indolo[2,3-*b*]quinoxaline, for highly stable, low-reduction potential, and high-solubility anolytes for nonaqueous redox flow batteries (NARFBs). The mixture of 2- and 3-(*tert*-butyl)-6-(2-methoxyethyl)-6*H*-indolo[2,3-*b*]quinoxaline exhibits a low reduction potential (−2.01 V vs Fc/Fc^+^), high solubility (>2.7 M in acetonitrile), and remarkable stability (99.86% capacity retention over 49.5 h (202 cycles) of H-cell cycling). This anolyte was paired with *N*-(2-(2-methoxyethoxy)-ethyl)phenothiazine (MEEPT) to achieve a 2.3 V all-organic NARFB exhibiting 95.8% capacity retention over 75.1 h (120 cycles) of cycling.

## Introduction

The burning of fossil fuels continues to impact global warming through the generation of greenhouse gases, underscoring the necessity of adopting renewable energy sources such as wind and solar.^1,2^ However, the intermittent nature of wind and solar energy presents unique challenges to ensuring the stability of the power grid. One promising technology to help stabilize the latter is low-cost grid-scale energy storage that can help balance energy demand with energy production.^3−5^ Redox flow batteries (RFBs) are a promising solution for grid-scale energy storage and store electrical energy in the form of chemical energy within flowable anolytes and catholytes, which circulate from separate storage reservoirs through an electrochemical cell for charging and discharging.^6−9^ RFBs offer ease and economic feasibility of scale-up, as well as decoupling of energy and power.^9,10^ Aqueous-based vanadium RFBs are currently the state-of-the-art commercial systems, exhibiting excellent lifetimes and energy efficiencies.^11,12^ However, the accessible voltage of aqueous redox flow batteries is limited by the thermodynamic potential window of water (between 1.1 and 1.6 V), which constrains energy and power density.^13^ Given these limitations, along with concerns about cost, sustainability, and potential environmental issues, it is necessary to explore other types of RFBs.^14,15^ Nonaqueous redox flow batteries (NARFBs) offer a wider operating window (up to 5 V with MeCN), and modern organic synthesis allows for the tailoring of redox-active organic molecules (ROMs) to meet the stability, potential, and solubility demands of NARFBs.^16−20^ However, identifying ROMs with high stability, high oxidation (for catholyte, more positive than +1.0 V vs Fc/Fc^+^)/low reduction (for anolyte, more negative than −1.9 V vs Fc/Fc^+^) potential, and high solubility that can leverage the nonaqueous system’s wide operating window remains challenging for NARFBs.^19−21^

**Figure 1:**
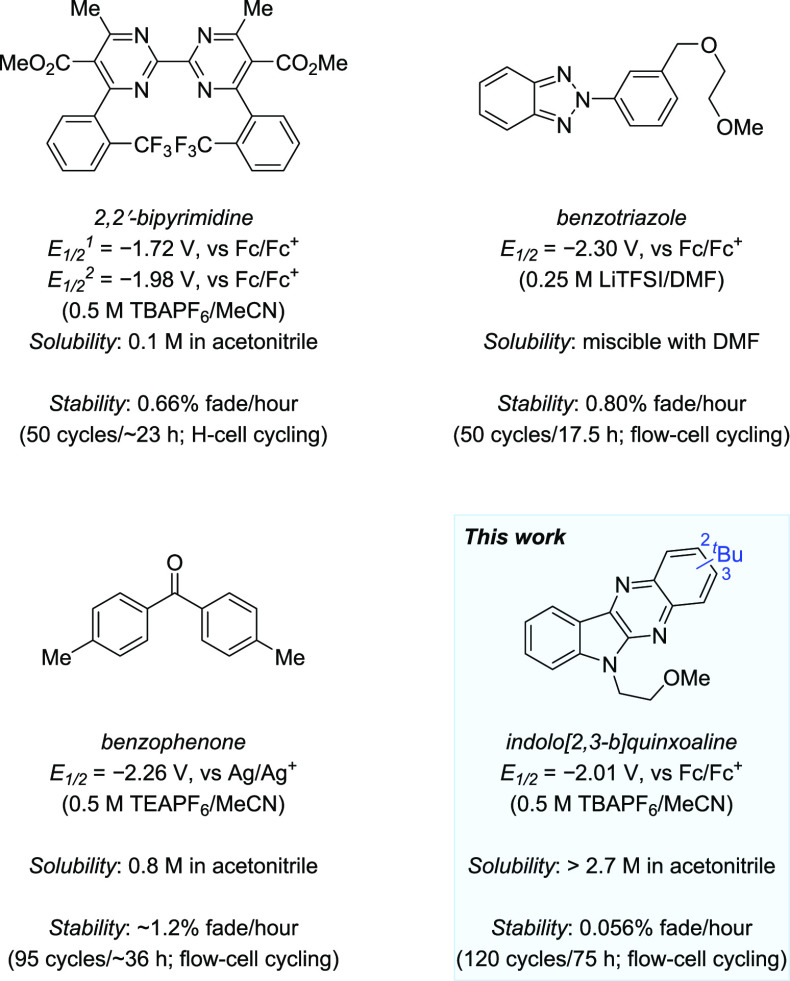
Representative organic anolyte scaffolds with reduction potentials more negative than −1.9 V (vs Fc/Fc^+^) for the NARFBs.

In recent years, there has been significant progress in the development of new ROM scaffolds for catholytes,^22−27^ anolytes,^21,28−38^ and bipolar molecules.^39−45^ These include several elegant examples of novel anolytes;^21,28−38^ however, only a few of them leverage the wide potential window of nonaqueous solvents by reaching reduction potentials more extreme than −1.9 V (vs Fc/Fc^+^) (Figure 1). The 2,2′-bipyrimidines display two-electron reductions where only the second reaches such a low reduction potential; furthermore, they exhibit moderate stability and solubility.^34^ On the other hand, benzotriazoles display an extremely low reduction potential (−2.3 V vs Fc/Fc^+^) and high solubility but only moderate electrochemical cycling stability.^35^ Benzophenone-containing scaffolds also exhibit low reduction potentials (−2.26 V vs Ag/Ag^+^) and good solubility but maintain only moderate cycling stability.^32^ Therefore, developing organic electrolytes with reduction potentials at the extreme ends of the electrochemical window of acetonitrile (<−1.9 V Fc/Fc^+^) along with high stability remains a significant unmet challenge, especially when considering the solubility requirements necessary to achieve high energy density.^19−21^ This is due to generally unfavorable trade-offs between redox potential and stability. Anolytes with lower potentials often deliver high-energy charged intermediates that are more basic and/or nucleophilic, resulting in decomposition through protonation as well as addition reactions involving electrolyte and solvent.^46^

To tackle this challenge, we hypothesized that extending the π-conjugation system of an anolyte while simultaneously incorporating π-donor *N*-atoms would allow us to both stabilize the charged intermediate while also adjusting the reduction potential to more negative values. Overall, this should enable the development of high-energy-density anolytes with improved stability and low reduction potentials. Herein, we report a new anolyte scaffold, indolo[2,3-*b*]quinoxaline, based on these design principles, that exhibits high stability and low reduction potential via delocalization via charge throughout the extended π-system while still maintaining a low reduction potential. Further molecular engineering has resulted in an anolyte with remarkably high solubility (>2.7 M in acetonitrile; >1.2 M in 0.5 M TBAPF_6_/MeCN), high stability (0.0028% fade per hour (H-cell cycling); 0.056% fade per hour (flow cell cycling)), and a low reduction potential (*E*_1/2_ = −2.01 V vs Fc/Fc^+^).

## Results and Discussion

### Design, Synthesis, and Characterization of the Indolo[2,3-*b*]quinoxaline Model Compound

The challenge we sought to overcome in achieving highly stable electrolytes for NARFBs is that the simple expansion of a conjugated π-system, a commonly employed approach for enhancing the electrochemical stability of organic anolytes, generally comes at the cost of less negative reduction potentials as well as reduced solubility due to strong π–π stacking interactions.^47^ Therefore, electron-rich atoms must be incorporated into the conjugated π-system to adjust the reduction potential, while additional functional groups must also be incorporated to improve solubility.

**Figure 2:**
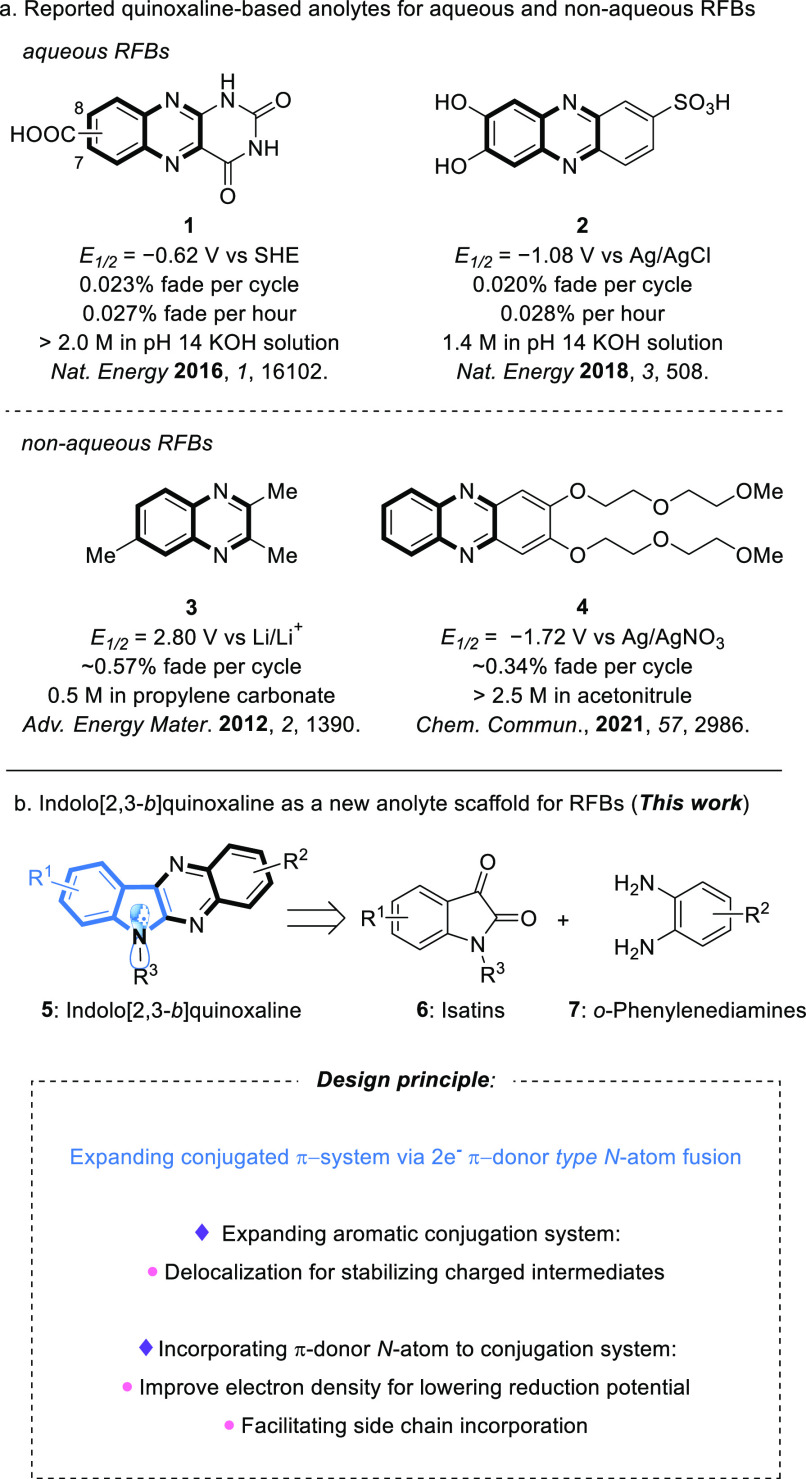
(a) Reported quinoxaline-based anolytes for aqueous and nonaqueous RFBs. (b) Design of indolo[2,3-*b*]quinoxaline as a new anolyte scaffold.

The incorporation of nitrogen atoms, which are electron-rich trivalent π-donors, offers a potential solution to the aforementioned issues. First, we expect that the p_*z*_ orbital of the nitrogen atom with the lone pair electrons on nitrogen has the correct orbital symmetry to participate in electron delocalization and to expand conjugated π-systems. Second, this donation can enhance the electron density of the entire π-system, which should result in more negative reduction potentials. Third, this atom can serve as a synthetic handle for incorporating side chains, facilitating the development of soluble derivatives. Guided by these concepts, we selected quinoxaline as the starting point to test our proposal. As shown in Figure 2a, quinoxaline^48^ and phenazine derivatives^49−52^ have been developed for aqueous RFBs. Quinoxaline-based scaffolds show excellent performance in aqueous systems but suffer from the inherent voltage limitations of aqueous solvent. A nonaqueous system (*V*_ocv_ = 2.2 V) featuring a quinoxaline derivative (**3**) overcomes the voltage limitation of aqueous systems; however, low stability is observed.^53^ On the other hand, phenazine derivative **4**, which has an expanding π-system, exhibits both moderate reduction potential (−1.72 V vs Ag/Ag^+^) and stability (∼30% capacity fade over 50 cycles; flow cycling experiment), with excellent solubility as an anolyte.^54^

We designed indolo[2,3-*b*]quinoxaline as a new motif for NARFB anolytes, inspired by these earlier works and our own concept for improving stability and lowering the reduction potential (Figure 2b). Our approach involves fusing a π-donor nitrogen atom, incorporating a seven-center eight-electron π-system to this scaffold. This fusion expands the aromatic conjugated π-system, leading to better charge delocalization in charged intermediates and, thus, enhanced stability. Furthermore, the increased electron density in the π-system was expected to result in a lowering of the reduction potential. Additionally, the *N*6-atom in the indolo[2,3-*b*]quinoxaline scaffold can serve as a handle for further derivatization. Finally, the indolo[2,3-*b*]quinoxaline motif can be synthesized using a modular and convergent route, facilitating the preparation of derivatives for evaluation.

**Figure 3:**
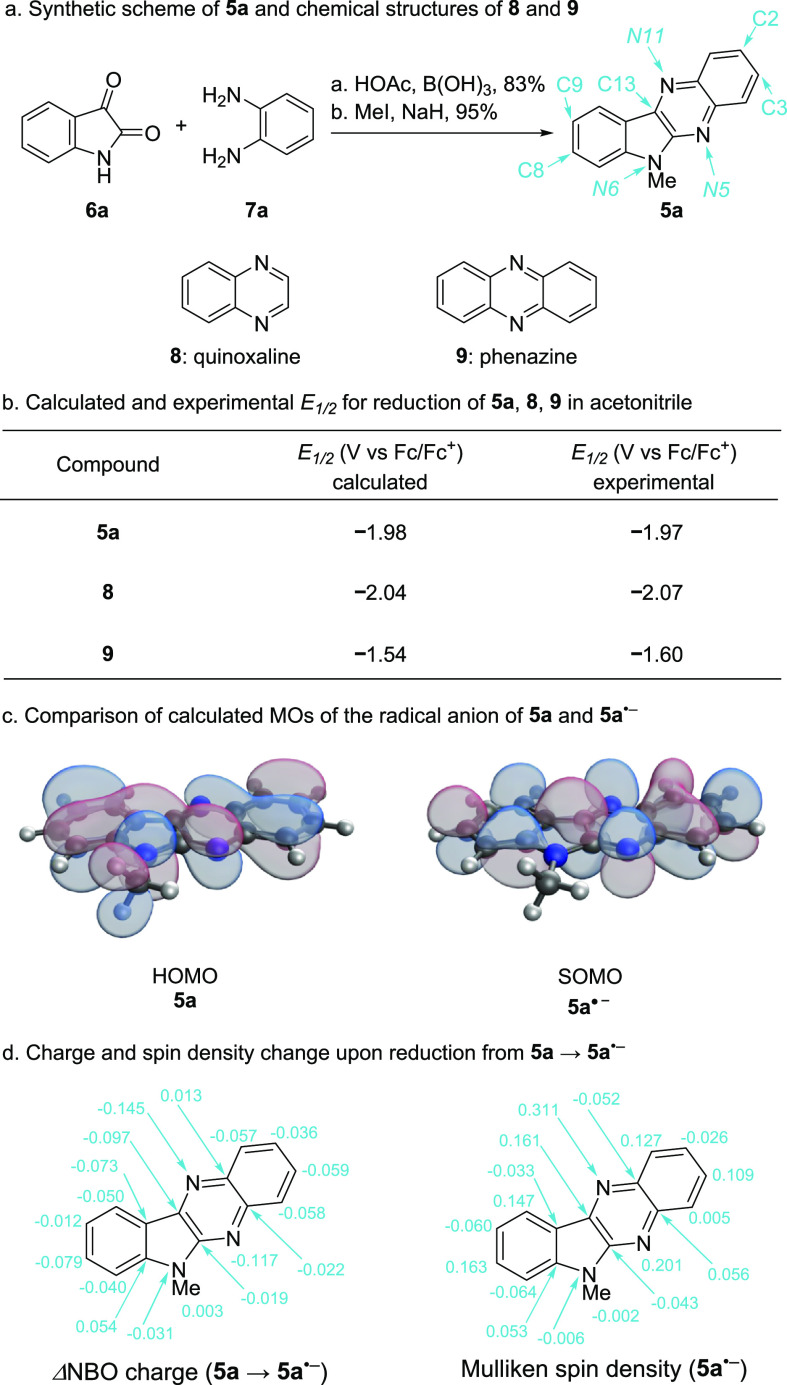
(a) Synthetic scheme of **5a** and chemical structures of **8** and **9**. (b) Calculated and experimental reduction potential of **5a**, **8**, and **9** in acetonitrile. (c) Comparison of calculated HOMO of **5a** and the SOMO of radical anion **5a^•–^**. (d) Charge and spin density changes upon reduction from **5a** to **5a^•–^**.

On the basis of this hypothesis, **5a** was synthesized via the condensation of isatin (**6a**) and *O*-phenylenediamine (**7a**) (delivering the indolo[2,3-*b*]quinoxaline core in 83% yield), followed by methylation with iodomethane to give the target anolyte in 95% yield (Figure 3a). The solubility of this unsubstituted aromatic molecule in acetonitrile is very low (33 mM compared to 58 mM for phenazine), suggesting that the expansion of the conjugated π-system resulted in decreased solubility. Therefore, further modification to the structure will be necessary to achieve high solubility.

**Figure 4:**
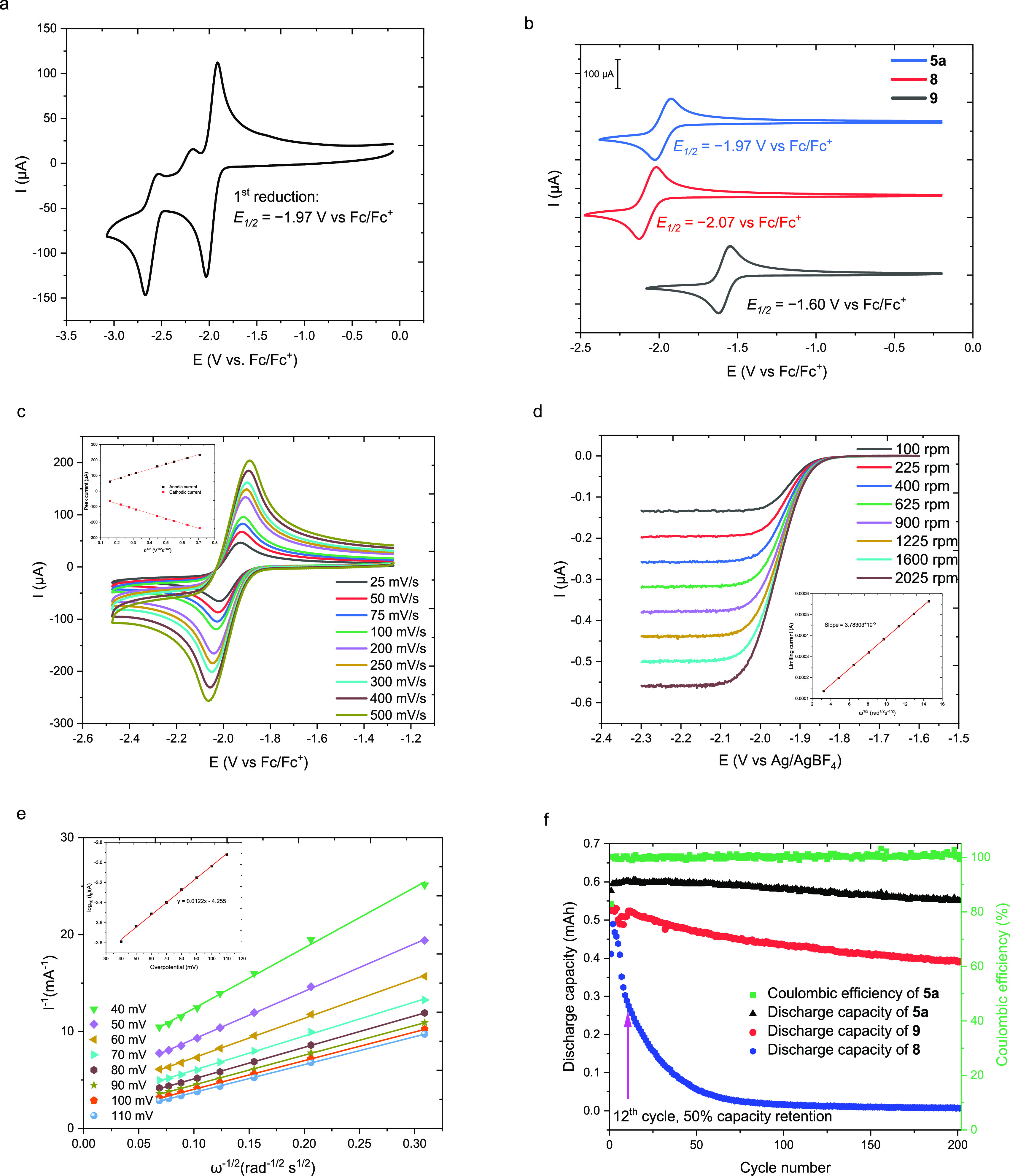
(a) CV of **5a** (5 mM) in 0.5 M TBAPF_6_/MeCN solution with a glassy carbon working electrode at a scan rate of 100 mV/s. (b) CVs of **5a** (blue, top), **8** (red, middle), and **9** (black, bottom). (c) Scan-rate-dependent CVs of **5a**. Inset: Linear relationship between the peak current density versus the square root of scan rates. (d) LSV of different rotation rates of the RDE scans of 2 mM **5a** in 0.1 M TBAPF_6_/MeCN. Inset: Linearly fitted Levich plots of **5a** in 0.1 M TBAPF_6_/MeCN. (e) Koutecky–Levich plots of **5a** in 0.1 M TBAPF_6_/MeCN. Inset: Linearly fitted Tafel plots of **5a** based on the Butler–Volmer equation as a function of overpotentials. (f) Discharge capacity versus cycle number of **5a** (black), **8** (blue), and **9** (red) and Coulombic efficiency for cycling of **5a** (green) (5 mM in 0.5 M TBAPF_6_/MeCN) in a static H-cell.

Nonetheless, with **5a** in hand, we began our evaluation of the electrochemical properties through cyclic voltammetry (CV). A 5 mM solution of **5a** in 0.5 M TBAPF_6_/MeCN was tested using the three-electrode method with a glassy carbon working electrode (0.071 cm^2^) at a scan rate of 100 mV/s. **5a** exhibits a first reversible reduction at *E*_1/2_ = −1.97 V vs Fc/Fc^+^ and a second irreversible reduction at *E*_pc_ = −2.64 V vs Fc/Fc^+^ (Figure 4a). **5a** exhibits a significantly more negative reduction potential (Δ*E*_1/2_ = 370 mV) compared with phenazine (**9**), which has its first reversible reduction at *E*_1/2_ = −1.60 V vs Fc/Fc^+^ (Figure 4b). The first reversible reduction potential of **5a** is comparable to that of quinoxaline (**8**) (*E*_1/2_ = −2.07 V vs Fc/Fc^+^; Figure 4b). These findings highlight the significant effect on reduction potential that comes from incorporating an electron-rich trivalent π-donor *N*-atom.

With these electrochemical results in hand, we selected 6-methyl-*6H*-indolo[2,3-*b*]quinoxaline (**5a**) as a model compound to examine our initial hypothesis. Density functional theory (DFT) calculations employing the TPSSh-D4 functional were validated by comparison to the measured reduction potential of **5a**/**5a**^•–^, quinoxaline (**8**/**8**^•–^), and phenazine (**9**/**9**^•–^). Using this computational protocol (detailed in the Supporting Information), the reduction potentials in acetonitrile were calculated to be −1.98, −2.04, and −1.54 V vs Fc/Fc^+^, respectively (Figure 3b), which is in excellent agreement with the experiments above. The relative reduction potentials are revealing: going from **8** to **9** expands the π-system, thereby shifting the reduction potential to a more positive value. This is due to the preferential stabilization of the reduced vs the neutral species with a more extensive π-system. From **9** to **5a**, despite making the π-system slightly larger, the addition of a π-donor trivalent nitrogen atom leads to a reduction potential that is more negative by approximately 400 mV because of an increased electron density of the aromatic core that destabilizes the reduced species. Two additional compounds were investigated computationally (see Figure SI27), further corroborating these findings on the interplay of the size and electronic properties of the π-system and their substantial effects on the reduction potential.

The highest occupied molecular orbital (HOMO) of **5a** and the singly occupied molecular orbital (SOMO) of **5a**^•–^ (the latter is nearly identical to the LUMO of **5a**; see Figure SI29) are shown in Figure 3c. The HOMO of **5a** confirms that the p_*z*_ atomic orbital of the nitrogen has the correct orbital symmetry to participate in electron delocalization, which helps expand the conjugated π-system and stabilize the charged intermediate. The change in NBO charges upon reduction of **5a** to **5a^•–^** and the condensed Mulliken spin densities of **5a^•–^** (Figure 3d) show that the added electron, while fully delocalized in the π-system, has relatively more density on N5, N11, and C13, which may hint at possible decomposition pathways.^35^

### Mass Transport and Electrokinetics of **5a**

The electrochemical kinetics of ROMs, including the diffusion coefficient (*D*) and the heterogeneous electron-transfer kinetics (*k*_0_), are critical for the performance of RFBs. We investigated both *D* and *k*_0_ of **5a** through two experimental methods: CV with varying scan rates from 25 to 500 mV/s (Figure 4c) and linear sweep voltammetry (LSV) experiment (Figure 4d). The peak current observed in the CV exhibited a linear relationship with the square root of the scan rate under different scan rates (Figure 4c), indicating a diffusion-limited redox process for our model compound. The Randles–Sevcik equation was employed to estimate the diffusion coefficient of **5a**, resulting in an approximate value of 1.1 × 10^–5^ cm^2^/s (see the Supporting Information, including Figure SI17). To determine the accurate diffusion coefficient (*D*) and the heterogeneous electron-transfer kinetics (*k*_0_) of **5a**,^55^ we conducted an LSV experiment using a three-electrode rotating disk electrode (RDE) setup with a glassy carbon working RDE (Figure 4d). The LSV analysis revealed a linear relationship between the limiting current and the square root of the angular speed (Figure 4d inset); the derived slope was applied to calculate the diffusion coefficient (*D*). The diffusion coefficient of **5a** was determined to be 1.7 × 10^–5^ cm^2^/s using the Levich equation, which is higher than that of other low-potential anolytes such as *N*-methylphthalimide (8.4 × 10^–6^ cm^2^/s).^56^ Further analysis was performed using Koutecky–Levich plots (Figure 4f) based on the LSV data. The linear Tafel plot was derived as a function of overpotential (Figure 4e inset) and employed the Butler–Volmer equation to determine the heterogeneous electron-transfer constant (*k*_0_) of **5a**. The obtained *k*_0_ value of **5a** was 1.5 × 10^–3^ cm/s (see the Supporting Information, including Table SI3 and Figures SI21 and SI22), which is comparable to that of *N*-methylphthalimide (2.5 × 10^–3^ cm/s).^56^ Notably, the Nicholson method yielded an estimated heterogeneous electron-transfer constant of **5a** as approximately 5.0 × 10^–3^ cm/s (see the Supporting Information, Table SI1 and Figure SI18), which significantly differs from the result obtained through the LSV test.^55^ The determined diffusion coefficient and rate constant demonstrate that the new scaffold possesses good kinetic properties.

### Static H-Cell Cycling Stability of **5a**

Electrochemical cycling stability is a crucial parameter for ROMs, and we evaluated the cycling stability of **5a** by performing galvanostatic charge–discharge experiments (see the Supporting Information for further details). In these experiments, 5 mL of 5 mM solution of **5a** in 0.5 M TBAPF_6_/MeCN is placed in the working electrode side of an H-cell separated with a fine glass fit. Initially, the counter electrode side of the cell contains 5 mL of 0.5 M TBAPF_6_/MeCN. **5a** is reduced to **5a**^•–^ at a constant rate (−5 mA) to a voltage limit of 350 mV lower than the *E*_1/2_ of **5a** to maximize the state of charge (SOC), and then the current is reversed (+5 mA) to regenerate **5a**. This cycling was repeated to evaluate the stability of **5a**. As shown in Figure 4f, **5a** exhibited only a 9.18% capacity fade over 202 cycles (46.8 h) with an average Coulombic efficiency of 100% throughout the cycling. We also compared the cycling stability of quinoxaline (**8**) and phenazine (**9**) using the same assay (Figure 4d). Phenazine (**9**) exhibited a 26.3% capacity fade over 202 cycles (35.8 h), while quinoxaline (**8**) exhibited a 50.4% capacity fade after only 12 cycles (2.0 h). After 202 cycles (5.1 h), the capacity fade of quinoxaline (**8**) increased to 98.6%. These results demonstrate that incorporating the electron-rich trivalent π-donor *N*-atom into the expanded conjugation system can indeed significantly improve cycling stability while simultaneously pushing the reduction potential closer to the edge of the reduction window of acetonitrile.

### Study of the Relationship between the Structure of Indolo[2,3-*b*]quinoxaline Derivatives and Their Stability and Solubility

**Figure 5:**
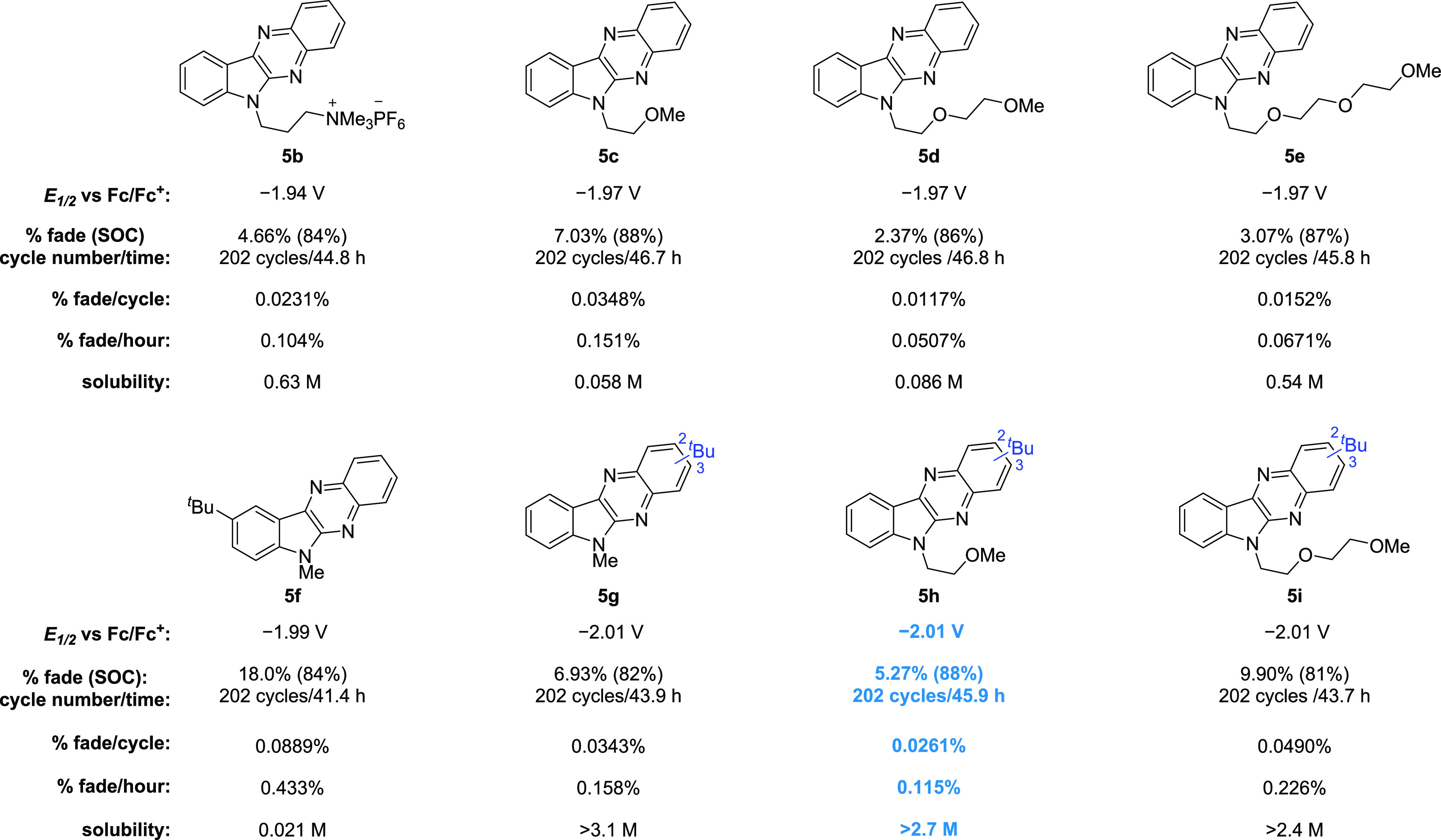
Evaluation of reduction potential, H-cell cycling stability, and solubility of indolo[2,3-*b*]quinoxaline derivatives **5b**–**5i**.

Encouraged by these results, we proceeded to study the structure–property relationships for this class of anolytes in order to identify next-generation derivatives based on the indolo[2,3-*b*]quinoxaline scaffold, with a focus on enhancing solubility and H-cell cycling stability. Previous studies have suggested the use of tetraalkylammonium groups and glycol ethers to enhance the solubility of ROMs.^57^ We first incorporated the solubilizing groups into the core through *N*-alkylation. Ammonium salt (**5b**) and glycol ether derivatives **5c–e** were synthesized through our convergent synthetic route in excellent yield (91–93%; see the SI). Incorporating tetramethylammonium groups significantly improved the solubility of **5b** to 0.63 M in acetonitrile, which is almost 20-fold greater than that of **5a** (Figure 5). The electron-withdrawing property of the ammonium salt resulted in a 30 mV positive shift in the reduction potential of **5b** to −1.94 V vs Fc/Fc^+^. The cycling stability of **5b** improved, showing a 4.66% capacity fade over 202 cycles (44.8 h). This improvement may be due to stabilizing charge–charge interactions between the ammonium salt and the radical anion. The different lengths of glycol ether substitutions did not affect the reduction potential, which remained at −1.97 V vs Fc/Fc^+^ for **5c**, **5d**, and **5e** (Figure 5). However, the solubility of glycol ether derivatives showed a positive correlation with the number of glycol ether units (Figure 5). The stability also benefited from the glycol ether substitution and **5d** proved optimal, showing just a 2.37% capacity fade over 202 cycles (46.8 h).

We reasoned that the introduction of a sterically bulky substituent on the ring could interrupt the π–π stacking of the conjugated π-system and lead to further improved solubility. Specifically, we were drawn to the *tert*-butyl group, which we believed could act as both an electron-donating group and a solubilizing group by interrupting the π–π stacking and which might benefit both the reduction potential and solubility. To test this, we synthesized the 9-*tert*-butyl-substituted derivative **5f** and the mixture of 2- and 3-*tert*-butyl-substituted **5g**. Benefiting from the electron-donation property of *tert*-butyl, the reduction potential of **5f** moved 20 mV negative to −1.99 V vs Fc/Fc^+^ and **5g** moved 40 mV to −2.01 V vs Fc/Fc^+^ (Figure 5). CV studies showed that 2-*tert*-butyl or 3-*tert*-butyl substitution results in the same electrochemical properties on the CV scale. As the mixture of 2- and 3-*tert*-butyl substitution should increase system entropy and therefore benefit solubility as compared to pure regioisomers, we did not try to separate the mixture to obtain the single isomers. To our surprise, the solubility of **5f** dropped to 0.021 M in acetonitrile compared with **5a**. However, the solubility of **5g** dramatically increased to over 3.1 M in acetonitrile, which could be attributed to the *tert*-butyl effect and the mixture of 2-*tert*-butyl and 3-*tert*-butyl substitution derivatives. Static H-cell cycling experiments reveal that the 9-*tert*-butyl substitution decreased the cycling stability of the indolo[2,3-*b*]quinoxaline core. **5f** exhibits only half the stability of **5a**, with a capacity fade of 18.0% over 202 cycles (41.4 h). However, interestingly, the mixture of 2- and 3-*tert*-butyl substitution improved stability in addition to enhancing potential performance. **5g** exhibited a capacity fade of only 6.93% over 202 cycles (43.9 h).

With the knowledge gained from these structure–property studies, we aimed to incorporate the solubilizing and stabilizing groups together in a single scaffold. As such, we synthesized two derivatives, **5h** and **5i**, with 2- and 3-*tert*-butyl substitution and glycol ether side chains. Both **5h** and **5i** exhibit the same reduction potential as **5g** (−2.01 V vs Fc/Fc^+^) as well as high solubility (>2.7 and >2.4 M, respectively, in acetonitrile). H-cell cycling experiments showed that the stability of **5h** (5.27% capacity fade over 202 cycles (45.9 h)) is better than that of **5i** (9.90% capacity fade over 202 cycles (43.7 h)) and **5g** (6.93% capacity fade over 202 cycles (43.9 h)), which is in line with the previous observed glycol ether unit effect trend for the indolo[2,3-*b*]quinoxaline core (i.e., **5d** vs **5e** and **5c**). Interestingly, the DFT calculations reveal that the π-system is further expanded via hyperconjugation with orbitals from both the *tert*-butyl and *N*6-substitution side chain (Figure SI29). We further investigated the electrochemical kinetics of **5h** using both LSV and CV. The diffusion coefficient (*D*) was 1.2 × 10^–5^ cm^2^/s, and the kinetic rate constant (*k*_0_) was 9.6 × 10^–4^ cm/s through LSV analysis (see the Supporting Information, including Table SI4 and Figures SI23–SI26). Both of these values are comparable to other ROMs used in NARFBs.^38,43,56^

### Identification of the Catholyte

**Figure 6:**
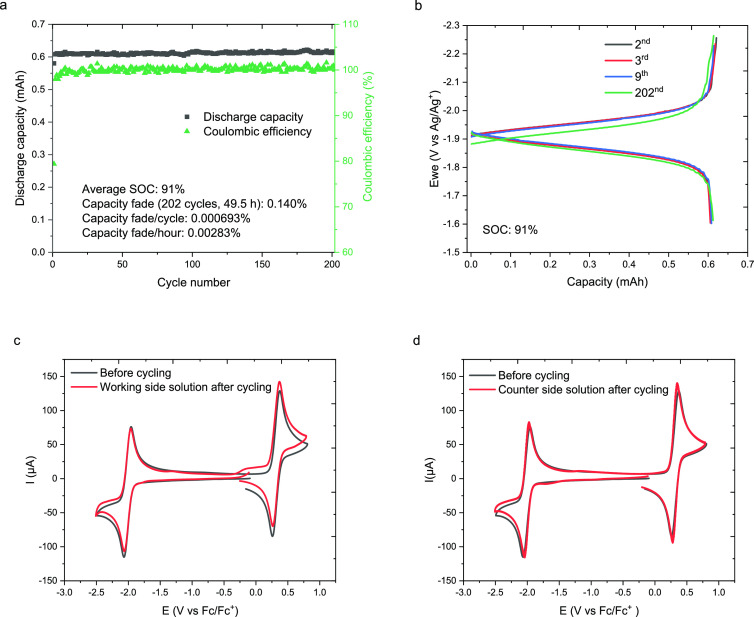
(a) Discharge capacity and Coulombic efficiency versus cycle number of mixed 5 mM **5h** and 5 mM **10** solution (in 0.5 M TBAPF_6_/MeCN) in a static H-cell cycling. (b) Potential versus capacity for the 2nd, 3rd, 9th, and 202nd H-cell cycling (91% SOC). CV of the anolyte (c) and catholyte (d) before and after H-cell cycling between **5h** and **5h**^•–^of mixed 5 mM **5h** and 5 mM **10** solution (5 mM in 0.5 M TBAPF_6_/MeCN) with a glassy carbon working electrode at a scan rate of 100 mV/s.

With the optimal anolyte **5h** in hand, we sought to test its performance in a flow battery. To do so, we first needed to identify a compatible catholyte. We explored the phenothiazine derivative MEEPT (**10**),^24^ which has a first oxidation potential of 0.32 V vs Fc/Fc^+^ and exhibits high cycling stability and solubility, as a potential catholyte. To initially assess the compatibility between **5h** and **10**, a mixed solution of 5 mM **5h** and 5 mM **10** in 0.5 M TBAPF_6_/MeCN was subjected to a static H-cell cycling experiment where **5h** is cycled with **5h**^•–^, while **10** remained electrochemically neutral throughout. The anolyte **5h** displays improved cycling stability in the mixed solution as measured by the discharge capacity and Coulombic efficiency of cycling between **5h** and **5h**^•–^ shown in Figure 6a, with 99.86% capacity retention (0.140% capacity fading) after 202 cycles (49.5 h). This equates to a 0.000693% capacity fade per cycle and a 0.0283% capacity fade per hour, making it among the most stable anolytes reported in acetonitrile.^19,20,58−62^ The 202nd cycle shows almost the same SOC (91%) and similar charge–discharge performance as the 9th cycle (Figure 6b). The post-cycling CV analysis revealed almost identical CV curves, with no new peaks observed nor any significant amplitude decrease in either the working (Figure 6c) or the counter side of the H-cell setup (Figure 6d). Furthermore, the mixture displayed similarly exceptional performance when **10** was cycled between its neutral state and its radical cation **10**^•+^ with **5h** remaining electrochemically neutral (Figure SI13).

### Flow Battery Cycling of **5h** with **10**

**Figure 7:**
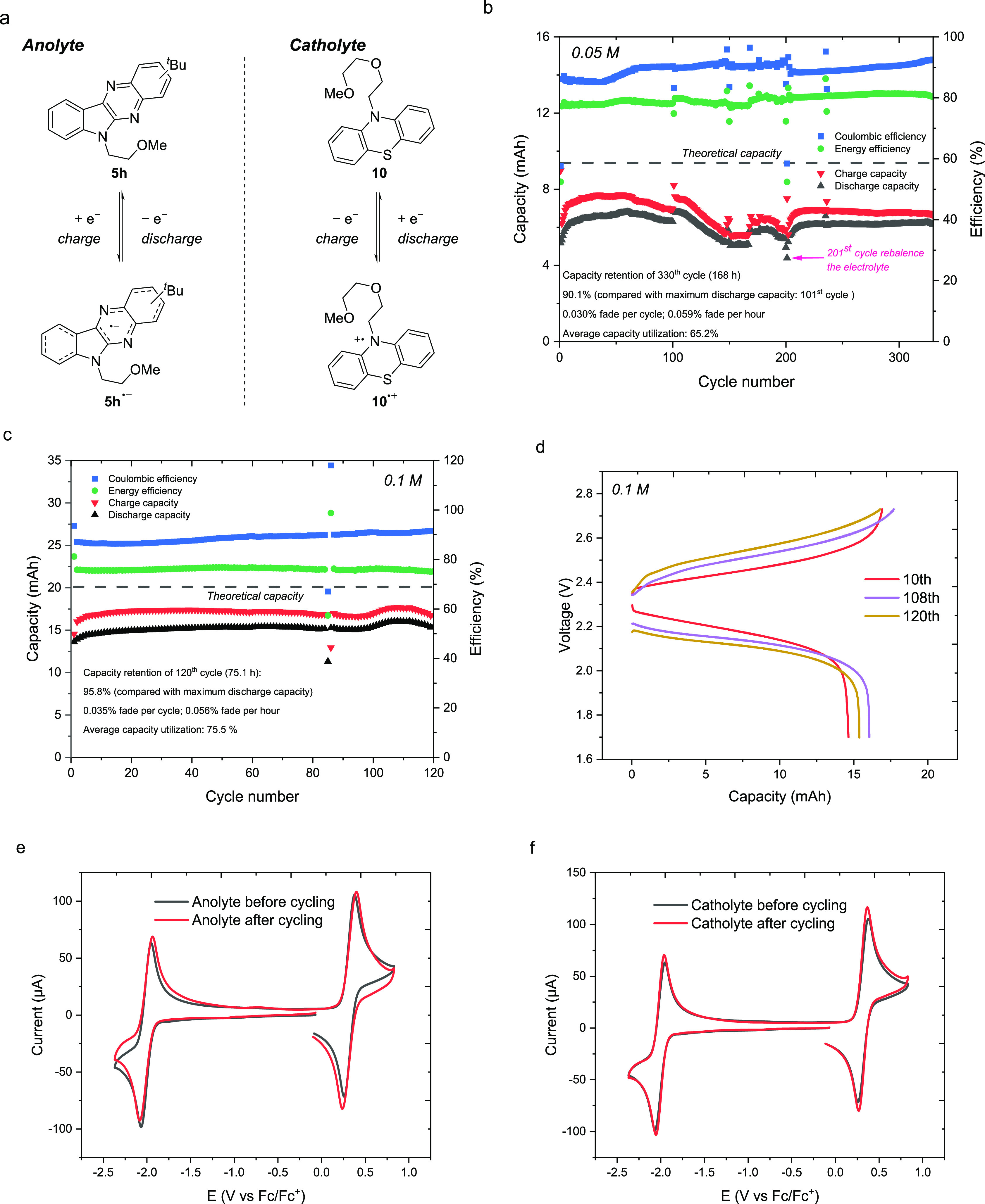
(a) Anolyte and catholyte electrochemical reactions in the flow battery of **5h** and **10**. (b) Charge capacity, discharge capacity, Coulombic efficiency, and energy efficiency versus cycle number for flow cell cycling of 50 mM **5h** and 50 mM **10** (c) and 100 mM **5h** and 100 mM **10** (d) in 0.5 M TBAPF_6_/MeCN solution. CVs of the 5 mM diluted anolyte (e) and catholyte (f) before and after cycling the flow battery (100 mM **5h** and 100 mM **10** in 0.5 M TBAPF_6_/MeCN solution) with a glassy carbon working electrode at a scan rate of 100 mV/s.

Finally, we evaluated the performance of **5h** as an anolyte in a flow cell battery with **10** as the catholyte using a mixed electrolyte solution of **5h** and **10**. During the charge–discharge process, the electrochemical reaction for this flow battery system is depicted in Figure 7a. The flow battery was assembled with a mesoporous membrane (Daramic AA-175), and both the anolyte and catholyte reservoirs were filled with a mixed solution of 50 mM **5h** and 50 mM **10** in 0.5 M TBAPF_6_/MeCN (7.0 mL each side) to limit the potential impact of crossover driven by a concentration gradient. The electrolyte solutions were charged and discharged at a constant current density of 10 mA/cm^2^ until reaching cutoff voltages of 2.68 and 1.98 V (±350 mV from the theoretical cell potential) at a flow rate of 10 mL/min. The capacity utilization reached 66.1% in the steady flat state after initial equilibrium by the tenth cycle (Figure 7b). The Coulombic efficiency was 85.5%, and the energy efficiency was 77.5% for the 10th cycle. After 200 cycles, the flow cell was rebalanced to recover the discharge capacity (∼80% retention) caused by volume discrepancies in the electrolyte.^58^ Then, the battery underwent another 200 cycles with the same parameters. The discharge capacity remained 90.1% after 330 cycles over 168 h compared to the maximum discharge capacity (101st cycle; 72.8% peak capacity utilization). However, the discharge capacity significantly decreased after 330 cycles, reaching only 31.0% by the 400th cycle (Figure SI14), with substantial volume discrepancy in the reservoirs, presumably arising from the differences of pressure/viscosity of the anolyte and catholyte that result in preferential mass transfer from the anolyte to the catholyte solution.^44,58,59,61,62^ Impedance analysis conducted on the flow cell post-400 cycles showed an increase in resistance (Figure SI14).^62^ Post-cycling CV analysis of diluted electrolyte solutions (100 μL diluted to 1.0 mL) revealed no significant concentration change of the ROMs in either the anolyte or catholyte side of the cell (Figure SI14), suggesting that the capacity loss was mainly due to the battery failing to charge and not as a result of ROM decomposition.

Encouraged by these results, we proceeded to test a higher concentration (100 mM) of the mixed **5h** and **10** electrolyte solution in 0.5 M TBAPF_6_/MeCN. The electrolyte solutions (7.5 mL per side) were charged and discharged at a constant current density of 20 mA/cm^2^ until reaching cutoff voltages of 2.73 and 1.70 V at a flow rate of 20 mL/min. In this case, the capacity utilization reaches 72.8% after the initial equilibrium at the tenth cycle, achieving a steady flat state (Figure 7c), while the Coulombic efficiency is 86.6% and the energy efficiency is 75.6% for the 10th cycle. Remarkably, the discharge capacity of the 120th cycle remained at 95.8% compared to the maximum discharge capacity (108th cycle; 79.9% peak capacity utilization) over 75.1 h cycling with 75.5% average capacity utilization. However, the charge capacity of the battery decreased dramatically afterward, with the discharge capacity reaching only 28.9% by the 200th cycle (Figure SI15). Impedance analysis conducted on the flow cell revealed a substantial increase in resistance after the 200th cycle.^63^ Post-200 cycles, CV analysis of diluted electrolyte solutions (50 μL diluted to 1.0 mL) indicated that there was less than a 5% concentration loss in either the anolyte or catholyte. The flow cell battery was rebalanced after 200 cycles, and the battery was run for another 20 cycles with the same parameters, with the capacity recovering to 91.7% at the 205th cycle (Figure SI15). These observations suggest that the capacity loss was mainly due to the buildup of charge and/or concentration gradients in the cell that create flow cell resistance and which result in prematurely reaching the cell cutoff potential and not as a result of the loss of the ROM. Rebalancing the cell alleviates these gradients and allows for a return to higher discharge capacities. The battery demonstrated extraordinary capacity retention compared with other reported high-performance NARFBs.^44,58−62^ We further conducted the flow cycling of the mixed **5h** and **10** electrolyte solution at a higher concentration (250 mM; Figure SI16). However, the material utilization and battery system are likely to be constrained by the ion viscosity and diffusivity in the 0.5 M TBAPF_6_/MeCN electrolyte.^58^ Overall, the results of this study show the potential of **5h** as a highly promising anolyte for NARFBs with competitive solubility, reduction potential, and cycling performance.

## Conclusions

In summary, we have designed indolo[2,3-*b*]quinoxaline as a new anolyte scaffold for NARFBs. Our approach involved expanding the conjugated π-system with a trivalent π-donor nitrogen atom fusion and incorporating the mixed *tert*-butyl compound as a design principle that could potentially be applied to other material designs. To identify structure–property relationships, we synthesized a library of indolo[2,3-*b*]quinoxaline derivatives through a convergent synthetic route and evaluated their electrochemical properties and solubilities. Among these derivatives, 2- and 3-*tert-*butyl-6-(2-methoxyethyl)-6*H*-indolo[2,3-*b*]quinoxaline (**5h**) was identified as the best anolyte due to its low reduction potential (*E*_1/2_ = −2.01 V vs Fc/Fc^+^), high stability (0.000693% capacity fade/cycle; H-cell cycling), and high solubility (greater than 2.7 M in acetonitrile). **5h** paired with MEEPT (**10**) was deployed to a prototype flow battery, achieving a 2.3 V battery with a 2.68 Ah/L capacity. The battery maintained 95.8% capacity retention (120 cycles; 75 h) and demonstrated an average utilization of 75.5%.

## Supporting Information Available

Experimental procedures and compound characterization (PDF)
