# Supplementary material for: Indolo[2,3-b]quinoxaline as a Low Reduction Potential and High Stability Anolyte Scaffold for Nonaqueous Redox Flow Batteries
Source: J Am Chem Soc. 2023 Aug 16;145(34):18877–87. doi: 10.1021/jacs.3c05210 (PMC10472437; doi:10.1021/jacs.3c05210)
Supplement: Supplementary file 1 — ja3c05210_si_001.pdf [file ja3c05210_si_001.pdf]

Electronic Supporting Information

Indolo[2,3-*b*]quinoxaline as a low reduction potential and high stability anolyte scaffold for non-aqueous redox flow batteries

Wenhao Zhang,[†,‡,§] Ryan Walser-Kuntz,[ǁ,§] Jacob S. Tracy,[†,‡,§] Tim K. Schramm,[‡,¶] James Shee,[‡] Martin Head-Gordon,[†,‡] Gan Chen,[†] Brett A. Helms,[†] Melanie S. Sanford,[ǁ,§] and F. Dean Toste[*,†,‡,§]

[†]Chemical Science Division, Lawrence Berkeley National Laboratory, 1 Cyclotron Road, Berkeley, California 94720, United States

[‡]Department of Chemistry, University of California, Berkeley, California 94720, United States

[ǁ]Department of Chemistry, University of Michigan, Ann Arbor, Michigan 48109, United States

[§]Joint Center for Energy Storage Research (JCESR), 9700 South Cass Avenue, Argonne, Illinois, 60439, USA

[¶]Department of Chemistry, RWTH Aachen University, Landoltweg 1, Aachen 52074, Germany

*Correspondence to: fdtoste@berkeley.edu

Table of contents

I. General Information S2

II. Experimental Procedures and Spectroscopic Data of Compounds S2

III. ^{1}H and ^{13}C NMR Spectra for Compounds S8

IV. Electrochemical Experiments Details, Materials, and Methods, Including Photographs of Experimental Setup S34

V. Electrochemical Characterization Data S36

VI. Experiments Details and Methods of Electrochemical Kinetics Studies S53

VII. Comparison of the Cell Performance of This Work with Other State-of-Art NARFBs Anolytes S62

VIII. Density Functional Theory Calculations S67

IX. References S72

I. General Information

All reactions were carried out in flame-dried glassware sealed with rubber septa, under a nitrogen atmosphere with dry solvents under anhydrous conditions, unless otherwise noted. Dimethylformamide (DMF) used for synthesis was obtained by passing these previously degassed solvents through activated alumina columns under argon. Reagents were purchased at the highest commercial quality and used without further purification unless otherwise stated. Solvents for chromatography were purchased from Sigma-Aldrich. Reactions were monitored by thin layer chromatography (TLC) carried out on S-2 0.25 mm E. Merck silica gel plates (60F-254) using UV light for visualizing and aqueous ammonium cerium nitrate or basic aqueous potassium permanganate as developing agent. E. Merck silica gel (60, particle size 0.040–0.063 mm) was used for flash column chromatography. All NMR spectra were recorded at the University of California, Berkeley NMR facility. NMR spectra were recorded on Bruker AV-400 and AV-600 instruments. The spectra were calibrated by using residual undeuterated solvents (for ^{1}H NMR) and deuterated solvents (for ^{13}C NMR) as internal references: chloroform (δ_H = 7.26 ppm), and $CDCl_3$ (δ_C = 77.16 ppm); acetonitrile (δ_H = 1.94 ppm) and acetonitrile-d$_3$ (δ_C = 1.32 ppm); acetone (δ_H = 2.05 ppm) and acetone-d$_6$ (δ_C = 29.84 ppm). The following abbreviations are used to designate multiplicities: s = singlet, d = doublet, t = triplet, q = quartet, m = multiplet, quint = quintet, br = broad. IR spectra were recorded on a Bruker Vertex 80 FTIR spectrometer. High-resolution mass spectra (HRMS) were recorded on a Perkin Elmer UHPLC-TOF (ESI) at the Lawrence Berkeley National Laboratory Catalysis Laboratory located in the Department of Chemistry at the University of California, Berkeley.

II. Experimental Procedures and Spectroscopic Data of Compounds

6-methyl-*6H*-indolo[2,3-*b*]quinoxaline (5a):

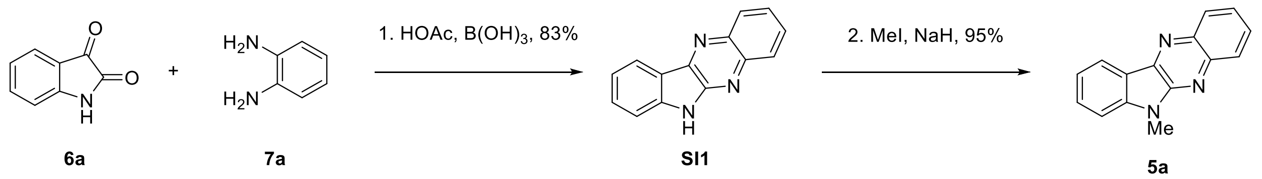

Step 1: To a stirred solution of isatin (**6a**, 3.67 g, 25.0 mmol) in acetic acid (70.0 mL) was added *O*-phenylenediamine (**7a,** 2.70 g, 25.0 mmol) and boronic acid (1.85 g, 30.0 mmol) at 22 °C, the resultant mixture was allowed to stir at that temperature for 15 h before the filter. The yellow solid was obtained by filtration, followed by washing with acetic acid (3 × 10 mL) and ethyl ether (3 × 10 mL). The filtrate was concentrated under vacuum, and water (30 mL) was added to the residue. The mixture was filtered, and the solid was sequentially washed with water (3 × 10 mL) and cold acetone (3.0 mL). The combined solid was dry under vacuum, giving **SI1** (4.56 g, 83%) as a yellow solid. **SI1**: R_f = 0.25 (silica gel, ethyl acetate:hexane 1:2); IR (film): ν_{max} = 3069, 1617, 1461, 1408, 1246, 1137, 753, 746 cm^{-1}; ^{1}H NMR (400

MHz, acetone-d_6): δ = 8.40 (d, *J* = 7.8 Hz, 1 H), 8.26 (d, *J* = 8.4 Hz, 1 H), 8.07 (d, *J* = 8.4 Hz, 1 H), 7.83–7.75 (m, 1 H), 7.75–7.68 (m, 2 H), 7.65 (d, *J* = 8.1 Hz, 1 H), 7.40 (dd, *J* = 7.5, 7.5 Hz, 1 H) ppm; ^{13}C NMR (151 MHz, acetone-d_6): δ = 147.00, 144.90, 141.69, 141.05, 140.33, 132.02, 130.27, 129.44, 128.66, 126.77, 123.09, 121.76, 120.77, 112.72 ppm; HRMS (*m/z*): $[M + H]^+$ calcd for $C_{14}H_{10}N_3^+$ 220.0869, found 220.0868.

Step 2: To a stirred mixture of **SI1** (110 mg, 0.500 mmol) in DMF (500 μL) was added sodium hydride (40.0 mg, 60% *wt*, 1.00 mmol) at 0 °C. The resultant mixture was allowed to stir at that temperature for 5 mins before MeI (94 μL, 1.50 mmol) was added. The mixture was warmed to 22 °C and stirred for 5 h before it was quenched with saturated aq. $NaHCO_3$ (5.0 mL) and diluted with EtOAc (20 mL) and brine (20 mL). The resultant mixture was extracted with EtOAc (3 × 10 mL). The combined organic phases were washed with brine (50 mL), dried over anhydrous Na_2SO_4, and filtered. The volatiles were removed under vacuum, and the residue was purified by flash column chromatography with ethyl acetate/hexane (1:30 → 1:10) to give **5a** (111 mg, 95%) as a yellow solid. **5a**: R_f = 0.27 (silica gel, ethyl acetate:hexane 1:4); IR (film): ν_{max} = 1613, 1586, 1494, 1473, 1253, 1118, 746 cm^{-1}; ^{1}H NMR (400 MHz, $CDCl_3$): δ = 8.46 (d, *J* = 7.7 Hz, 1 H), 8.30 (d, *J* = 8.3 Hz, 1 H), 8.13 (d, *J* = 8.3 Hz, 1 H), 7.80–7.73 (m, 1 H), 7.73–7.64 (m, 2 H), 7.43 (d, *J* = 8.1 Hz, 1 H), 7.38 (dd, *J* = 7.5, 7.5 Hz, 1 H), 3.95 (s, 3 H) ppm; ^{13}C NMR (151 MHz, $CDCl_3$): δ = 145.99, 145.13, 140.71, 140.27, 139.38, 131.13, 129.52, 128.93, 127.81, 126.06, 122.78, 121.08, 119.53, 109.29, 27.65 ppm; HRMS (*m/z*): $[M + Na]^+$ calcd for $C_{15}H_{11}N_3Na^+$ 256.0845, found 250.0834.

3-(*6H*-indolo[2,3-*b*]quinoxalin-6-yl)-*N,N,N*-trimethylpropan-1-aminium hexafluorophosphate(V) (5b):

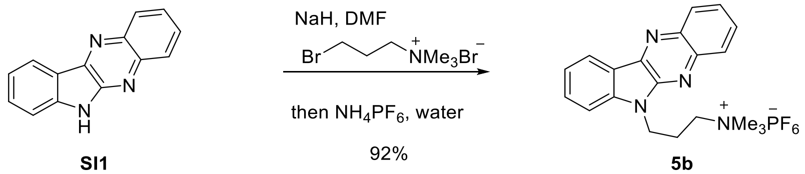

SI1 **5b**

To a stirred mixture of **SI1** (1.10 g, 5.00 mmol) in DMF (10.0 mL) was slowly added sodium hydride (240 mg, 60% *wt*, 6.00 mmol) at 0 °C. (*Caution*: Adding sodium hydride slowly, hydrogen is generated). The resultant mixture was allowed to stir at that temperature for 10 mins before 3-bromo-*N,N,N*-trimethylpropan-1-aminium bromide (1.33 g, 6.00 mmol) was added. The mixture was warmed to 22 °C and stirred for 5 h before quenched with water. The resultant mixture was subject to a vacuum to remove the volatiles at 40 °C before water (10 mL) was added to the resultant mixture. NH_4PF_6 (1.63 g, 10.0 mmol) in 15.0 mL water was added to the resultant mixture at 22 °C. The resultant mixture was allowed to stir at that temperature for 1 h before extracted with CH_2Cl_2 (3 × 50 mL). The combined organic phases were washed with brine (100 mL), dried over anhydrous Na_2SO_4, and filtered. The volatiles were removed under vacuum, and the residue was purified by recrystallization with acetone and ethyl ether (1:10) to give **5b** (2.14 g, 92%) as a yellow solid. **5b**: IR (film): ν_{max} = 1586, 1491, 1471, 1415, 1120, 836, 751, 558 cm^{-1}; ^{1}H NMR (400 MHz, CD_3CN): δ = 8.38 (d, *J* = 7.6 Hz, 1 H), 8.25 (d, *J* = 8.1 Hz, 1 H), 8.10 (d, *J* = 8.0 Hz, 1 H), 7.84–7.76 (m, 2 H), 7.77–7.77 (m, 1 H), 7.66 (d, *J* = 8.1 Hz, 1 H), 7.43 (dd, *J* = 7.5, 7.5 Hz, 1 H), 4.57 (t, *J* = 6.4 Hz, 2 H), 3.45–3.31 (m, 2 H), 2.94 (s, 9 H), 2.49–2.39 (m, 2 H) ppm; ^{13}C NMR (151 MHz, CD_3CN): δ = 146.89, 145.26, 141.40, 141.33, 140.29, 132.40, 130.29, 129.98, 128.77, 127.19, 123.22, 122.30, 120.62, 110.95, 65.23, 53.98, 53.95, 53.92, 39.17, 23.33 ppm; ^{19}F NMR (377 MHz, CD_3CN): δ = –72.72 (d, *J* = 706.5 Hz, PF_6); HRMS (*m/z*): $[M]^+$ calcd for $C_{20}H_{23}N_4^+$ 319.1917, found 319.1912.

6-(2-methoxyethyl)-*6H*-indolo[2,3-*b*]quinoxaline (5c):

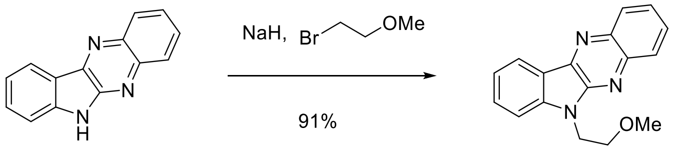

To a stirred mixture of **SI1** (87.7 mg, 0.400 mmol) in DMF (500 μL) was added sodium hydride (24.0 mg, 60% *wt*, 0.600 mmol) at 0 °C. The resultant mixture was allowed to stir at that temperature for 5 mins before 1-bromo-2-methoxyethane (75.0 μL, 0.800 mmol) was added. The mixture was warmed to 22 °C and stirred for 5 h before it was quenched with saturated aq. $NaHCO_3$ (5.0 mL), and diluted with EtOAc (10 mL) and brine (10 mL). The resultant mixture was extracted with EtOAc (3 × 10 mL). The combined organic phases were washed with brine (50 mL), dried over anhydrous Na_2SO_4, and filtered. The volatiles were removed under vacuum, and the residue was purified by flash column chromatography with ethyl acetate/hexane (1:12 → 1:4) to give **5c** (101 mg, 91%) as a yellow solid. **5c**: R_f = 0.38 (silica gel, ethyl acetate:hexane 1:2); IR (film): ν_{max} = 2932, 1485, 1469, 1410, 1354, 1193, 1119, 748 cm^{-1}; ^{1}H NMR (400 MHz, $CDCl_3$): δ = 8.47 (d, *J* = 7.8 Hz, 1 H), 8.31 (dd, *J* = 8.3, 1.5 Hz, 1 H), 8.14 (dd, *J* = 8.3, 1.5 Hz, 1 H), 7.76 (ddd, *J* = 8.4, 6.8, 1.6 Hz, 1 H), 7.73–7.65 (m, 2 H), 7.59 (d, *J* = 8.2 Hz, 1 H), 7.43–7.34 (m, 1 H), 4.67 (t, *J* = 5.6 Hz, 2 H), 3.91 (t, *J* = 5.7 Hz, 2 H), 3.34 (s, 3 H) ppm; ^{13}C NMR (151 MHz, $CDCl_3$): δ = 145.85, 145.10, 140.72, 140.33, 139.54, 131.10, 129.49, 128.84, 127.96, 126.13, 122.71, 121.09, 119.62, 110.26, 70.86, 59.22, 41.71 ppm; HRMS (*m/z*): [M + H]$^+$ calcd for $C_{17}H_{16}N_3^+$ 300.1107, found 300.1110.

6-(2-(2-methoxyethoxy)ethyl)-*6H*-indolo[2,3-*b*]quinoxaline (5d):

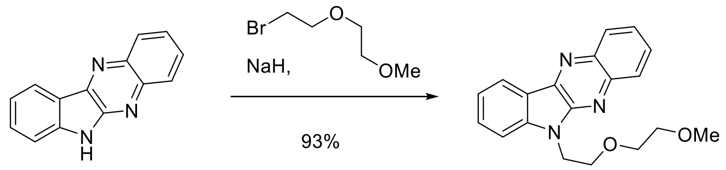

To a stirred mixture of **SI1** (110 mg, 0.500 mmol) in DMF (500 μL) was added sodium hydride (40.0 mg, 60% *wt*, 1.00 mmol) at 0 °C. The resultant mixture was allowed to stir at that temperature for 5 mins before 1-bromo-2-(2-methoxyethoxy)ethane (81 μL, 1.50 mmol) was added. The mixture was warmed to 22 °C and stirred for 5 h before it was quenched with saturated aq. $NaHCO_3$ (5.0 mL), then diluted with EtOAc (10 mL) and brine (10 mL). The resultant mixture was extracted with EtOAc (3 × 10 mL). The combined organic phases were washed with brine (50 mL), dried over anhydrous Na_2SO_4, and filtered. The volatiles were removed under vacuum, and the residue was purified by flash column chromatography with ethyl acetate/hexane (1:10 → 1:3) to give **5d** (149 mg, 93%) as a dark orange oil. **5d**: R_f = 0.32 (silica gel, ethyl acetate:hexane 1:1); IR (film): ν_{max} = 2877, 1584, 1469, 1410, 1356, 1204, 1118, 748 cm^{-1}; ^{1}H NMR (400 MHz, $CDCl_3$): δ = 8.46 (d, *J* = 7.8 Hz, 1 H), 8.30 (dd, *J* = 8.3, 1.4 Hz, 1 H), 8.12 (dd, *J* = 8.4, 1.3 Hz, 1 H), 7.75 (ddd, *J* = 8.4, 6.9, 1.5 Hz, 1 H), 7.71–7.65 (m, 2 H), 7.63 (d, *J* = 8.2 Hz, 1 H), 7.38 (ddd, *J* = 8.0, 7.0, 1.0 Hz, 1 H), 4.70 (t, *J* = 5.9 Hz, 2 H), 4.00 (t, *J* = 5.9 Hz, 2 H), 3.66–3.56 (m, 2 H), 3.47–3.39 (m, 2 H), 3.28 (s, 3 H) ppm; ^{13}C NMR (151 MHz, $CDCl_3$): δ = 145.82, 145.09, 140.70, 140.33, 139.53, 131.03, 129.49, 128.84, 127.93, 126.12, 122.65, 121.07, 119.61, 110.42, 72.06, 70.78, 69.41, 59.16, 41.67 ppm; HRMS (*m/z*): [M + Na]$^+$ calcd for $C_{19}H_{19}N_3O_2Na^+$ 344.1369, found 344.1364.

6-(2-(2-(2-methoxyethoxy)ethoxy)ethyl)-*6H*-indolo[2,3-*b*]quinoxaline (5e):

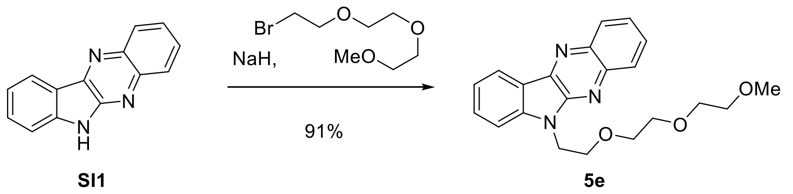

To a stirred solution of **SI1** (439 mg, 2.00 mmol) in DMF (4.0 mL) was added sodium hydride slowly (120 mg, 60% *wt*, 3.00 mmol) at 0 °C. The resultant mixture was allowed to stir at that temperature for 5 mins before 1-bromo-2-(2-(2-methoxyethoxy)ethoxy)ethane (545 μL, 2.40 mmol) was added. The mixture was warmed to 22 °C and stirred for 5 h before it was quenched with saturated aq. $NaHCO_3$ (10 mL), then diluted with EtOAc (20 mL) and brine (20 mL). The resultant mixture was extracted with EtOAc (3 × 20 mL). The combined organic phases were washed with brine (100 mL), dried over anhydrous Na_2SO_4, and filtered. The volatiles were removed under vacuum, and the residue was purified by flash column chromatography with ethyl acetate/hexane (1:5 → 1:1) to give **5e** (665 mg, 91%) as a dark orange oil. **5e**: R_f = 0.18 (silica gel, ethyl acetate:hexane 1:1); IR (film): ν_{max} = 2877, 1585, 1470, 1411, 1355, 1204, 1119, 751 cm^{-1}; ^1H NMR (400 MHz, $CDCl_3$): δ = 8.46 (d, *J* = 7.7 Hz, 1 H), 8.30 (dd, *J* = 8.2, 1.5 Hz, 1 H), 8.12 (dd, *J* = 8.3, 1.4 Hz, 1 H), 7.75 (ddd, *J* = 8.4, 6.9, 1.5 Hz, 1 H), 7.72–7.65 (m, 2 H), 7.62 (d, *J* = 8.2 Hz, 1 H), 7.37 (ddd, *J* = 8.0, 7.0, 1.1 Hz, 1 H), 4.69 (t, *J* = 5.7 Hz, 2 H), 3.99 (t, *J* = 5.7 Hz, 2 H), 3.67–3.58 (m, 2 H), 3.57–3.50 (m, 2 H), 3.49–3.43 (m, 2 H), 3.41–3.35 (m, 2 H), 3.30 (s, 3 H) ppm; ^{13}C NMR (151 MHz, $CDCl_3$): δ = 145.81, 145.11, 140.69, 140.31, 139.51, 131.00, 129.48, 128.84, 127.92, 126.11, 122.63, 121.04, 119.58, 110.46, 71.97, 70.94, 70.73, 70.66, 69.41, 59.09, 41.72 ppm; HRMS (*m/z*): $[M + Na]^+$ calcd for $C_{21}H_{23}N_3O_3Na^+$ 365.1739, found 388.1630.

9-(*tert*-butyl)-6-methyl-6H-indolo[2,3-*b*]quinoxaline (5f):

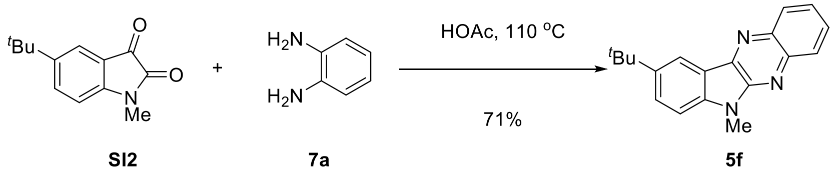

To a stirred solution of **SI2** (217 mg, 1.00 mmol) in acetic acid (1.0 mL) was added *O*-phenylenediamine (**7a**, 108 mg, 1.00 mmol). The resultant mixture was heated to 110 °C and stirred for 3 h before cooling to 22 °C. The volatiles were removed under vacuum, and the residue was purified by flash column chromatography with ethyl acetate/hexane (1:20 → 1:8) to give **5f** (206 mg, 71%) as a yellow solid. **5f**: R_f = 0.38 (silica gel, ethyl acetate:hexane 1:4); IR (film): ν_{max} = 2961, 1583, 1482, 1390, 1267, 811, 758 cm^{-1}; ^1H NMR (400 MHz, $CDCl_3$): δ = 8.52 (d, *J* = 2.0 Hz, 1 H), 8.31 (dd, *J* = 8.3, 1.5 Hz, 1 H), 8.14 (dd, *J* = 8.3, 1.4 Hz, 1 H), 7.81–7.72 (m, 2 H), 7.68 (ddd, *J* = 8.3, 6.8, 1.5 Hz, 1 H), 7.41 (d, *J* = 8.5 Hz, 1 H), 3.96 (s, 3 H), 1.47 (s, 9 H) ppm; ^{13}C NMR (151 MHz, $CDCl_3$): δ = 146.36, 144.49, 143.35, 140.67, 140.64, 139.23, 129.44, 129.01, 128.82, 127.78, 125.97, 119.30, 119.22, 108.90, 35.07, 32.00, 27.70 ppm; HRMS (*m/z*): $[M + H]^+$ calcd for $C_{19}H_{20}N_3^+$ 290.1652, found 290.1660.

2/3-(*tert*-butyl)-6-methyl-*6H*-indolo[2,3-*b*]quinoxaline (5g):

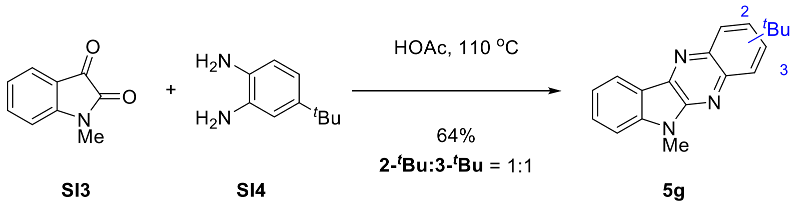

To a stirred solution of 1-methylindoline-2,3-dione (**SI3**, 484 mg, 3.00 mmol) in acetic acid (2.0 mL) was added 4-(*tert*-butyl)benzene-1,2-diamine (**SI4,** 492.8 mg, 3.00 mmol). The resultant mixture was heated to 110 °C and stirred for 3 h before cooling to 22 °C. The volatiles were removed under vacuum, and the residue was purified by flash column chromatography with ethyl acetate/hexane (1:20 → 1:8) to give **5g** (558 mg, 64%) as a yellow solid. **5g**: R_f = 0.39 (silica gel, ethyl acetate:hexane 1:4); IR (film): ν_{max} = 2963, 1586, 1475, 1397, 1256, 1124, 830, 746 cm^{-1}; ^{1}H NMR (400 MHz, $CDCl_3$): δ = 8.52–8.44 (m, 1 H), 8.29–8.21 (m, 1 H), 8.13–8.05 (m, 1 H), 7.87 (dd, *J* = 8.9, 2.3 Hz, 0.5 H), 7.78 (dd, *J* = 8.9, 2.2 Hz, 0.5 H), 7.74–7.66 (m, 1 H), 7.48–7.42 (m, 1 H), 7.42–7.35 (m, 1 H), 4.06–3.92 (m, 3 H), 1.50 (s, 9 H) ppm; ^{13}C NMR (151 MHz, $CDCl_3$): δ = 152.52, 149.36, 146.18, 145.98, 144.98, 144.86, 140.57, 140.01, 139.71, 139.21, 138.96, 137.73, 130.92, 130.81, 128.83, 127.93, 127.16, 125.08, 124.82, 123.19, 122.62, 122.62, 121.01, 120.96, 119.69, 119.59, 109.28, 109.26, 35.43, 35.20, 31.54, 31.48, 27.69, 27.66 ppm; HRMS (*m/z*): $[M + H]^+$ calcd for $C_{19}H_{20}N_3^+$ 290.1652, found 290.1653.

2/3-(*tert*-butyl)-6-(2-methoxyethyl)-*6H*-indolo[2,3-*b*]quinoxaline (5h):

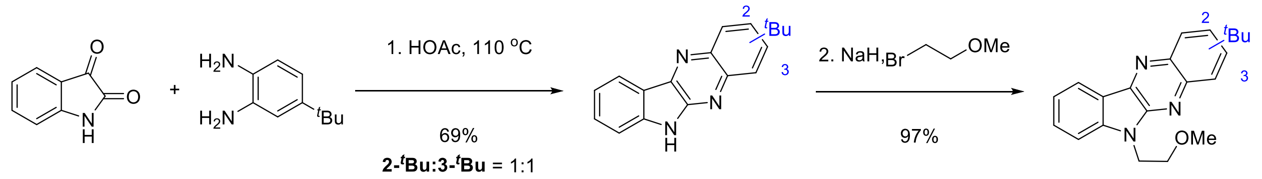

5a **SI4** **SI5** **5h**

Step 1: To a stirred solution of **5a** (1.47 g, 10.0 mmol) in acetic acid (1.0 mL) was added 4-(*tert*-butyl)benzene-1,2-diamine (**SI4,** 1.64 g, 10.0 mmol). The resultant mixture was heated to 110 °C and stirred for 3 h before cooling to 25 °C. The volatiles were removed under vacuum, and the residue was purified by flash column chromatography with ethyl acetate/dichloromethane/hexane (1:1:10 → 1:1:6) to give **SI5** (1.90 g, 69%) as an orange solid. **SI5**: R_f = 0.41;0.35 (silica gel, ethyl acetate:hexane 1:4); IR (film): ν_{max} = 3143, 2963, 1619, 1494, 1404, 1341, 1181, 825, 728 cm^{-1}; ^{1}H NMR (400 MHz, $CDCl_3$): δ = 10.10 (brs, 1 H), 8.54–8.44 (m, 1 H), 8.33–8.25 (m, 1 H), 8.15–8.09 (m, 1 H), 7.96–7.80 (m, 1 H), 7.71–7.64 (m, 1 H), 7.63–7.56 (m, 1 H), 7.44–7.37 (m, 1 H), 1.59–1.45 (m, 9 H) ppm; ^{13}C NMR (151 MHz, $CDCl_3$): δ = 152.80, 149.92, 146.23, 146.02, 143.14, 143.00, 140.46, 140.12, 139.58, 138.52, 138.09, 131.18, 131.06, 128.99, 128.24, 126.71, 125.59, 124.99, 122.86, 122.69, 121.54, 121.52, 120.46, 120.37, 111.66, 111.64, 35.47, 35.27, 31.52, 31.45 ppm; HRMS (*m/z*): $[M + H]^+$ calcd for $C_{18}H_{18}N_3^+$ 276.1495, found 276.1508.

Step 2: To a stirred mixture of **SI5** (550.7 mg, 2.00 mmol) in DMF (1.0 mL) was slowly added sodium hydride (96.0 mg, 60% *wt*, 2.40 mmol) at 0 °C. The resultant mixture was allowed to stir at that temperature for 5 mins before 1-bromo-2-methoxyethane (226 *μ*L, 2.40 mmol) was added. The mixture was warmed to 22 °C and stirred for 5 h before it was quenched with saturated aq. $NaHCO_3$ (10.0 mL), then diluted with EtOAc (20 mL) and brine (20 mL). The resultant mixture was extracted with EtOAc (3 × 20 mL). The combined organic phases were washed with brine (50 mL), dried over anhydrous Na_2SO_4, and filtered. The volatiles were removed under vacuum, and the residue was purified by flash column chromatography with ethyl acetate/hexane (1:20 → 1:6) to give **5h** (646 mg, 97%) as an orange half solid. **5h**: R_f = 0.30 (silica gel, ethyl acetate:hexane 1:4); IR (film): ν_{max} = 2961, 1585, 1469, 1404, 1355, 1179, 1123, 748 cm^{-1}; ^{1}H NMR (400 MHz, $CDCl_3$): δ = 8.52–8.41 (m, 1 H), 8.27 (d, *J* = 2.2 Hz, 0.5 H), 8.23 (d, *J* = 8.9 Hz, 0.5 H), 8.12–8.05 (m, 1 H), 7.86 (dd, *J* = 8.9, 2.2 Hz, 0.5 H), 7.78 (dd, *J* = 8.9, 2.2 Hz, 0.5 H), 7.70–7.63 (m, 1 H), 7.57 (d, *J* = 8.2 Hz, 1 H), 7.40–7.34 (m, 1 H), 4.73–4.59 (m, 2 H), 3.96–3.84 (m, 2 H), 3.33 (s, 1.5 H), 3.32 (s, 1.5 H), 1.49 (s, 9 H) ppm; ^{13}C NMR (151 MHz, $CDCl_3$): δ = 152.34, 149.38, 145.98, 145.76, 144.86, 144.73, 140.56, 140.02, 139.69, 139.34, 138.92, 137.86, 130.84, 130.72, 128.78, 127.80, 127.27, 125.10, 124.76, 123.32, 122.49, 122.49, 120.96, 120.93, 119.73, 119.64, 110.17, 110.15,

70.85, 59.23, 59.19, 41.67, 35.41, 35.18, 31.52, 31.47 ppm; HRMS (*m/z*): $[M + H]^+$ calcd for $C_{21}H_{24}N_3O^+$ 334.1914, found 334.1910.

2/3-(*tert*-butyl)-6-(2-(2-methoxyethoxy)ethyl)-*6H*-indolo[2,3-*b*]quinoxaline (5i):

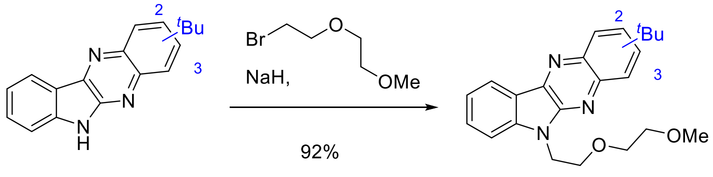

To a stirred mixture of **SI5** (275 mg, 1.00 mmol) in DMF (1.0 mL) was added sodium hydride (44.0 mg, 60% *wt*, 1.10 mmol) at 0 °C. The resultant mixture was allowed to stir at that temperature for 5 mins before 1-bromo-2-(2-methoxyethoxy)ethane (162 μL, 1.20 mmol) was added. The mixture was warmed to 22 °C and stirred for 5 h before it was quenched with saturated aq. $NaHCO_3$ (5.0 mL), then diluted with EtOAc (10 mL) and brine (10 mL). The resultant mixture was extracted with EtOAc (3 × 10 mL). The combined organic phases were washed with brine (50 mL), dried over anhydrous Na_2SO_4, and filtered. The volatiles were removed under vacuum, and the residue was purified by flash column chromatography with ethyl acetate/hexane (1:10 → 1:4) to give **5i** (348 mg, 92%) as a dark yellow oil. **5i**: R_f = 0.30 (silica gel, ethyl acetate:hexane 1:2); IR (film): ν_{max} = 2961, 2872, 1584, 1492, 1469, 1406, 1124, 831, 748 cm^{-1}; ^1H NMR (400 MHz, $CDCl_3$): δ = 8.51–8.41 (m, 1 H), 8.26 (d, *J* = 2.2 Hz, 0.5 H), 8.23 (d, *J* = 8.8 Hz, 0.5 H), 8.12–8.03 (m, 1 H), 7.85 (dd, *J* = 8.9, 2.2 Hz, 0.5 H), 7.78 (dd, *J* = 8.9, 2.2 Hz, 0.5 H), 7.69–7.64 (m, 1 H), 7.64–7.59 (m, 1 H), 7.40–7.32 (m, 1 H), 4.77–4.62 (m, 2 H), 4.05–3.93 (m, 2 H), 3.64–3.54 (m, 2 H), 3.47–3.36 (m, 2 H), 3.28 (s, 1.5 H), 3.27 (s, 1.5 H), 1.49 (s, 9 H) ppm; ^{13}C NMR (151 MHz, $CDCl_3$): δ = 152.35, 149.39, 145.95, 145.74, 144.88, 144.78, 140.55, 140.03, 139.71, 139.33, 138.92, 137.85, 130.78, 130.66, 128.78, 127.80, 127.26, 125.10, 124.76, 123.29, 122.45, 122.44, 120.95, 120.92, 119.72, 119.63, 110.35, 110.35, 72.04, 70.80, 70.76, 69.44, 69.41, 59.14, 41.66, 35.40, 35.18, 31.52, 31.47 ppm; HRMS (*m/z*): $[M + H]^+$ calcd for $C_{23}H_{28}N_3O_2^+$ 378.2176, found 378.2176.

III ^{1}H and ^{13}C NMR Spectra of Compounds

^{1}H NMR Spectrum of SI1 (400 MHz, acetone-d_6)

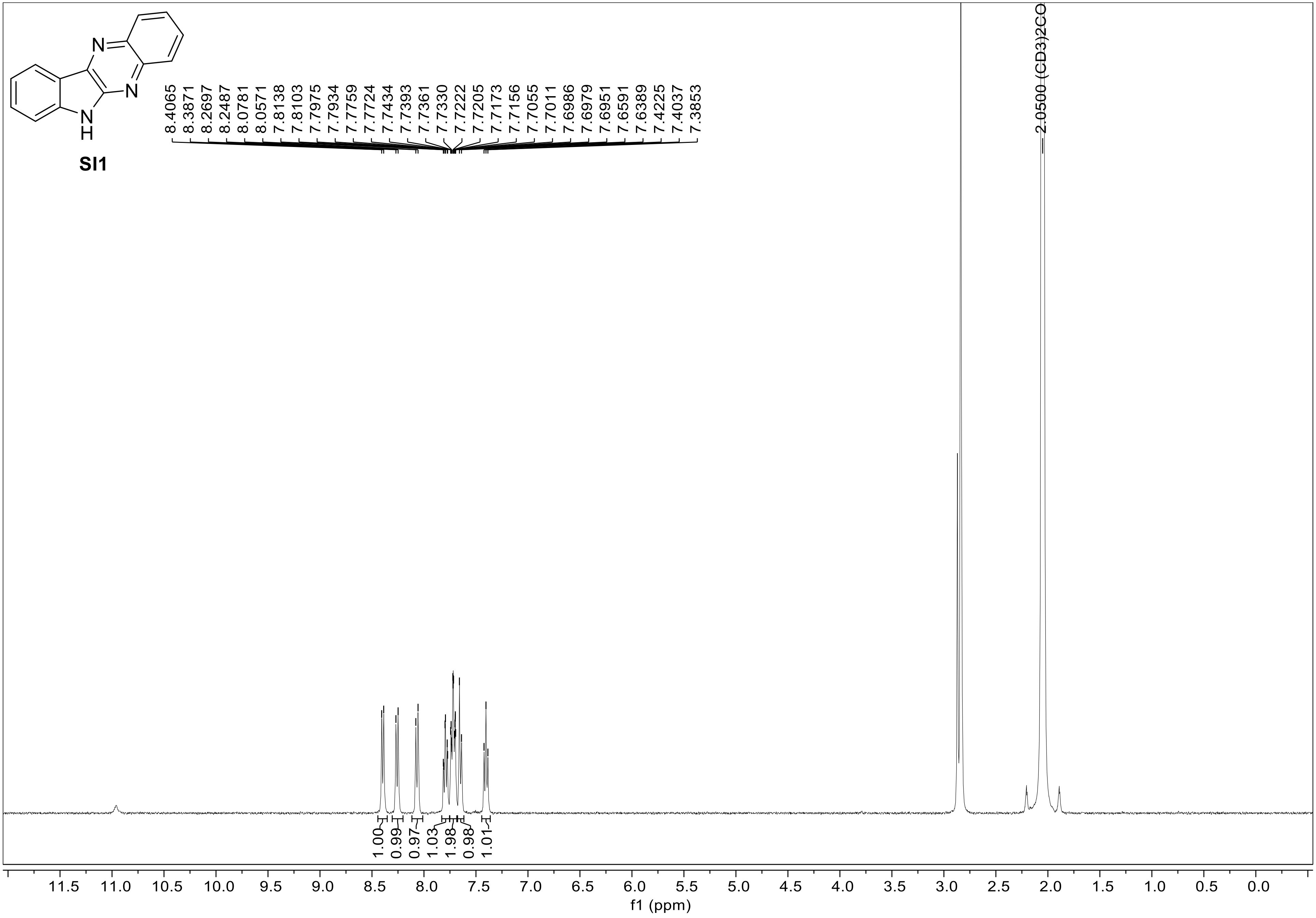

^{13}C NMR Spectrum of SI1 (151 MHz, acetone-d_6)

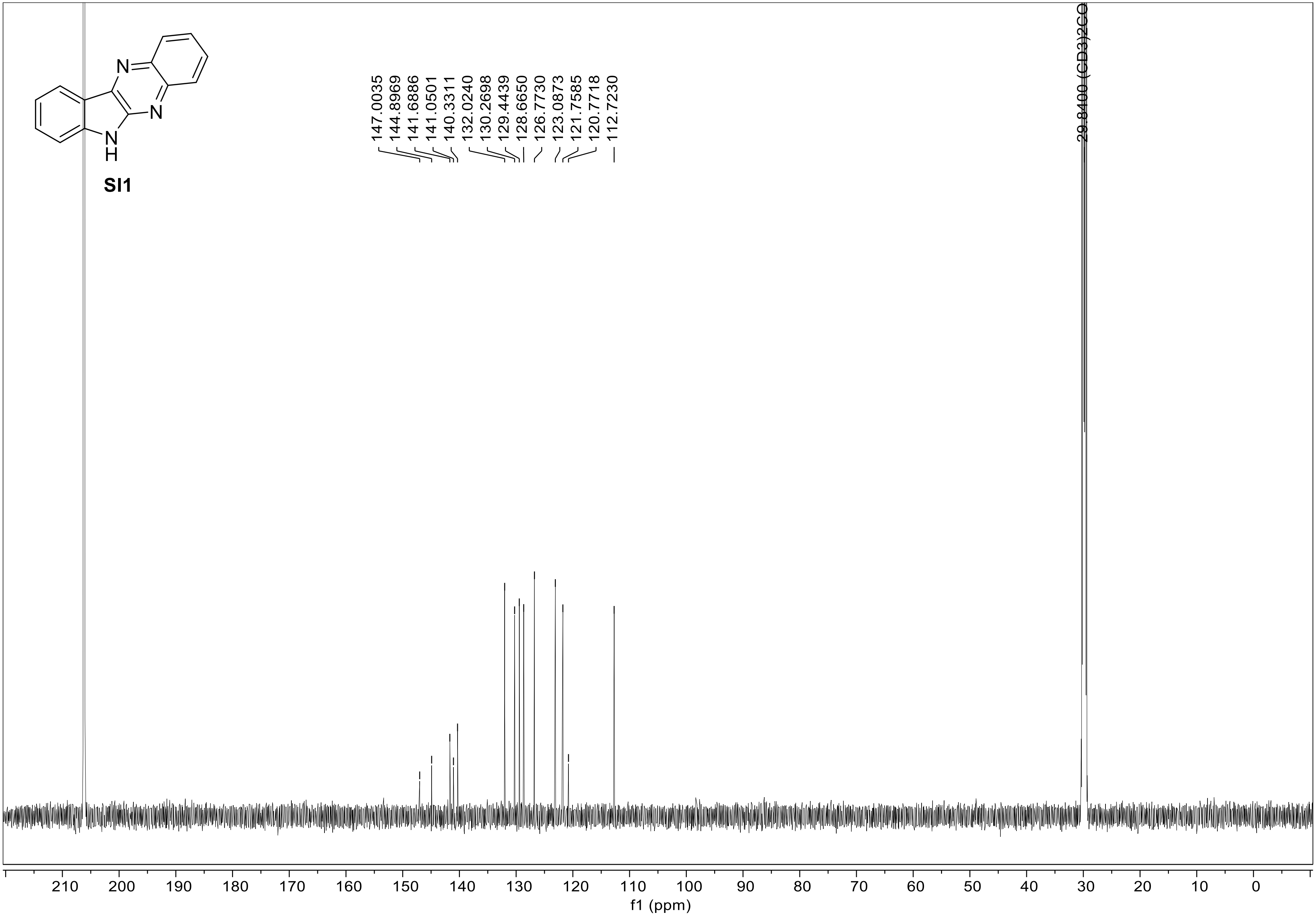

^1H NMR Spectrum of 5a (400 MHz, $CDCl_3$)

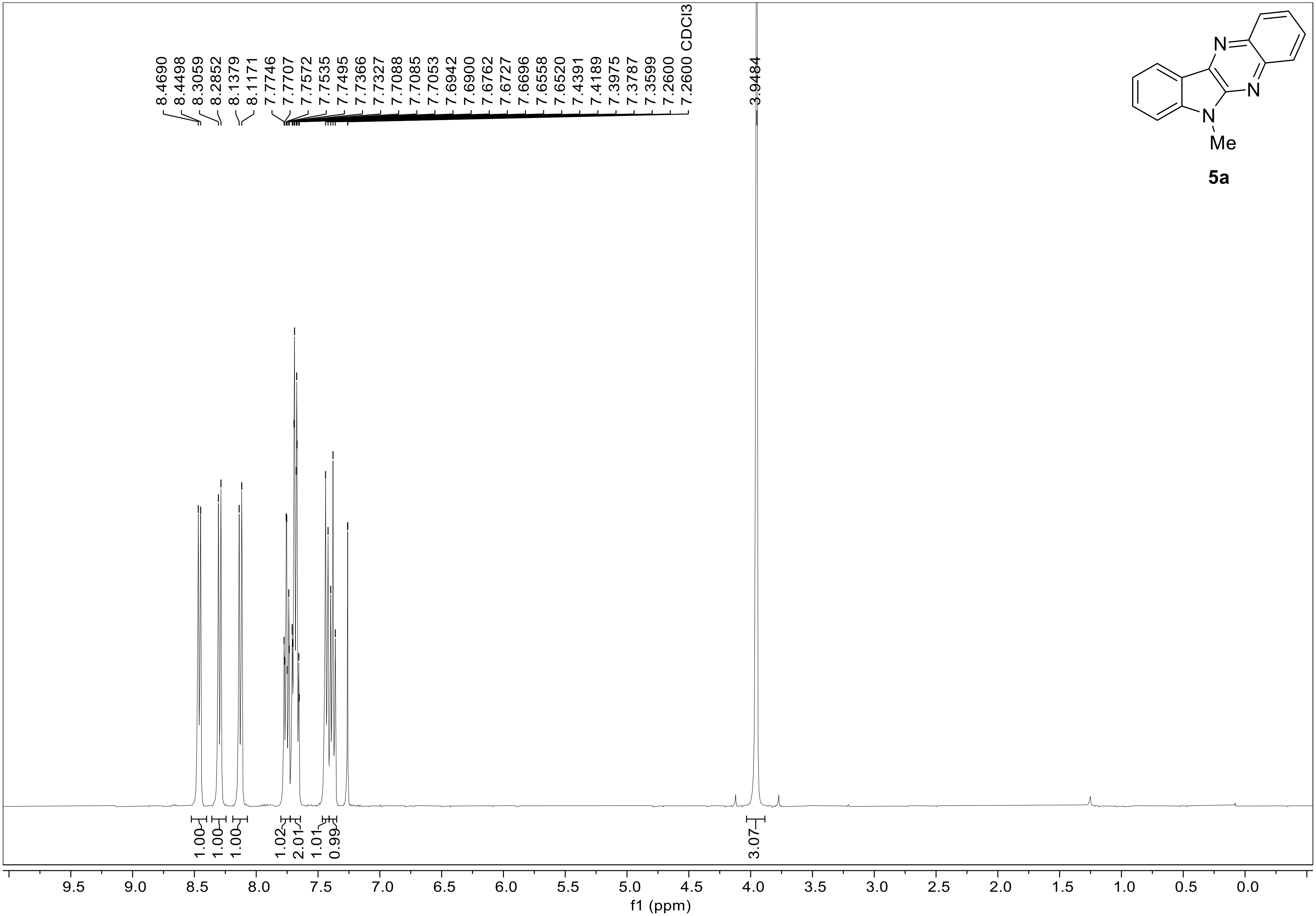

^{13}C NMR Spectrum of 5a (151 MHz, $CDCl_3$)

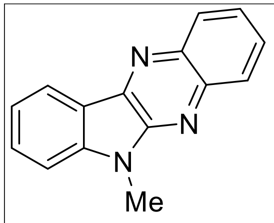

5a

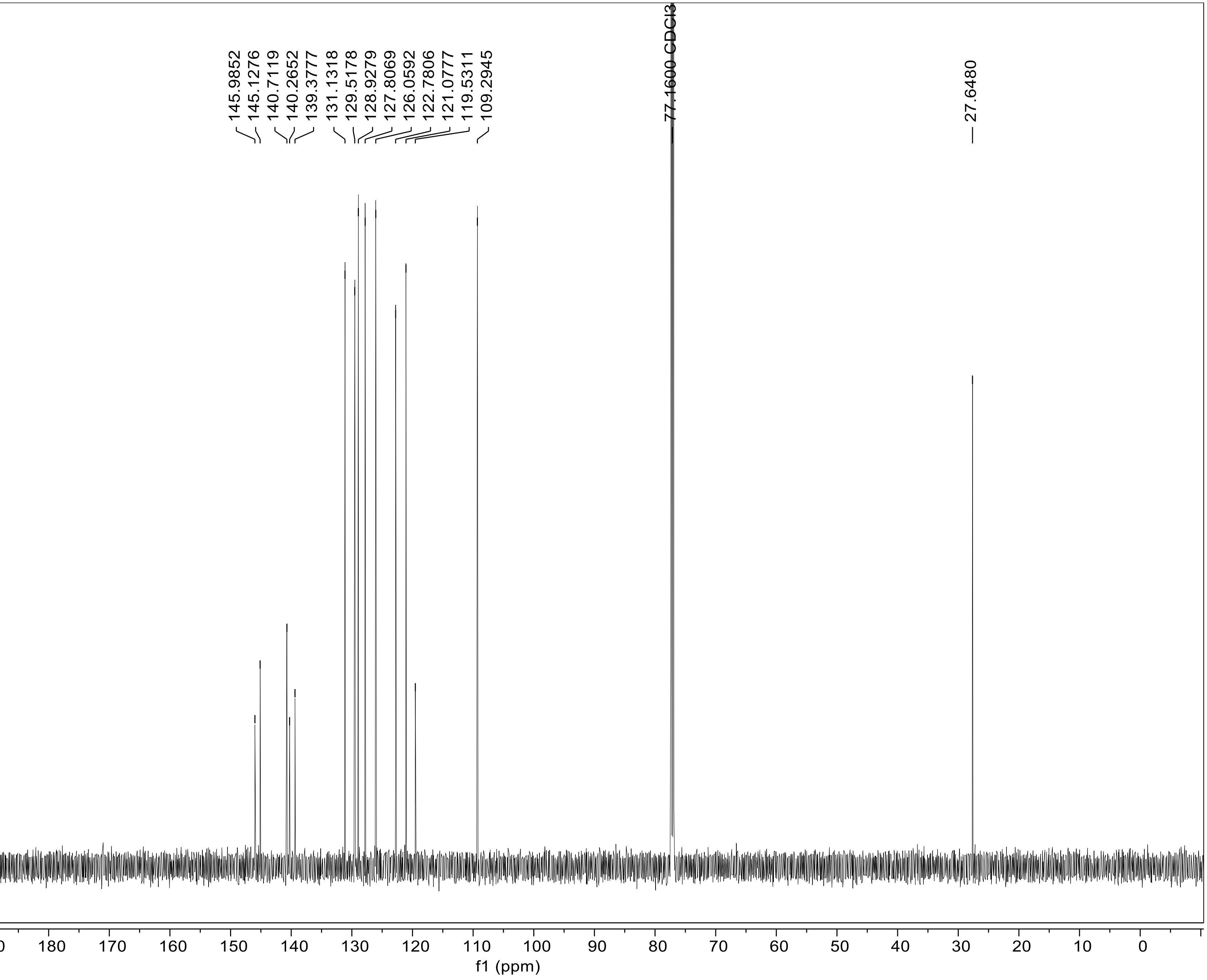

^{1}H NMR Spectrum of 5b (400 MHz, CD_3CN)

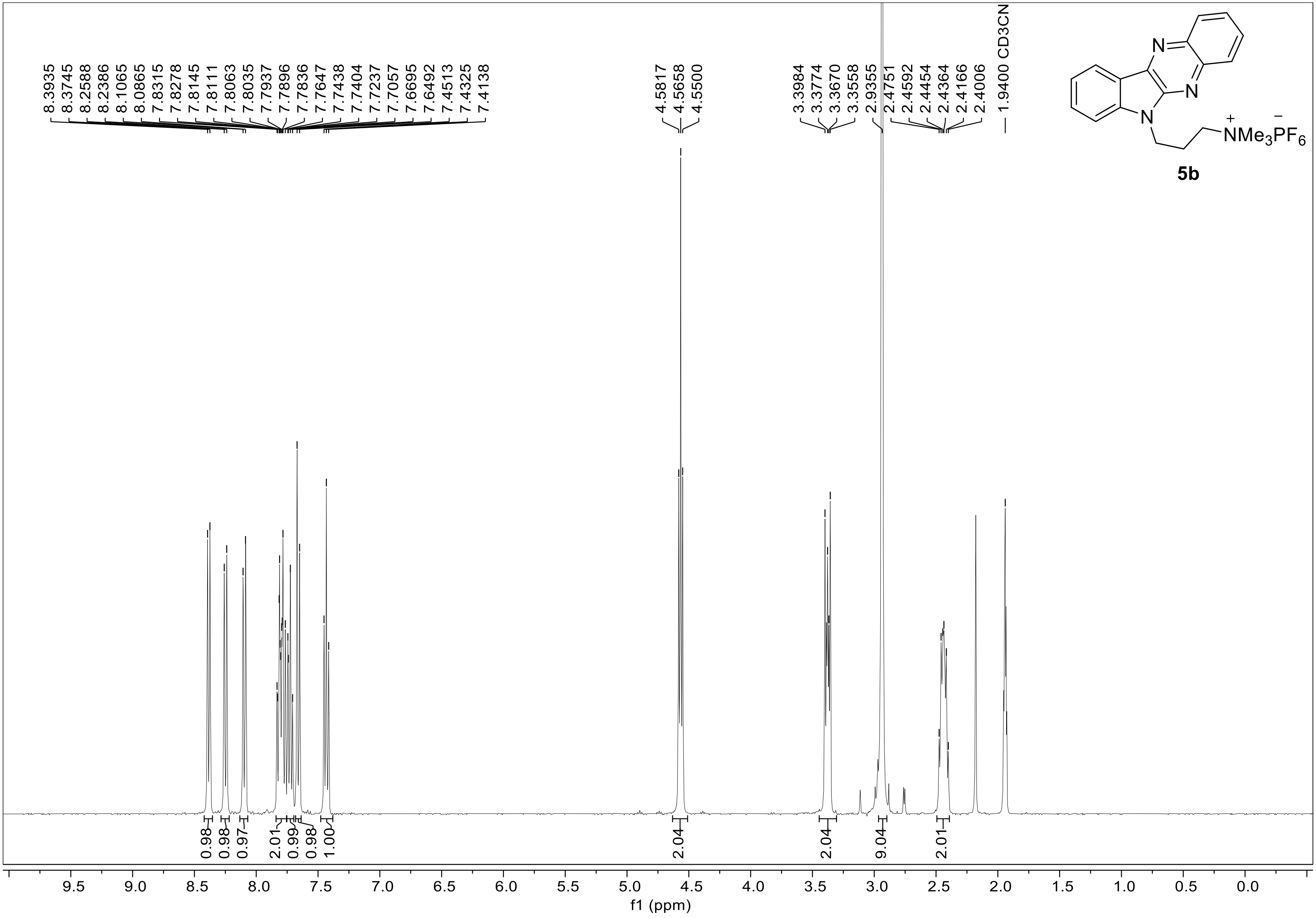

^{13}C NMR Spectrum of 5b (151 MHz, CD_3CN)

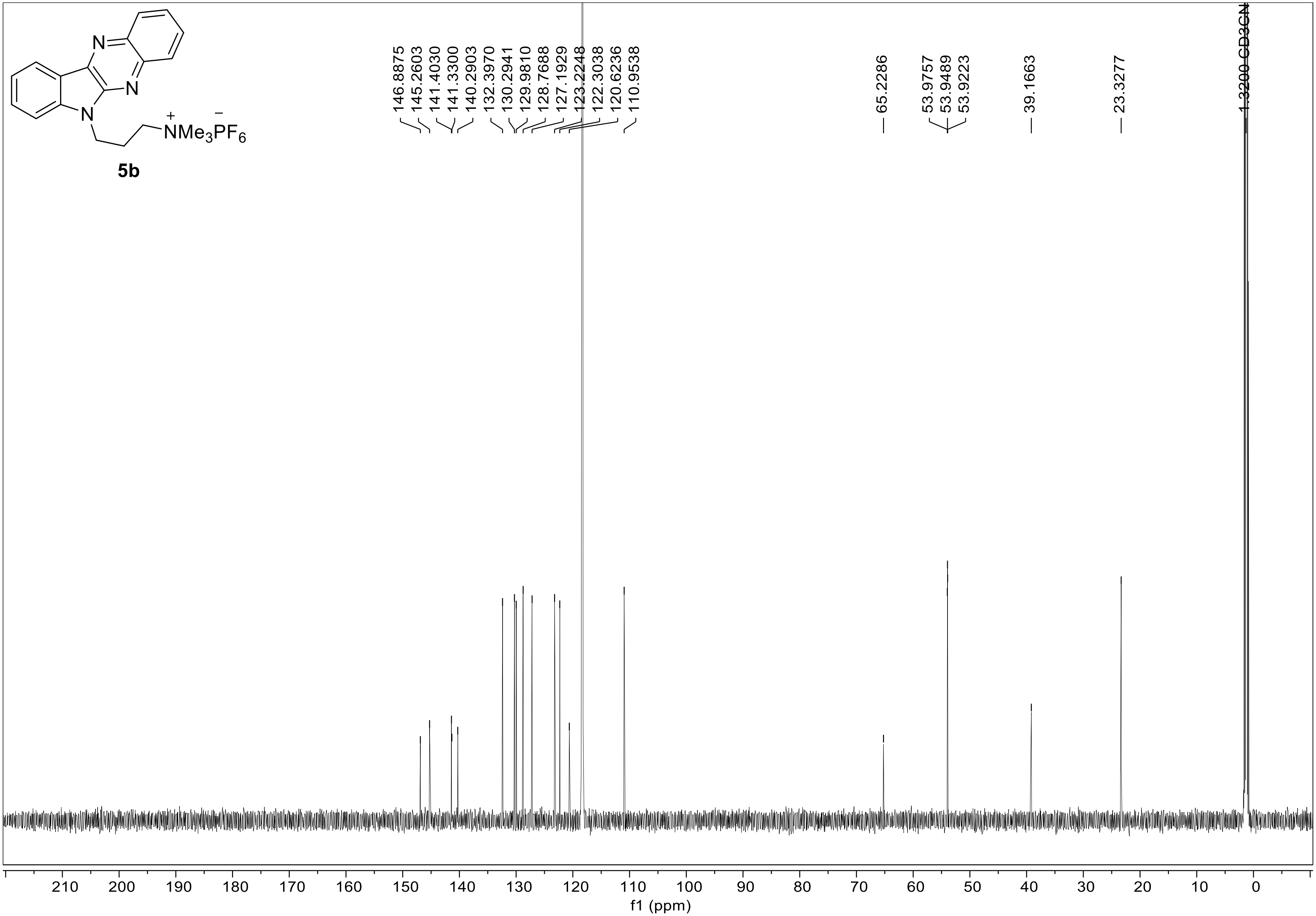

^{19}F NMR Spectrum of 5b (377 MHz, $CDCl_3$)

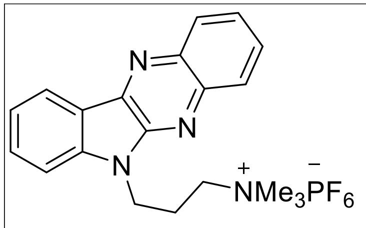

5b

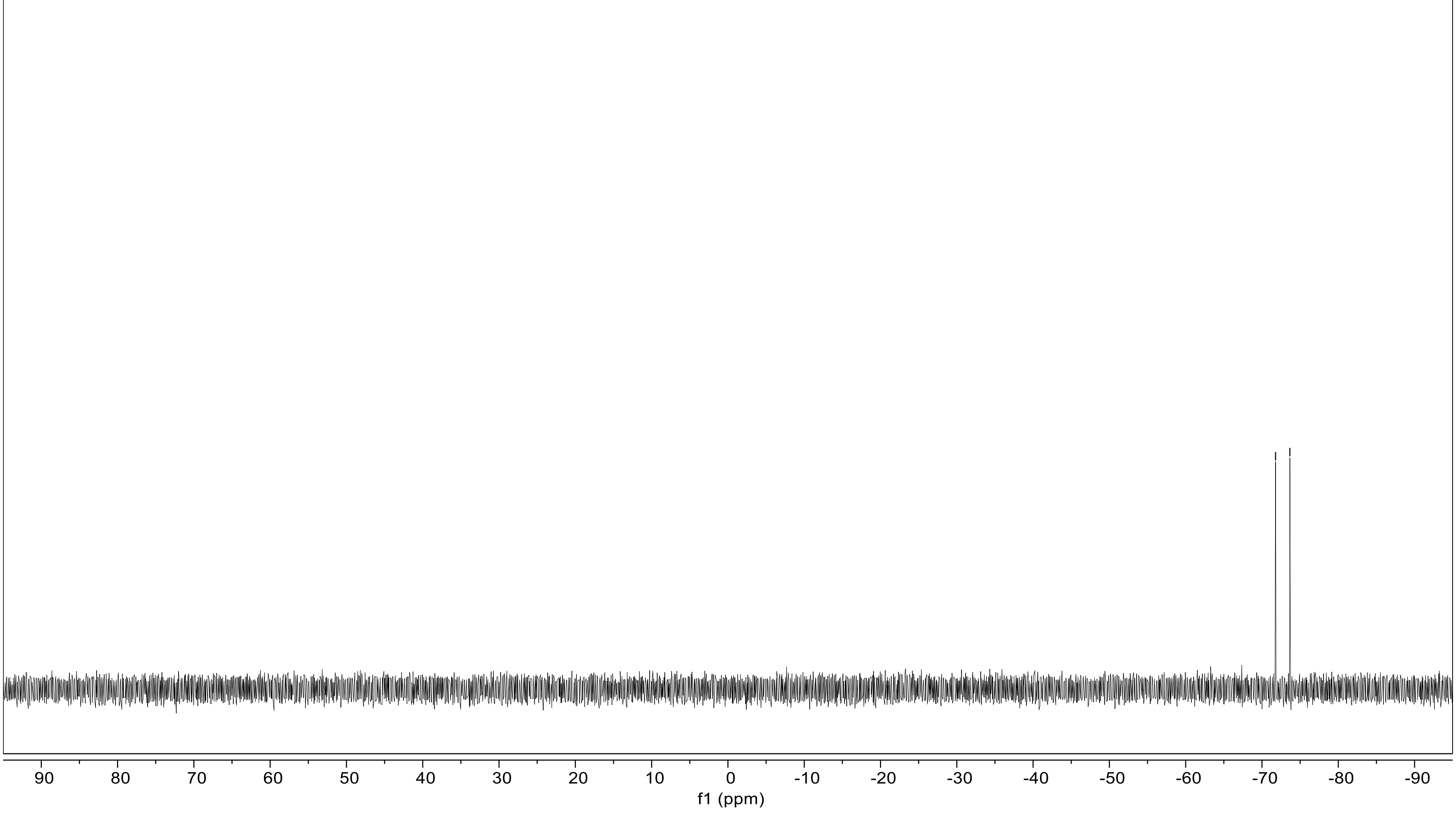

^{1}H NMR Spectrum of 5c (400 MHz, $CDCl_3$)

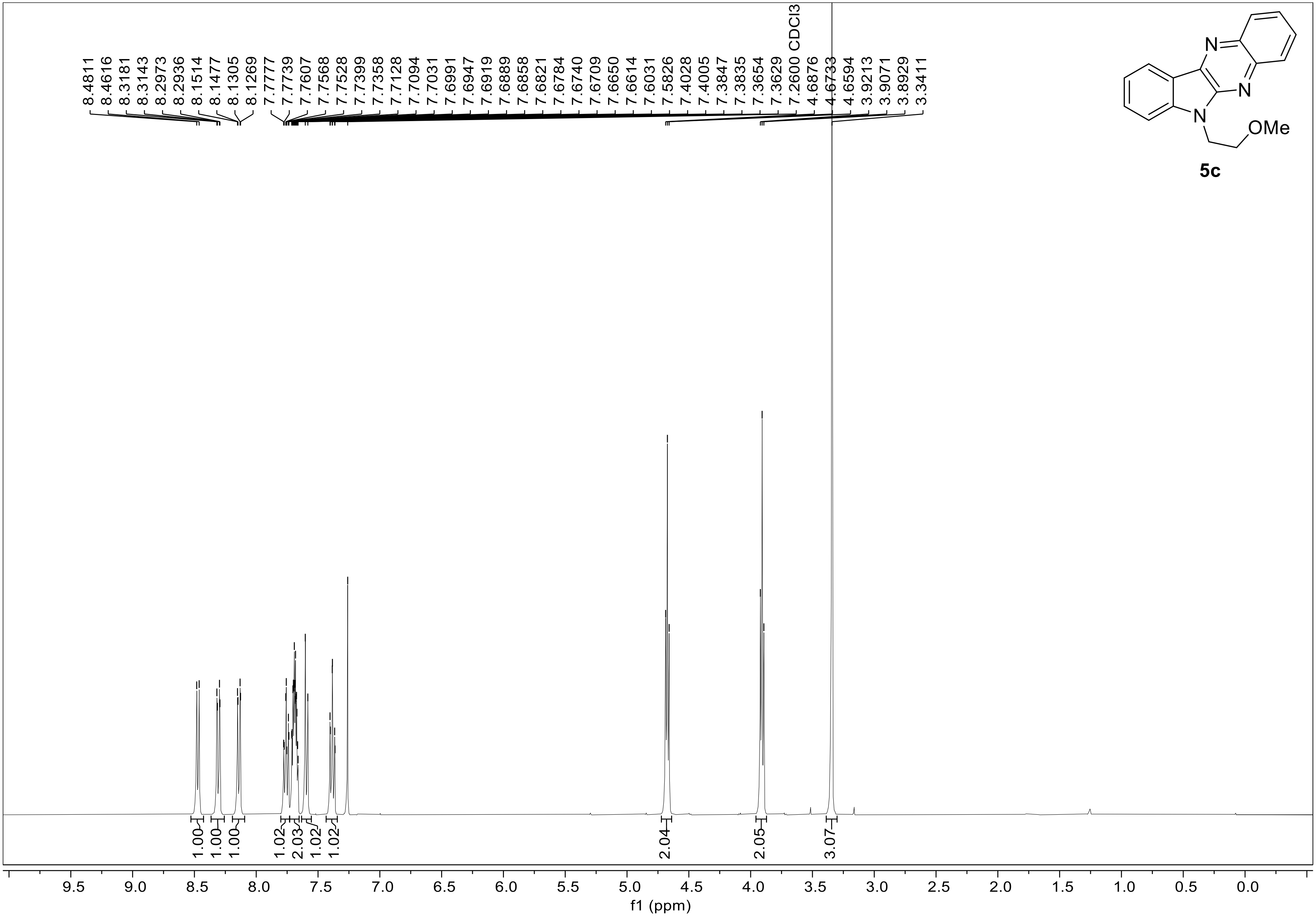

^{13}C NMR Spectrum of 5c (151 MHz, $CDCl_3$)

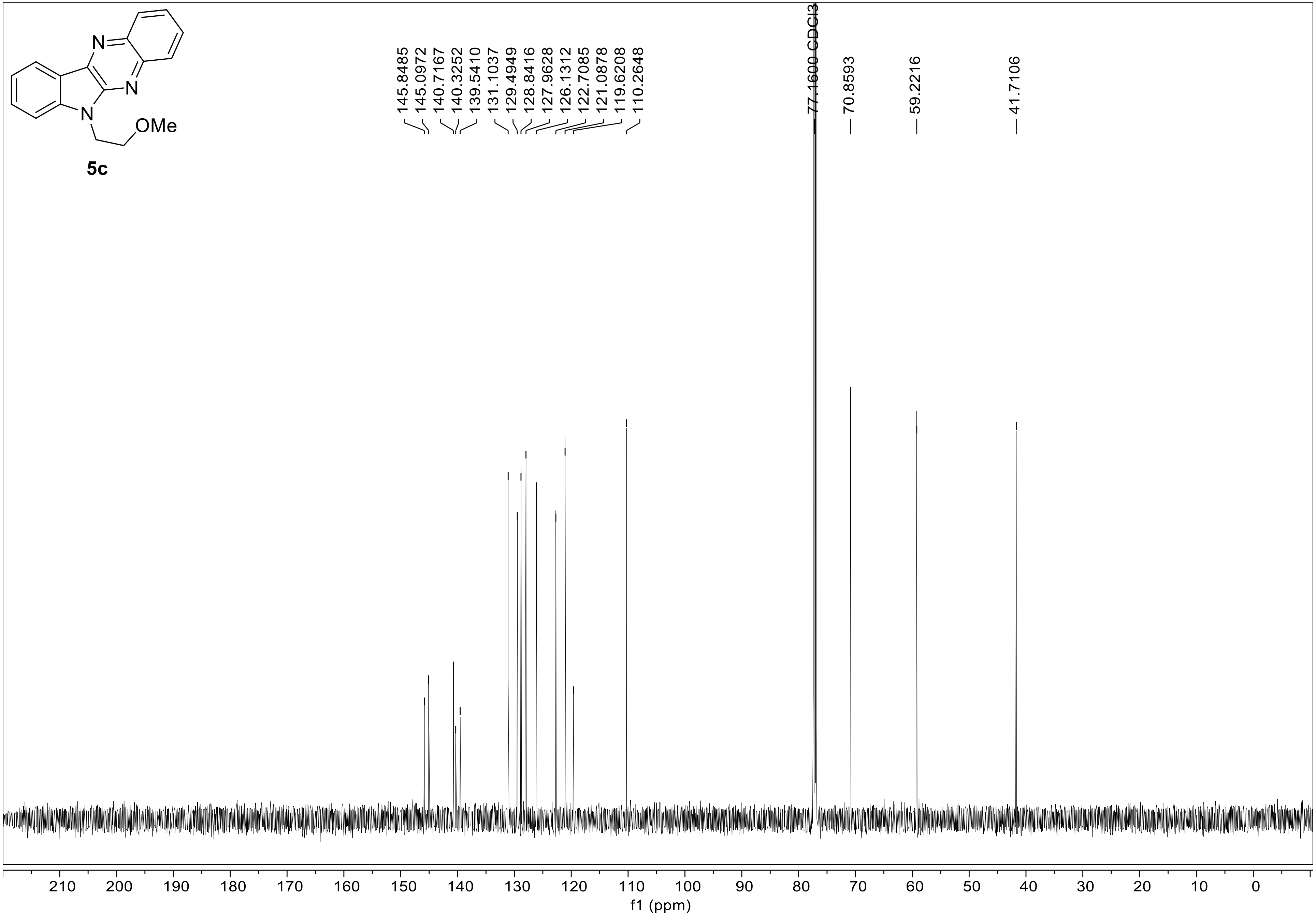

^{1}H NMR Spectrum of 5d (400 MHz, $CDCl_3$)

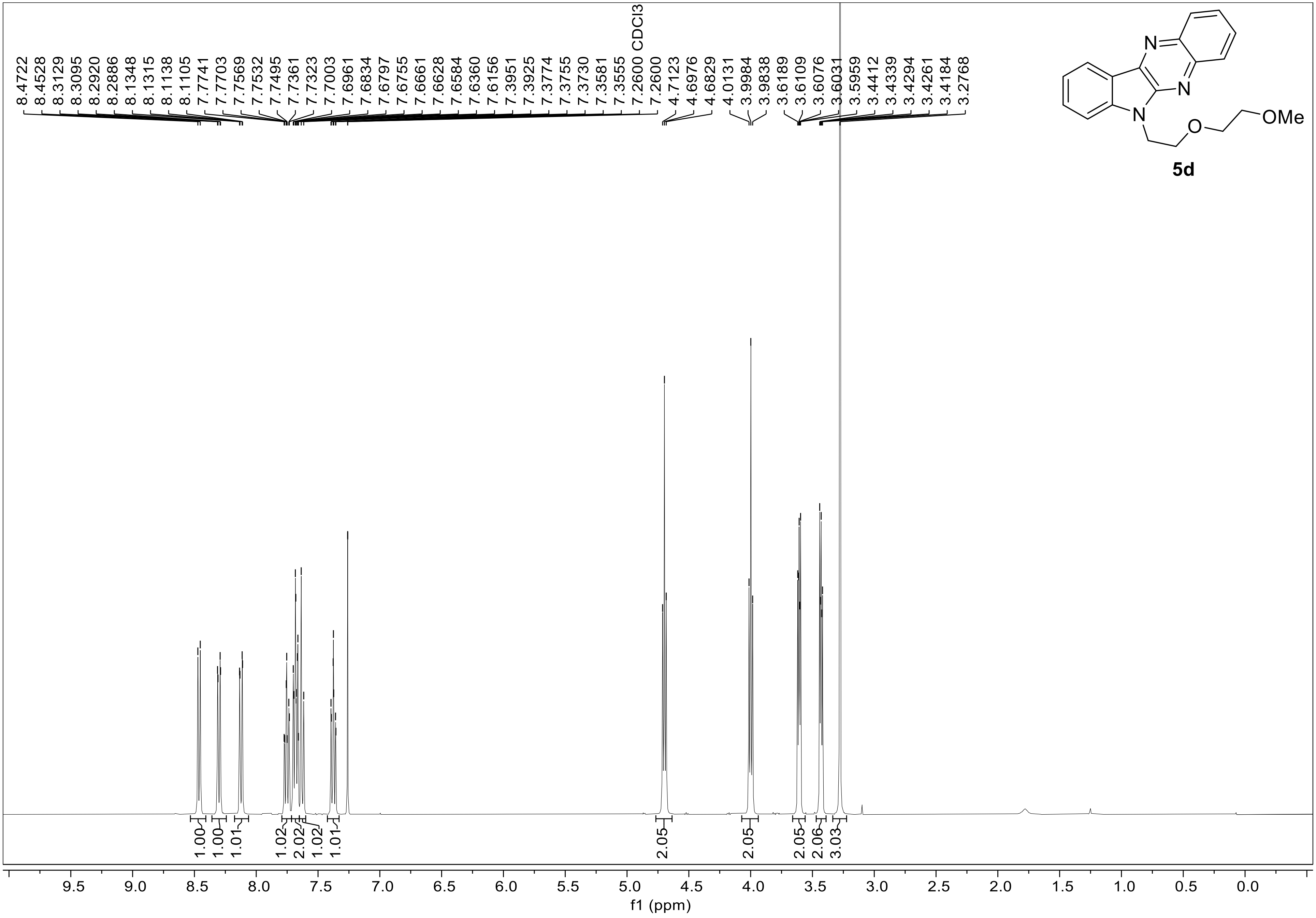

^{13}C NMR Spectrum of 5d (151 MHz, $CDCl_3$)

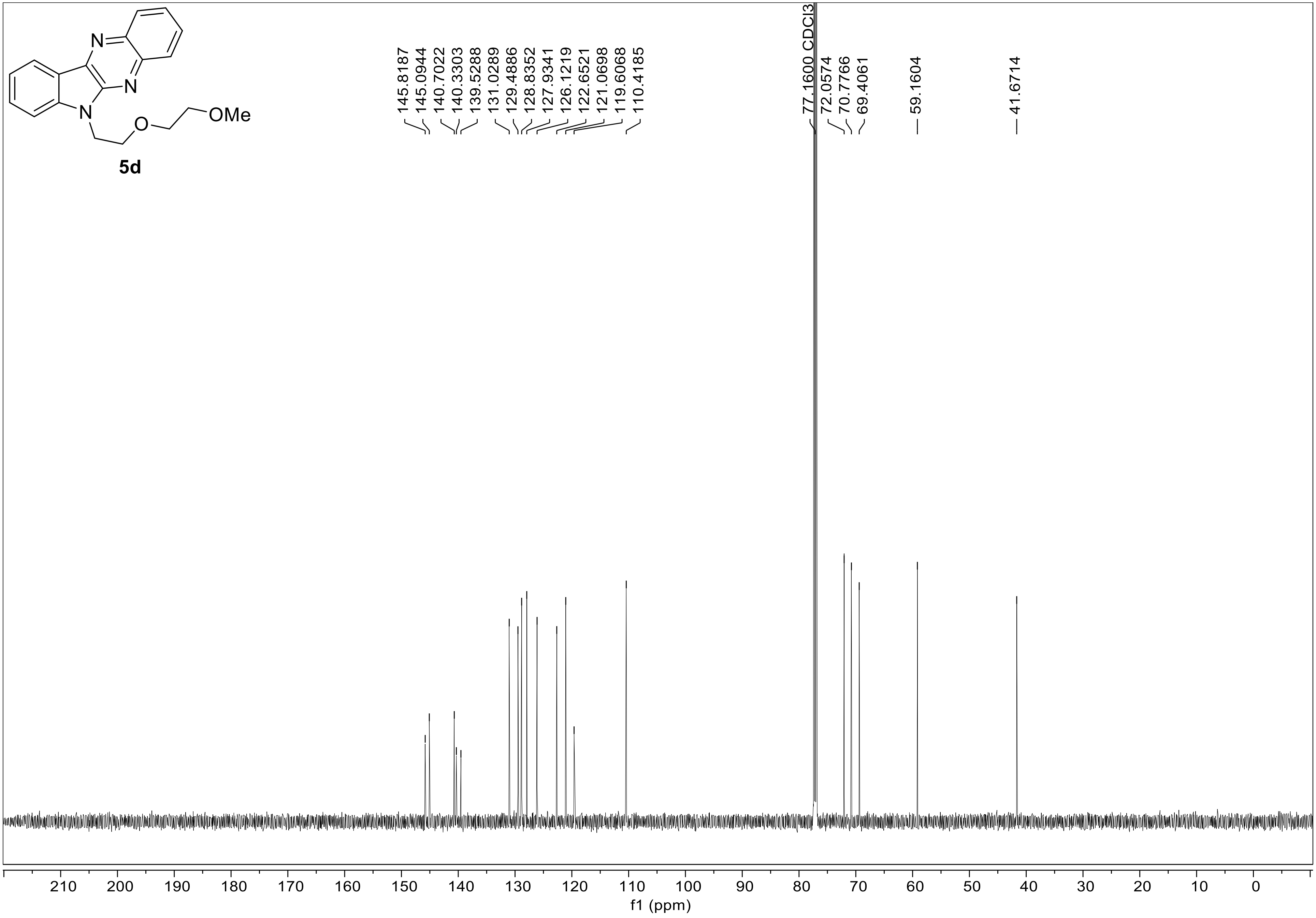

^{1}H NMR Spectrum of 5e (400 MHz, $CDCl_3$)

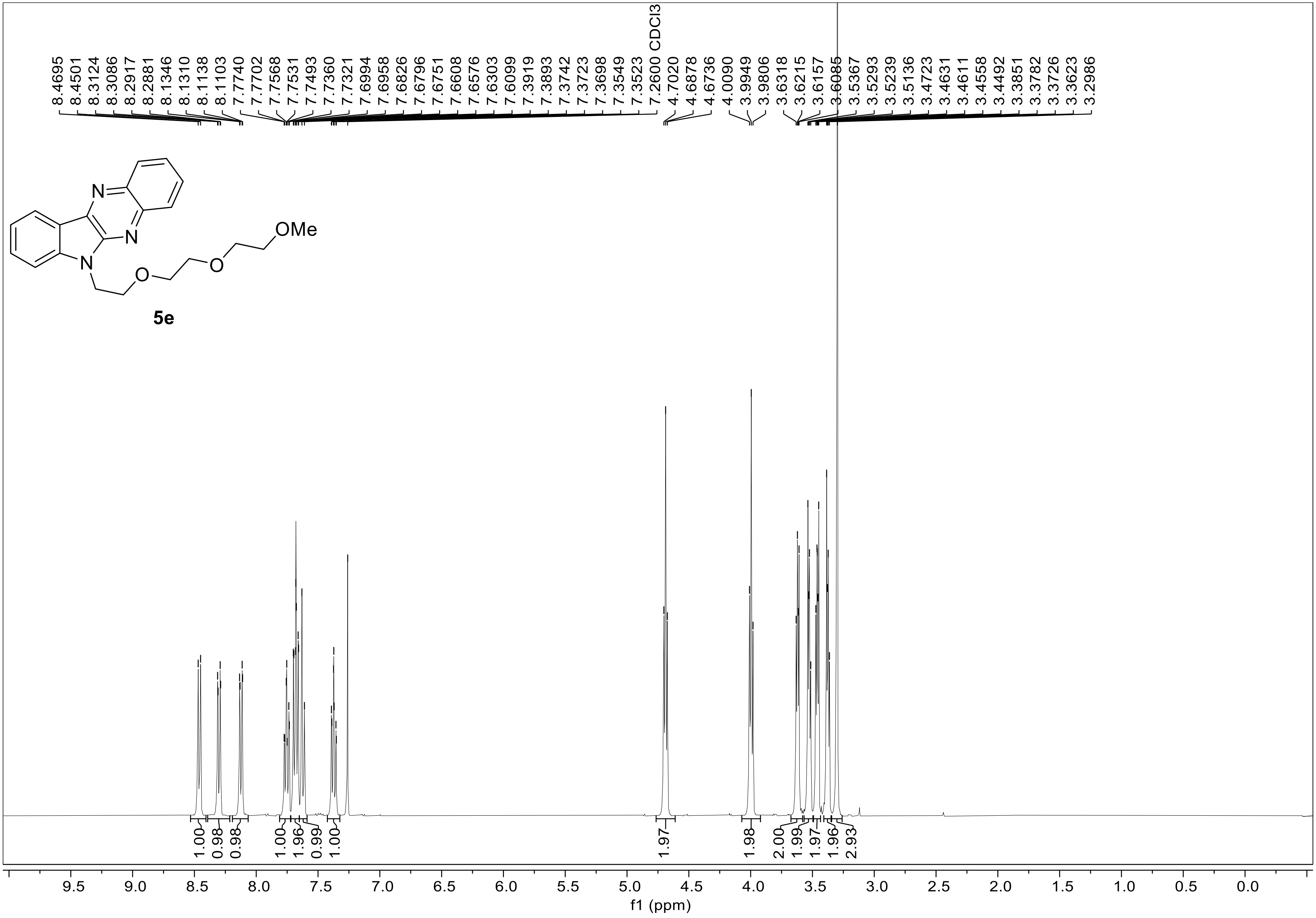

^{13}C NMR Spectrum of 5e (151 MHz, $CDCl_3$)

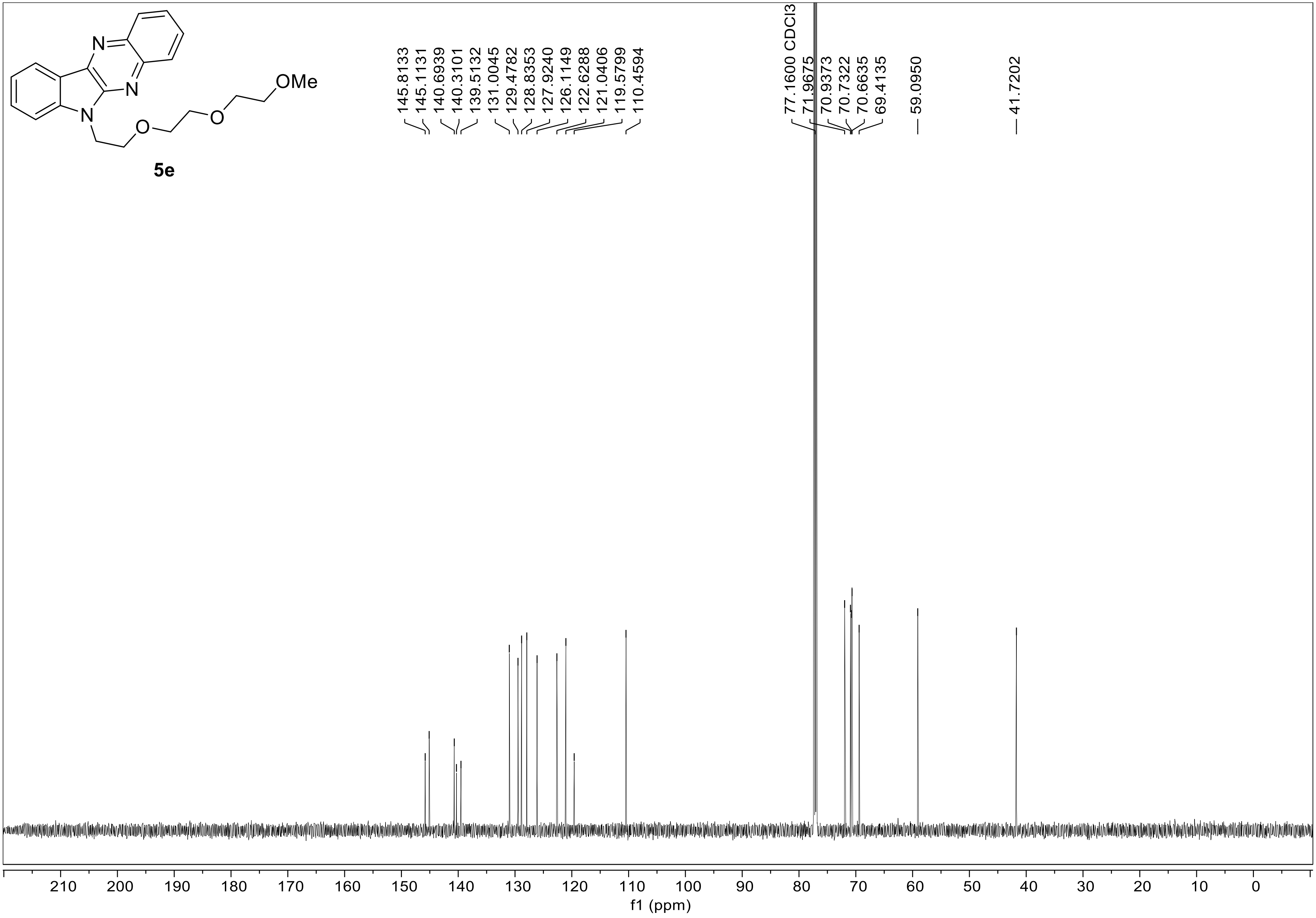

^{1}H NMR Spectrum of 5f (400 MHz, $CDCl_3$)

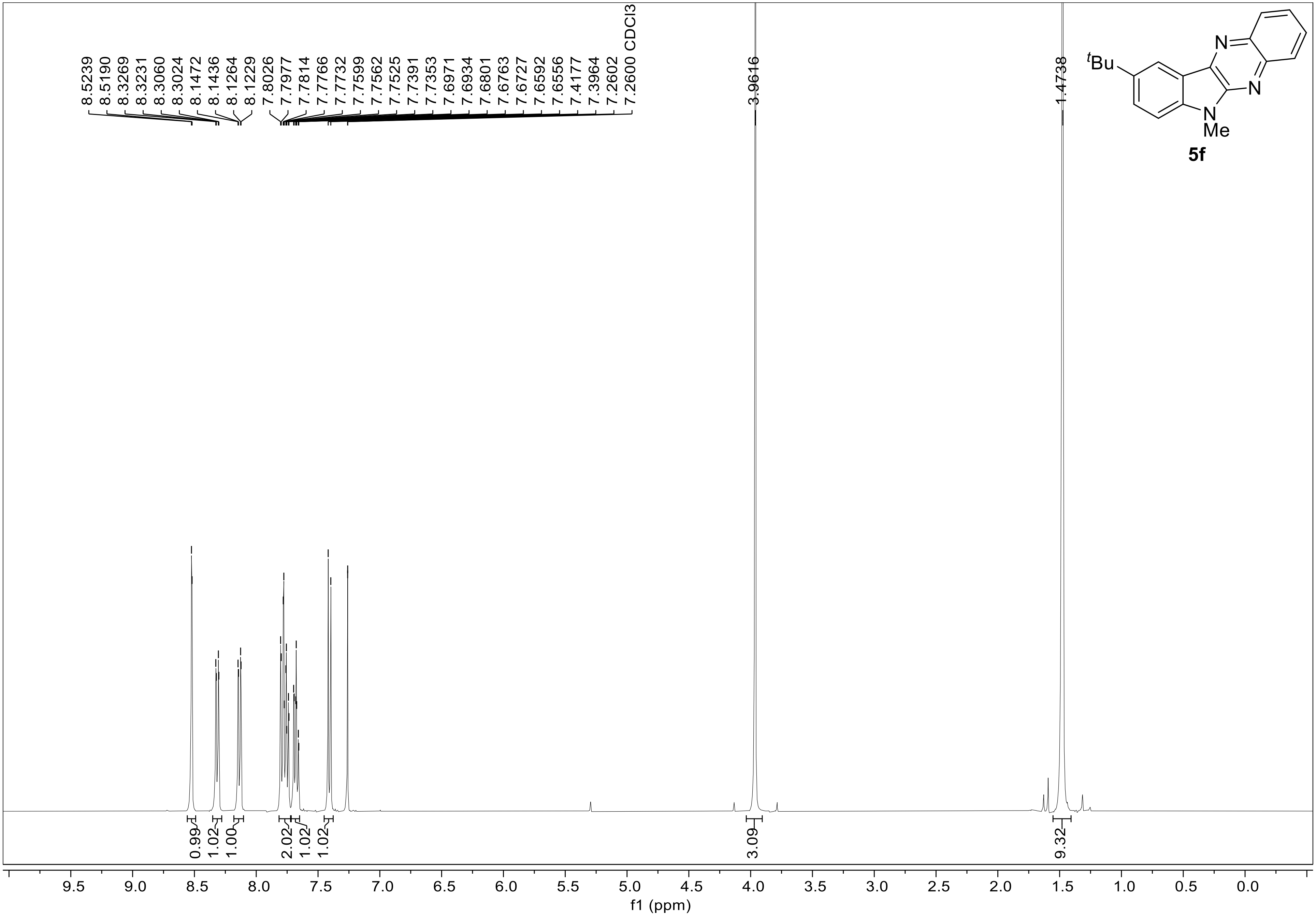

^{13}C NMR Spectrum of 5f (151 MHz, $CDCl_3$)

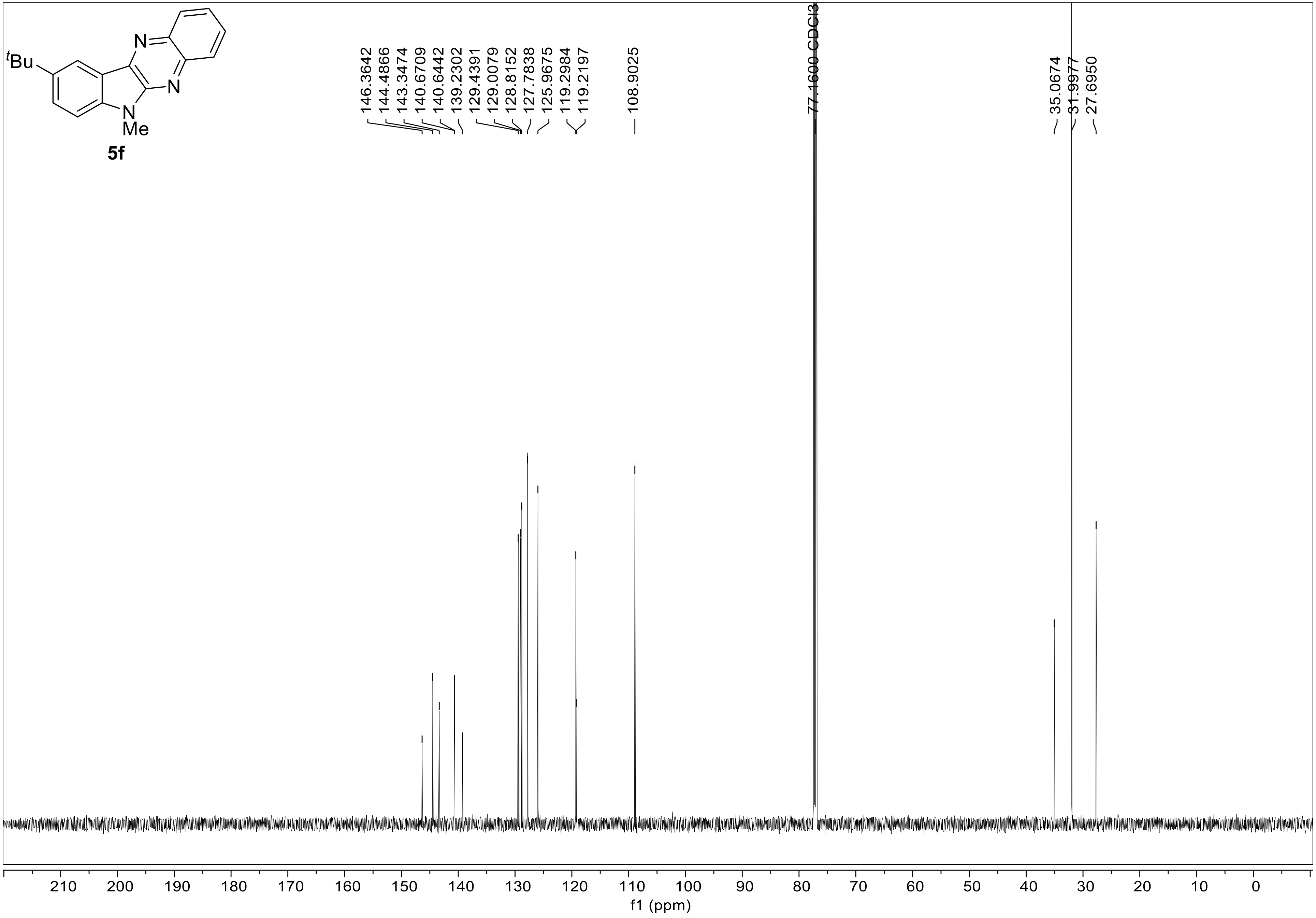

^{1}H NMR Spectrum of SI5 (400 MHz, $CDCl_3$)

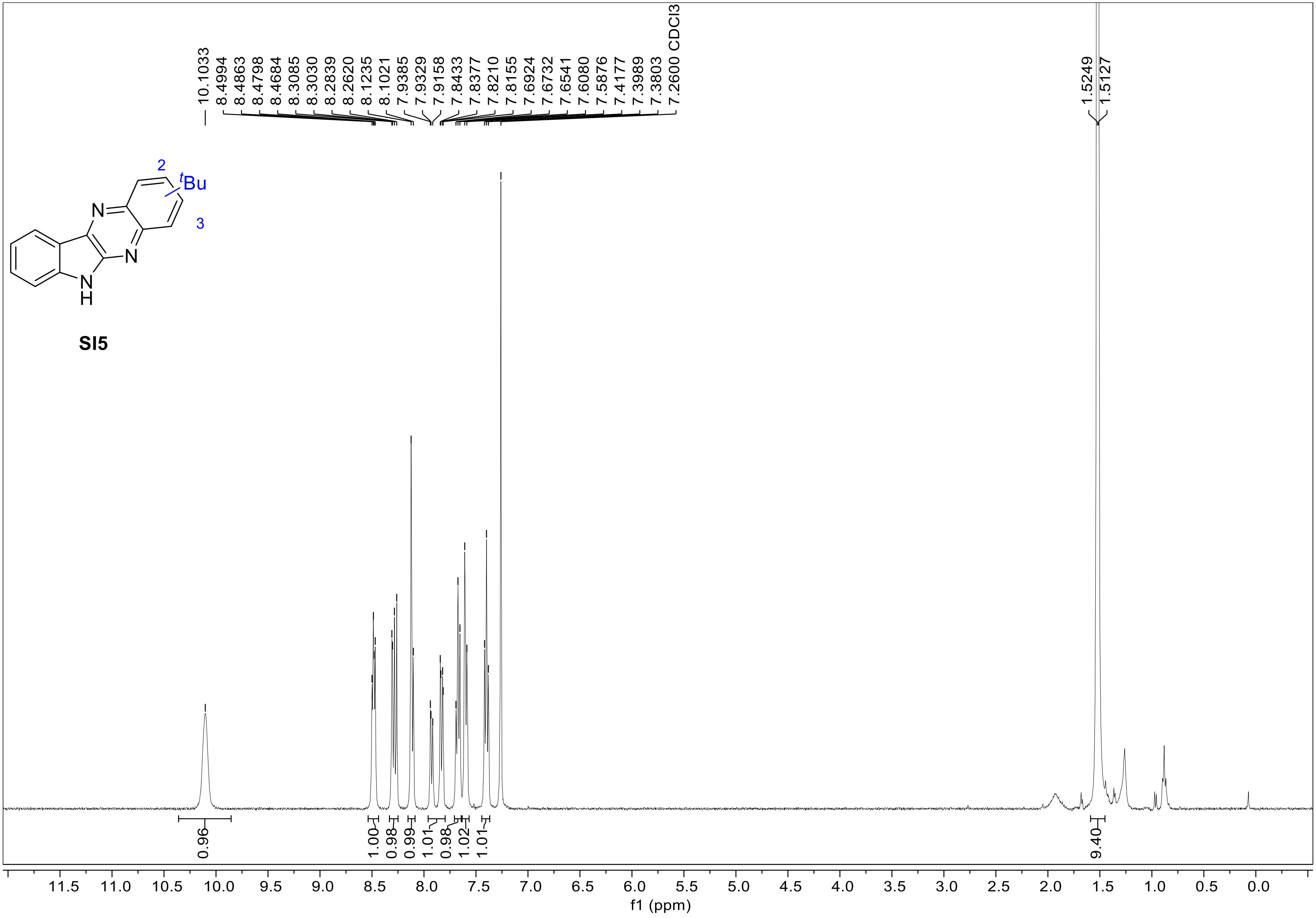

^{13}C NMR Spectrum of SI5 (151 MHz, $CDCl_3$)

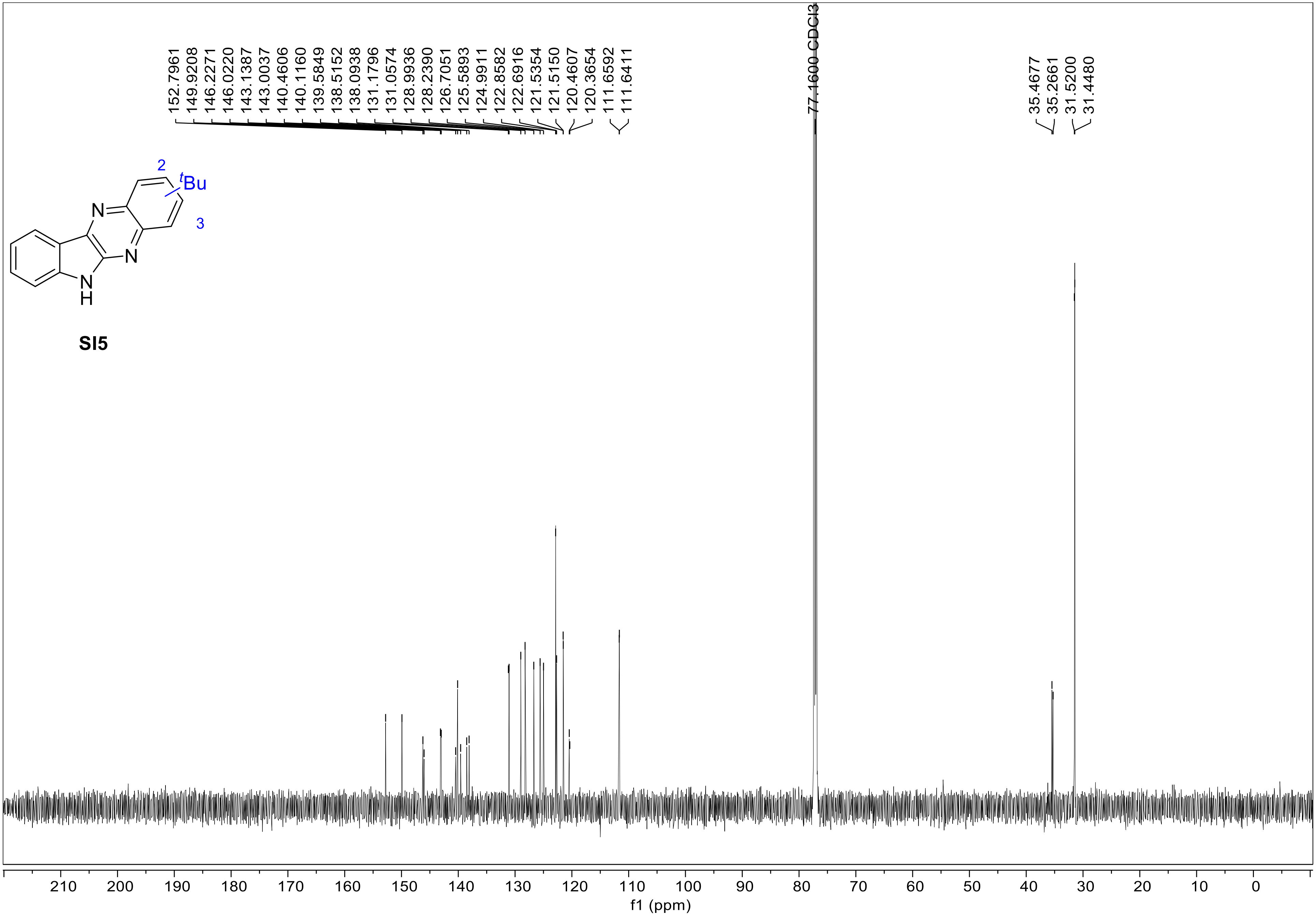

^{1}H NMR Spectrum of 5g (400 MHz, $CDCl_3$)

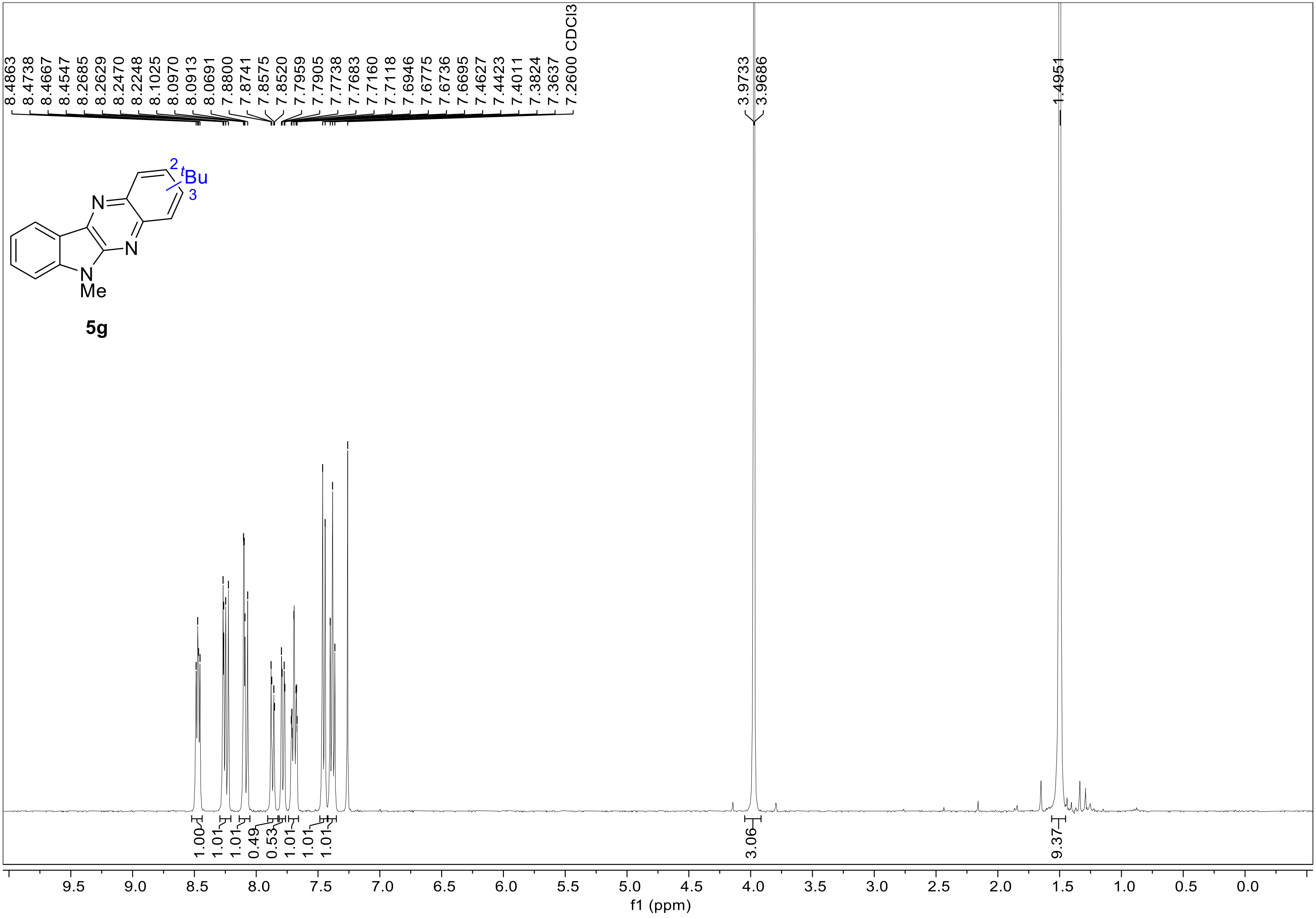

^{13}C NMR Spectrum of 5g (151 MHz, $CDCl_3$)

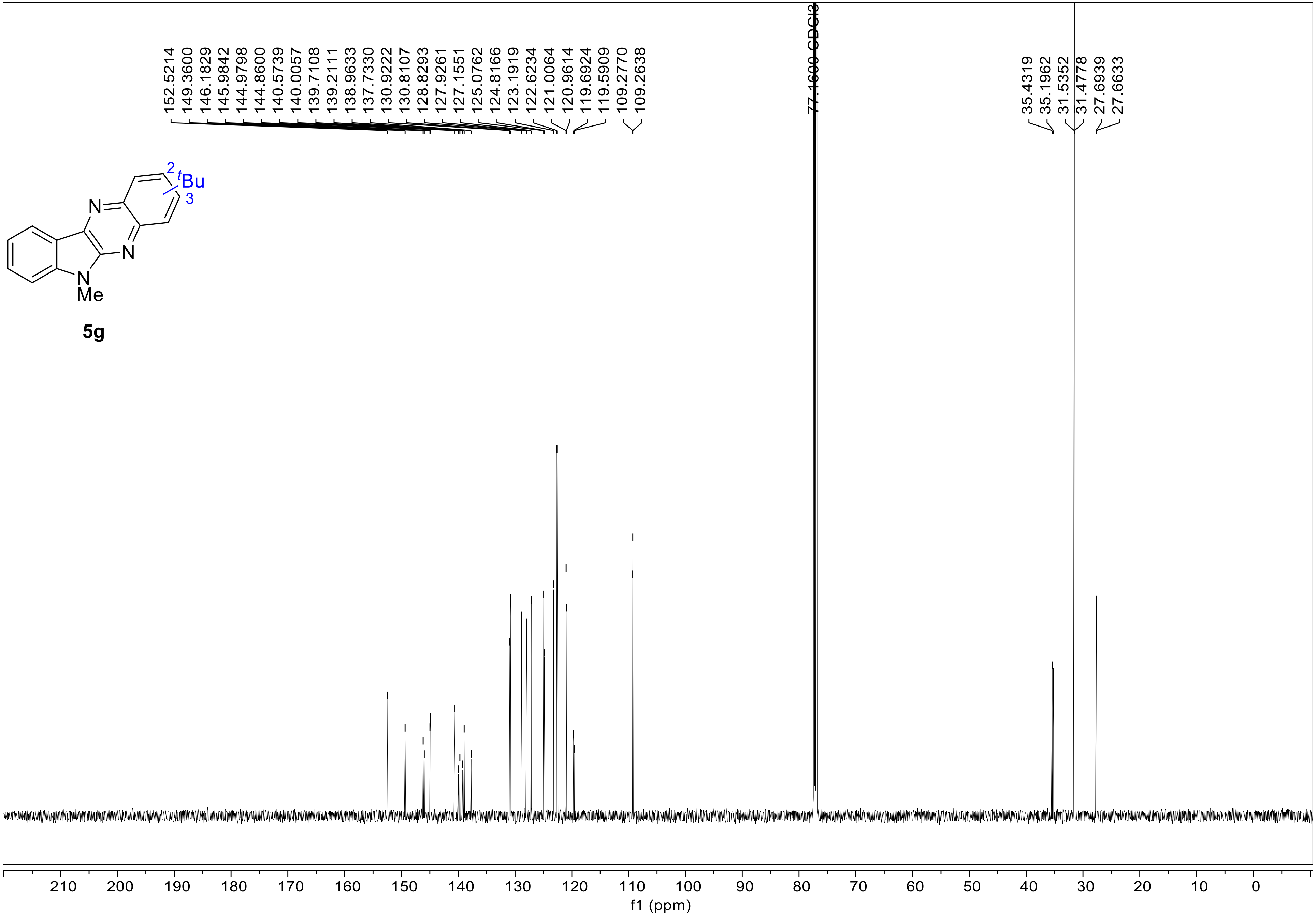

^{1}H NMR Spectrum of 5h (400 MHz, $CDCl_3$)

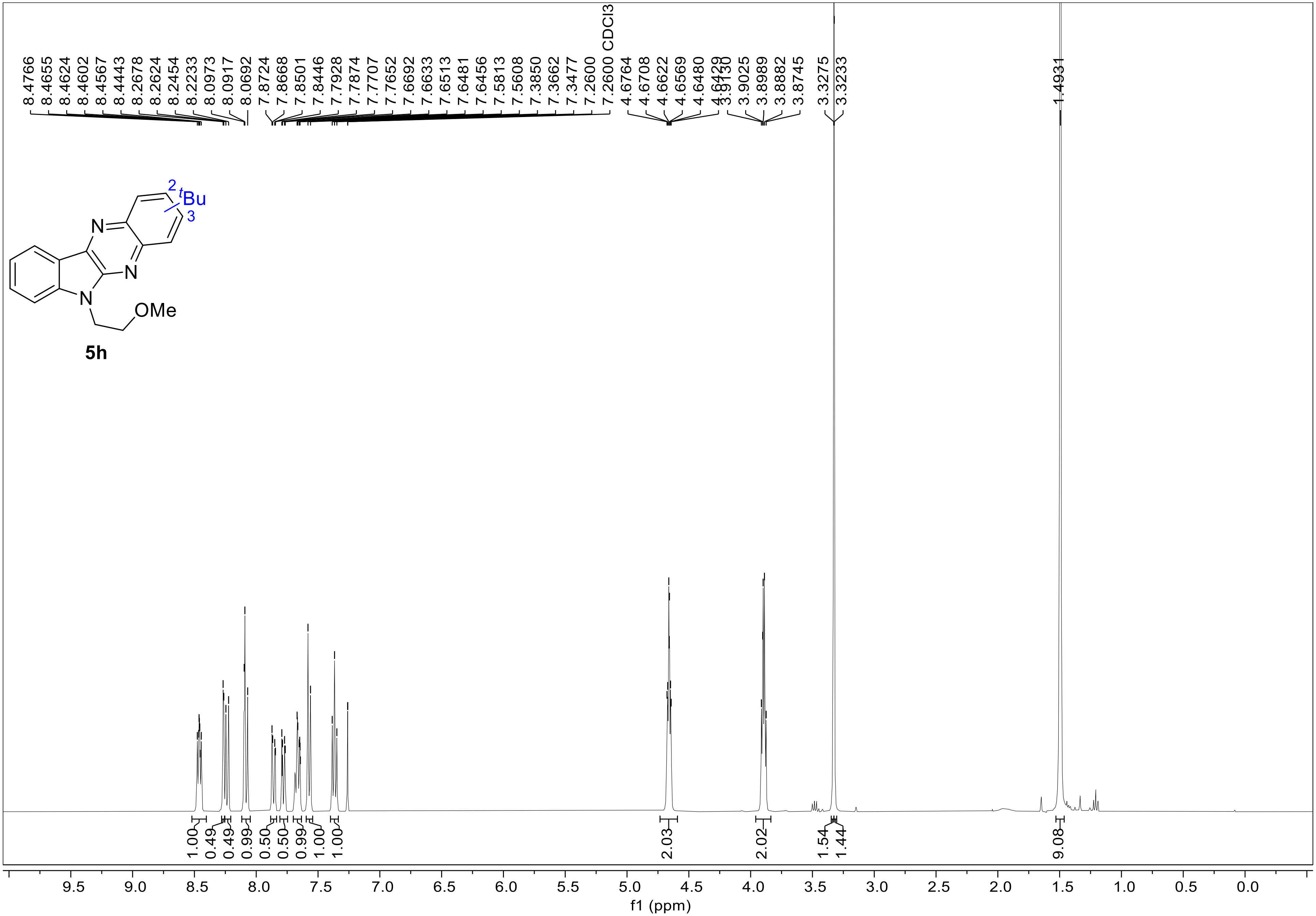

^{13}C NMR Spectrum of 5h (151 MHz, $CDCl_3$)

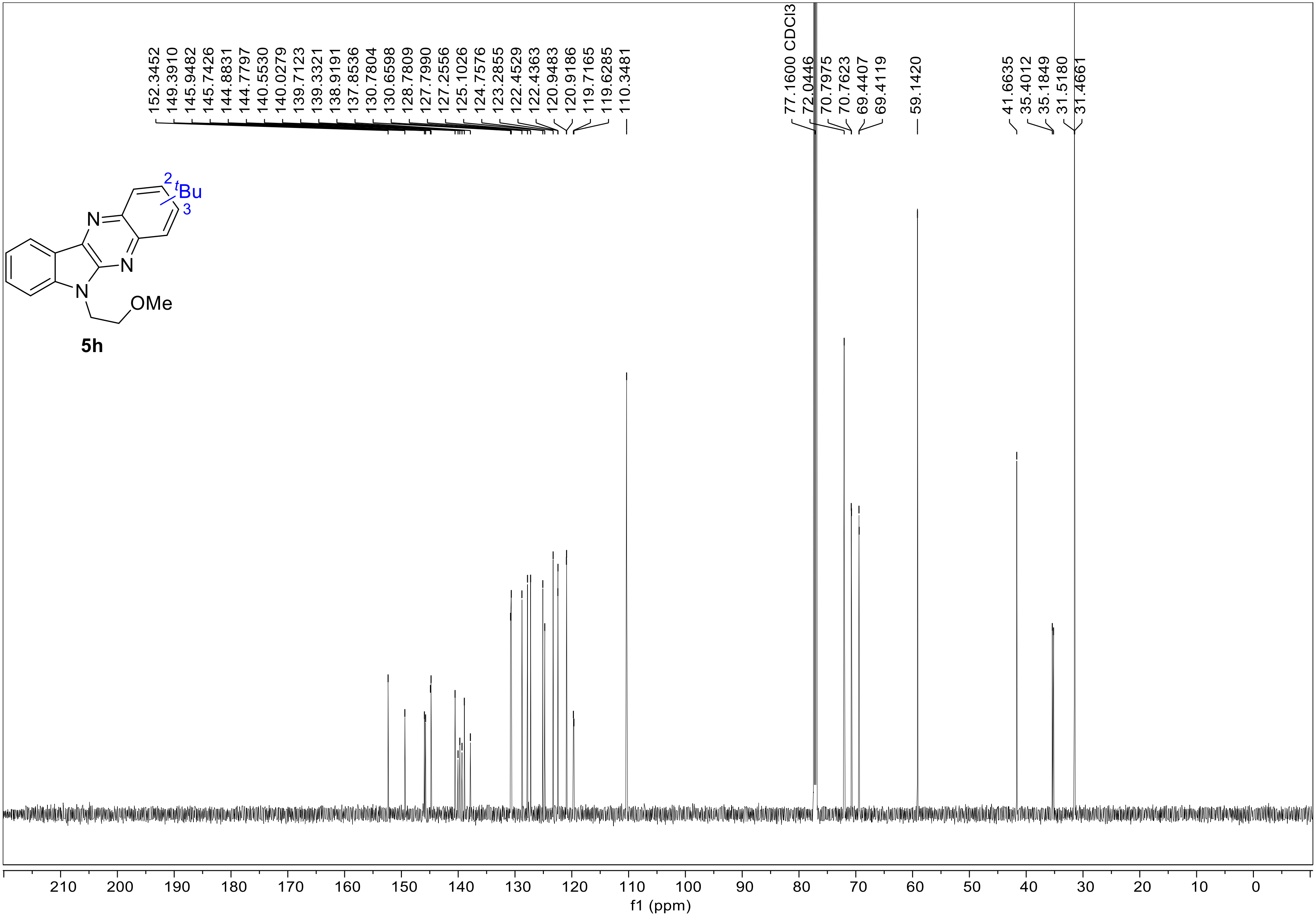

^{1}H NMR Spectrum of 5i (400 MHz, $CDCl_3$)

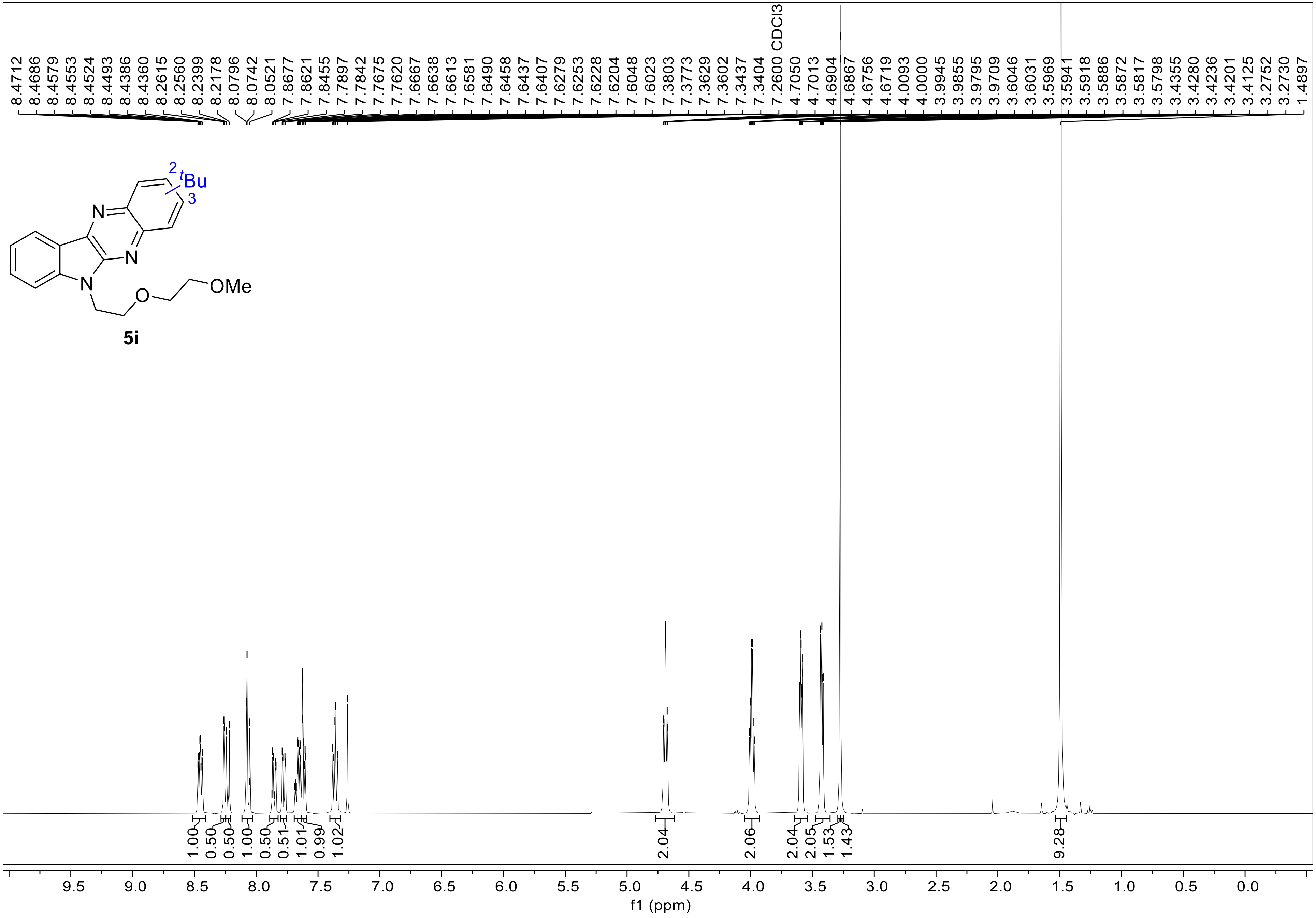

^{13}C NMR Spectrum of 5i (151 MHz, $CDCl_3$)

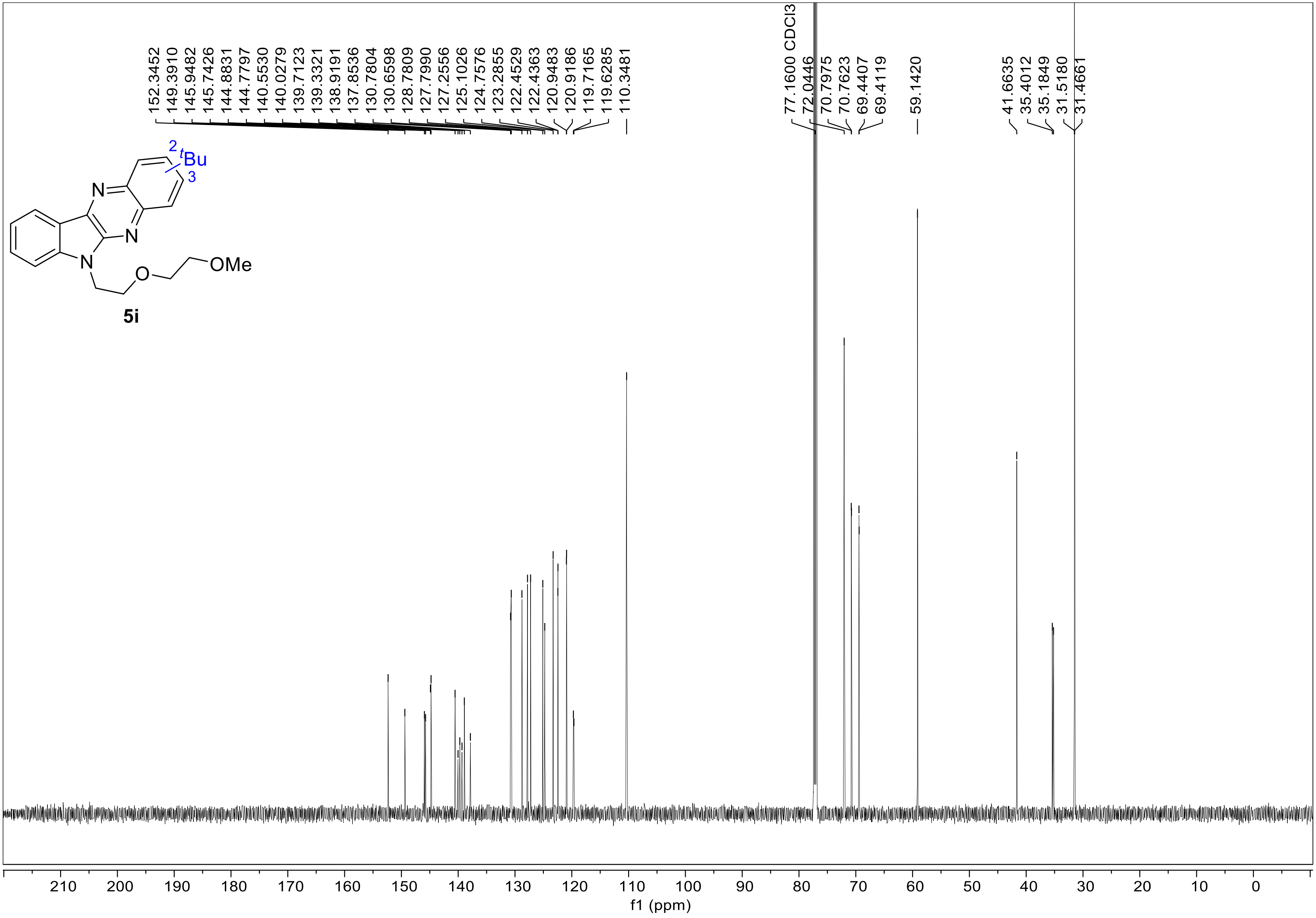

^{1}H NMR Spectrum of pre-cycling of 5h and 10 (600 MHz, CD_3CN)

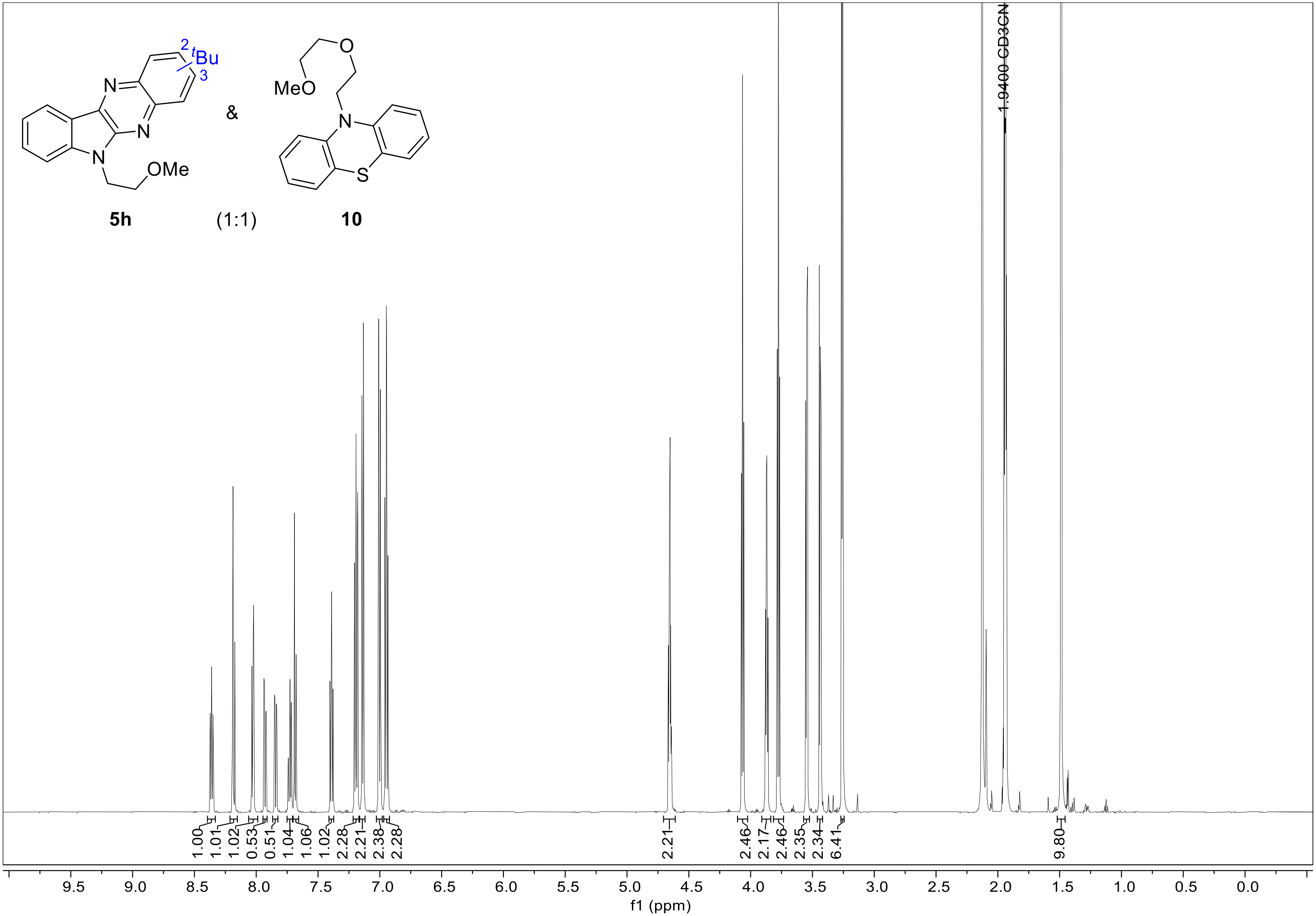

^{1}H NMR Spectrum of crude mixture of post-cycling of 100 mM 5h and 100 mM 10 (600 MHz, CD_3CN)

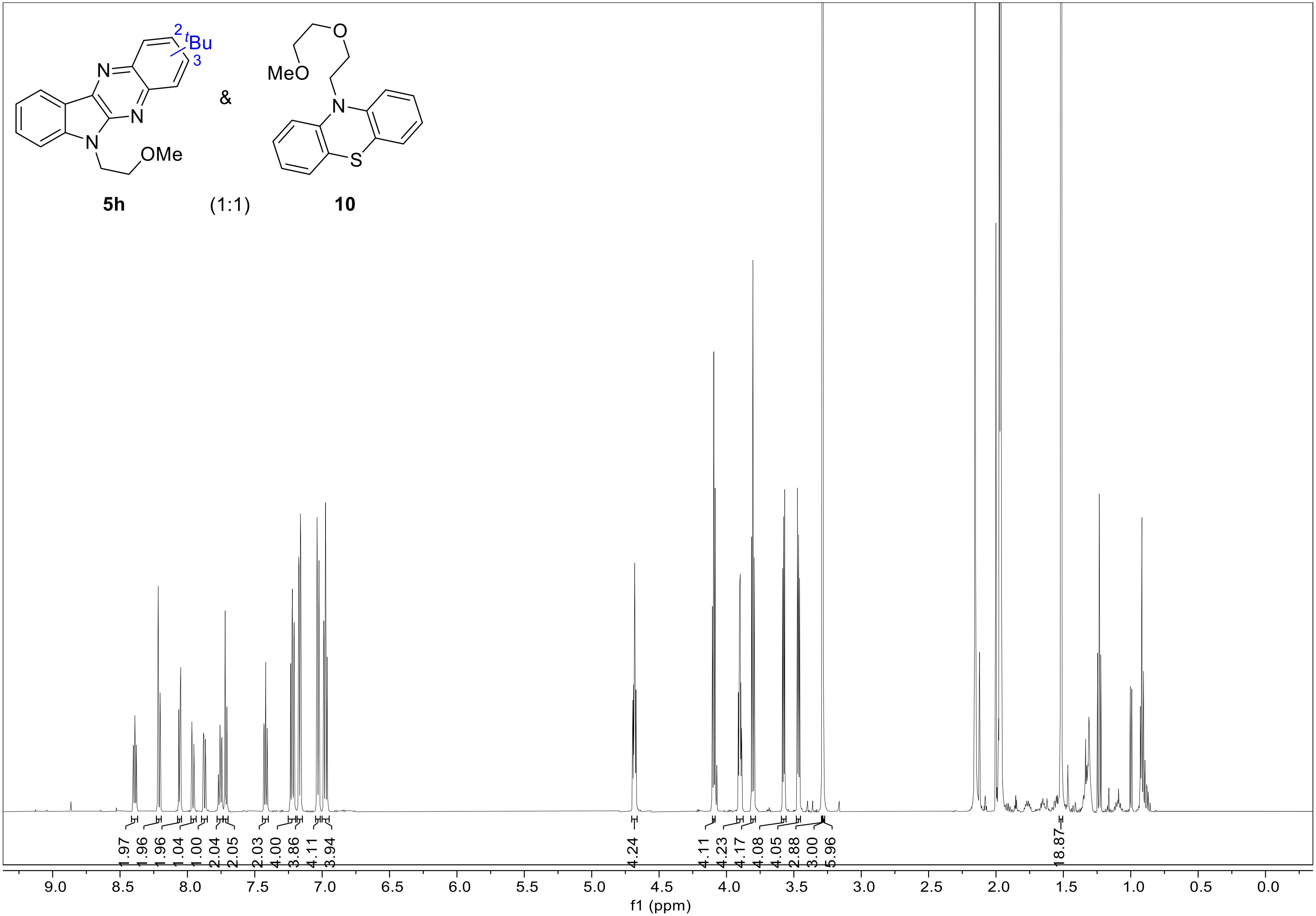

Comparison of ^{1}H NMR Spectrum of pre-cycling and post-cycling of 100 mM 5h and 100 mM 10 (600 MHz, CD_3CN)

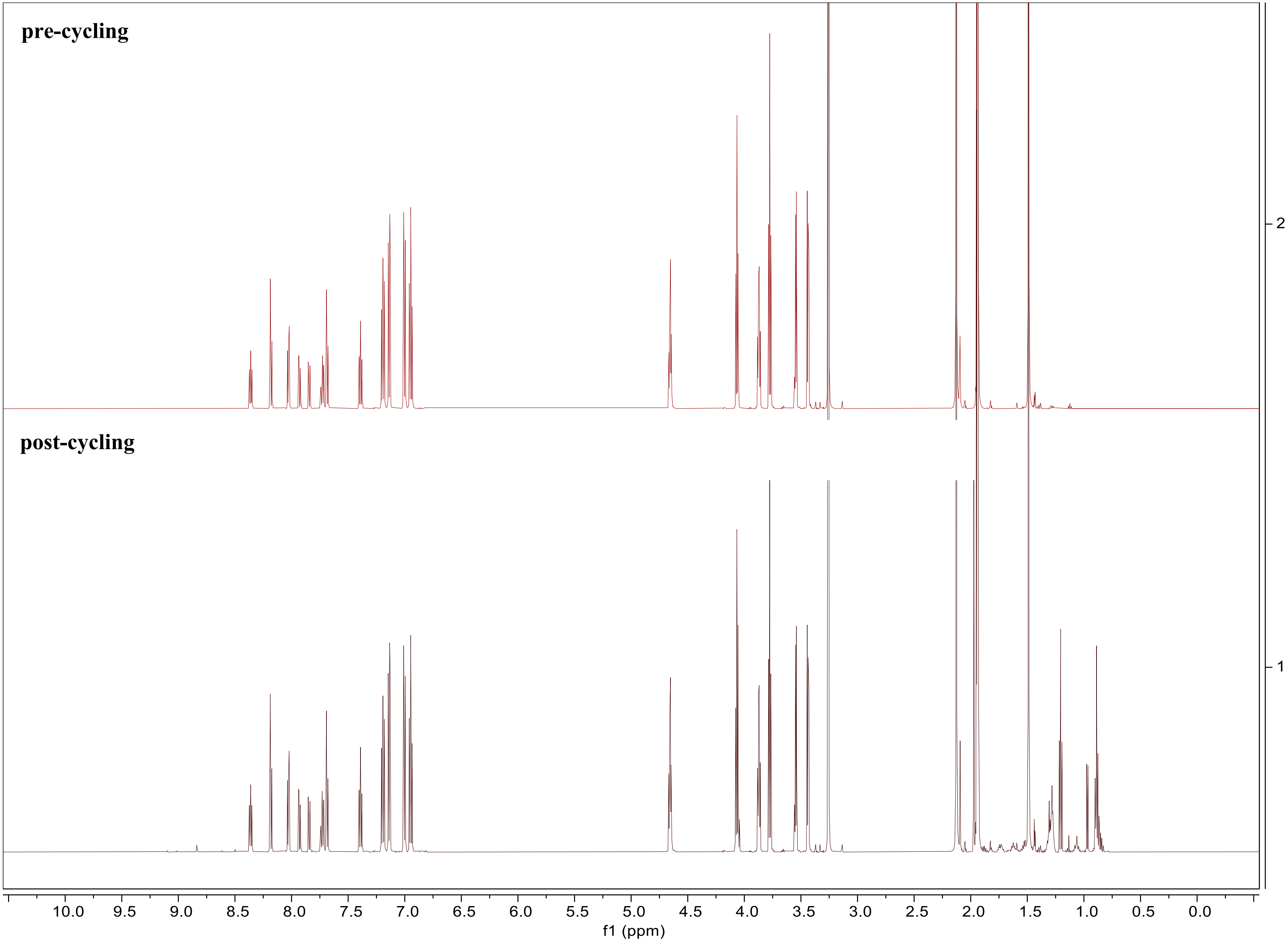

IV. Electrochemical Experiments Details, Materials, and Methods, Including Photographs of Experimental Setup.

General methods and materials: Acetonitrile (MeCN) (99.9%, extra dry over molecular sieves) was obtained from Thermo Scientific. Tetrabutylammonium hexafluorophosphate ($TBAPF_6$; >99%, for electrochemical analysis) was obtained from MilliporeSigma™, dried under high vacuum for 24 h at 80 °C and transferred to a N_2-filled glovebox for storage and use. All electrochemical experiments were performed in a N_2 filled glove box with an atmosphere <0.1 ppm oxygen and <0.1 ppm water. Electrolyte solutions were prepared in the glovebox by first drying the acetonitrile over freshly activated 3 Å molecular sieves for at least 24 h. Supporting electrolyte was then added and the solvent/electrolyte mixture was further dried for another 24 h before use. The resulting solvent/electrolyte mixtures were stored over the 3 Å molecular sieves in the glovebox. All potentials are reported relative to the ferrocene/ferrocenium couple (Fc/Fc^+), and this adjustment is made for each sample through the addition of a ferrocene reference at the end of each set of CV experiments.

Cyclic voltammetry: Cyclic voltammetry (CV) experiments were performed with a CH Instruments 760 Bipotentiostation with a three-electrode electrochemical cell. A glassy carbon electrode (BASi, 0.071 cm^2) was used as a working electrode, a Ag/Ag^+ electrode (10 mM $AgBF_4$ in 0.5 M $TBAPF_6$/MeCN) sealed with a Coralpor frit was used as a non-aqueous quasi-reference electrode (BASi), and platinum mesh was used as a counter electrode. The glassy carbon electrode was polished outside the glovebox using alumina (MicroPolish II, Buehler) in Milli-Q® water before being dried with acetone and brought into the glovebox. Unless otherwise indicated, all CV measurements were performed by dissolving the compound in stock 0.5 M $TBAPF_6$ in acetonitrile to give a concentration of 5 mM.

Linear sweep voltammetry: Linear sweep voltammetry (LSV) experiments were performed with a CH Instruments 760 Bipotentiostation with a three-electrode electrochemical cell. A rotating disk electrode (RDE) (glassy carbon, 5 mm in diameter, PINE) as the working electrode, a Ag/Ag^+ electrode (10 mM $AgBF_4$ in 0.5 M $TBAPF_6$/MeCN) sealed with a Coralpor frit was used as a non-aqueous quasi-reference electrode (BASi), and graphite electrode was used as a counter electrode.

H-cell cycling: Bulk charge/discharge measurements were carried out in a nitrogen-filled glovebox with a CH Instruments 760 Bipotentiostat in a custom H-cell (pictured below) with a fritted glass separator (P5). The working and counter electrodes were carbon (Duocel® RVC Foam, 100 PPI, 3% relative density). A Ag/Ag^+ quasi-reference electrode (described above) was used on the working side of the H-cell. The active compound was dissolved in 0.5 M $TBAPF_6$ in acetonitrile to give a redox-active material concentration of 5 mM. The working chamber of the H-cell was first loaded with 5 mL of the electrolyte/ROM solution while the counter chamber was loaded with 5 mL of only 0.5 M $TBAPF_6$ in acetonitrile. One charging event of the working chamber was completed at which point the solution was removed from the counter chamber and replaced with 5 mL of the electrolyte/ROM solution. A discharge event was then conducted followed by 201 more charge-discharge cycles. Charging and discharging were all conducted with a current of 5 mA and both chambers of the H-cell were continuously stirred with magnetic stir bars. 5 mL of 5 mM solution of electrolyte in 0.5 M $TBAPF_6$/MeCN is placed in the working side with 5 mL of 0.5 M $TBAPF_6$/MeCN in the counter side. Electrolyte is reduced/oxidized at a constant rate (−5/+5 mA) to a voltage limit of 350 mV lower/higher than the $E_{1/2}$ (determined with CV at a scan rate of 100 mV/s) of electrolyte to maximize the state-of-charge, and then the current was reversed to regenerate neutral electrolyte. This cycle was repeated iteratively to evaluate the cycling stability of electrolyte. Results of H-cell bulk electrolysis are displayed here in graphs of normalized discharge capacity (*normalized relative to theoretical capacity*) vs cycle number for 202 charge-discharge cycles. Each data point in these figures represents one cycle. ***The capacity fade is based on the maximum discharge capacity unless otherwise indicated.***

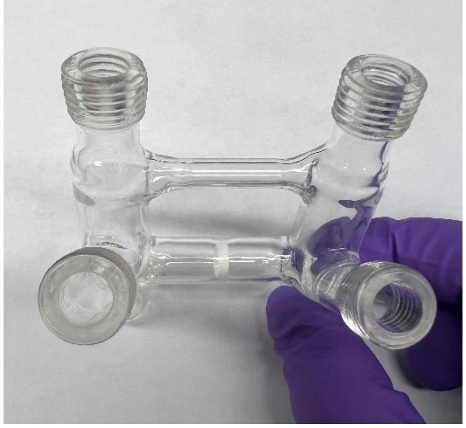

Picture of custom H-cell

Flow Cell Cycling: Data from flow cell cycling was collected on a BioLogic VSP potentiostat (operated in a galvanostatic mode) in a nitrogen-filled glovebox. The flow cell design used in these experiments was previously described in the literature by Brushett and coworkers (pictured below).[1]
The cell utilizes a zero-gap design that features graphite charge collectors built with interdigitated flow fields. Two layers of carbon felt (Sigracet® 29AA) with approximately 20% compression and with an active cross-sectional area of 2.55 cm^2 were utilized as electrodes on each side of the flow cell and held in place with ePTFA gaskets. The two sides of the flow cell were separated by a Daramic® A175 porous separator. All components of the flow cell were dried in an oven (~70 °C) overnight, assembled outside of the glovebox, and immediately brought into the glovebox through an antechamber via a 3-hour evacuation/nitrogen backfill process. The catholyte and anolyte side of the cell were loaded with 7.0 mL of a solution containing mixed 50 mM **5h** and 50 mM **10** or 7.5 mL of a solution containing mixed 100 mM **5h** and 100 mM **10**. The assembled flow cell was allowed to equilibrate in the glovebox for 24 hours prior to use. The continuous flow was provided by a Cole-Parmer Masterflex® pump featuring a two-channel rotor through a combination of Masterflex compressible pump tubing and PFA flexible tubing. Flow rates were 10 mL/min with charge and discharge rates of 10 mA/cm^2 and 20 mL/min with charge and discharge rates of 20 mA/cm^2. The upper and lower voltage cutoffs were 2.68 V and 1.98 V for charge and discharge rates of 10 mA/cm^2. The upper and lower voltage cutoffs were 2.73 V and 1.70 V for charge and discharge rates of 20 mA/cm^2. An equilibration period of 1 hour was utilized before active charging and discharging, during which the working solutions flowed through the cell. ***The capacity fade is based on the maximum discharge capacity unless otherwise indicated.***

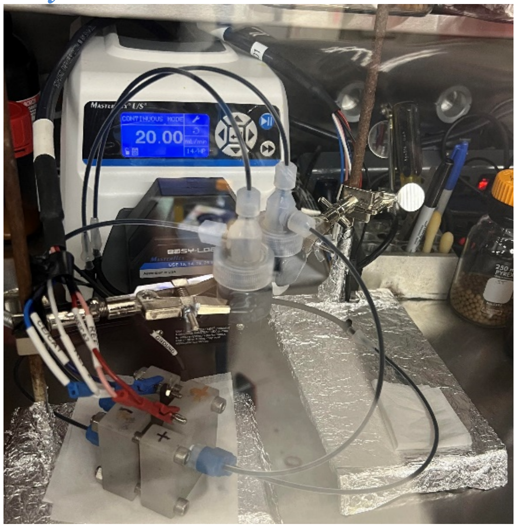

Picture of flow cell during operation

V. Electrochemical Characterization Data

6-methyl-*6H*-indolo[2,3-*b*]quinoxaline (5a):

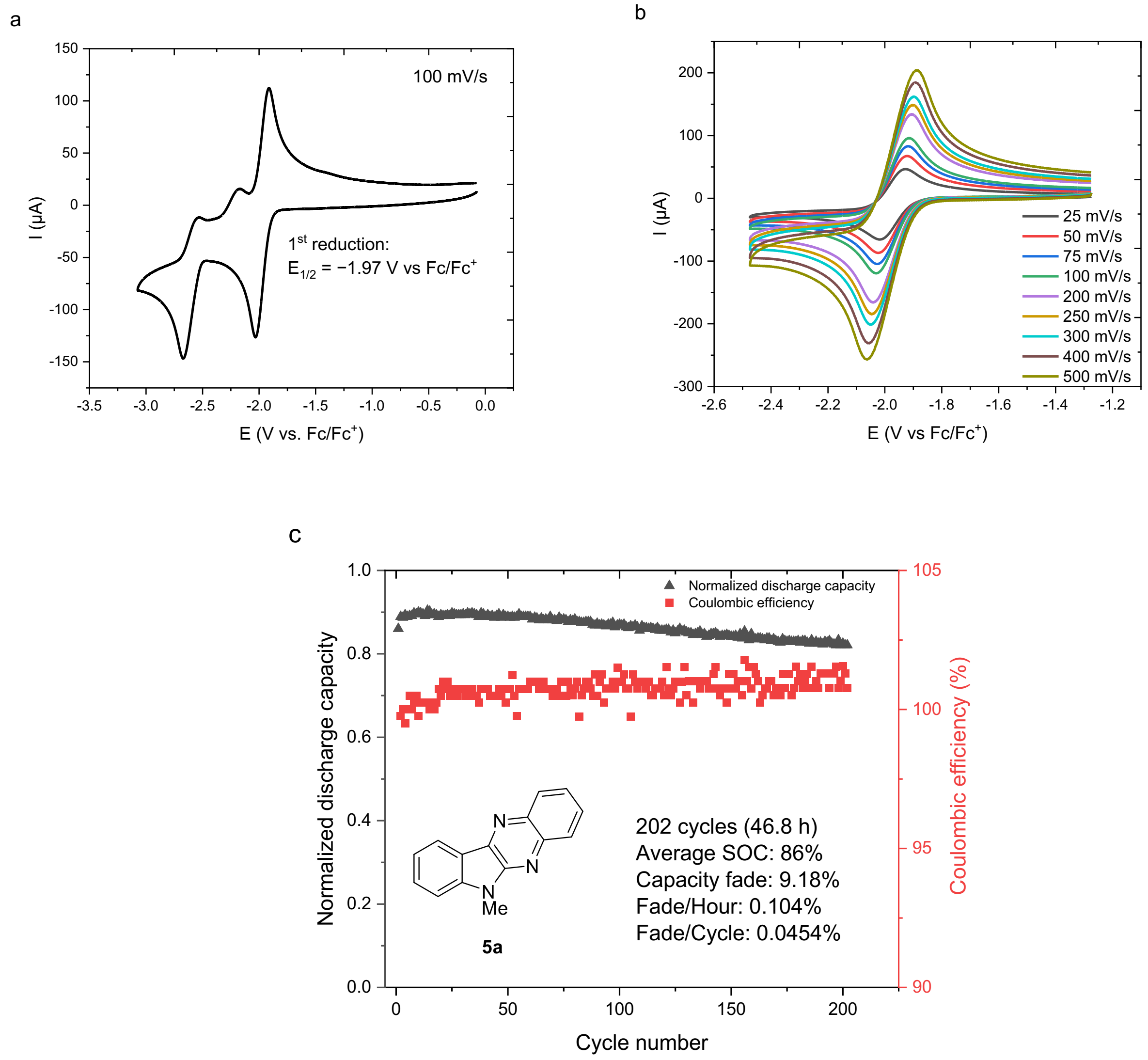

Figure SI1: (a). CV of **5a** (5 mM) in 0.5 M $TBAPF_6$/MeCN solution with glassy carbon working electrode at a scan rate of 100 mV/s. (b) Scan-rate-dependent CVs of **5a**. (c) Normalized discharge capacity and Coulombic efficiency versus cycle number of **5a**.

Quinoxaline (8):

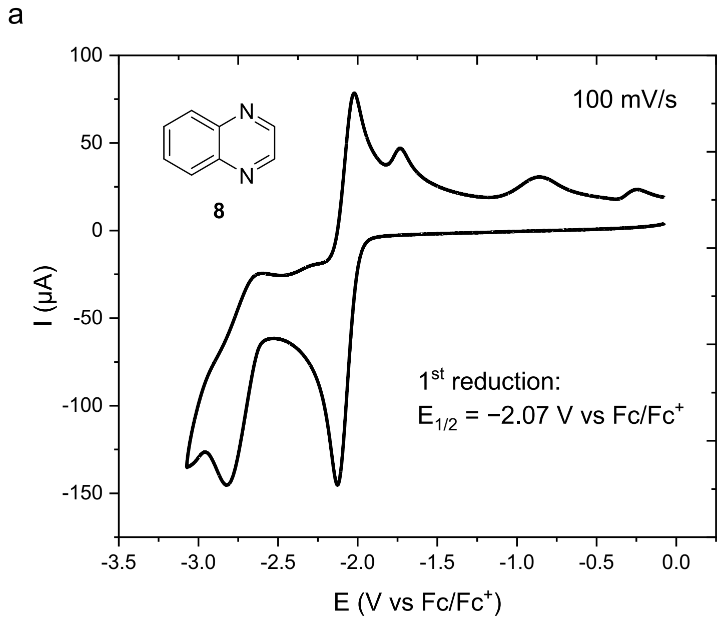

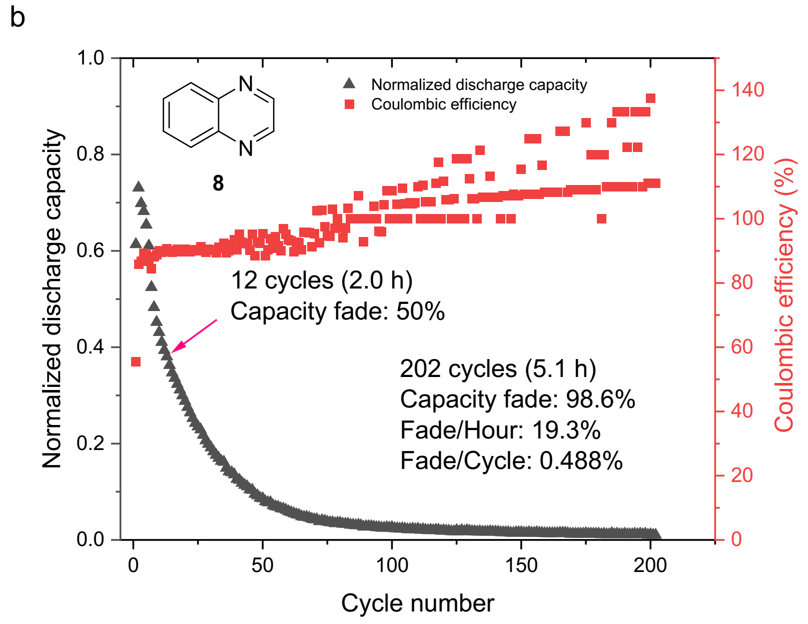

Figure SI2: (a). CV of **8** (5 mM) in 0.5 M $TBAPF_6$/MeCN solution with glassy carbon working electrode at a scan rate of 100 mV/s. (b) Normalized discharge capacity and Coulombic efficiency versus cycle number of **8**.

Phenazine (9):

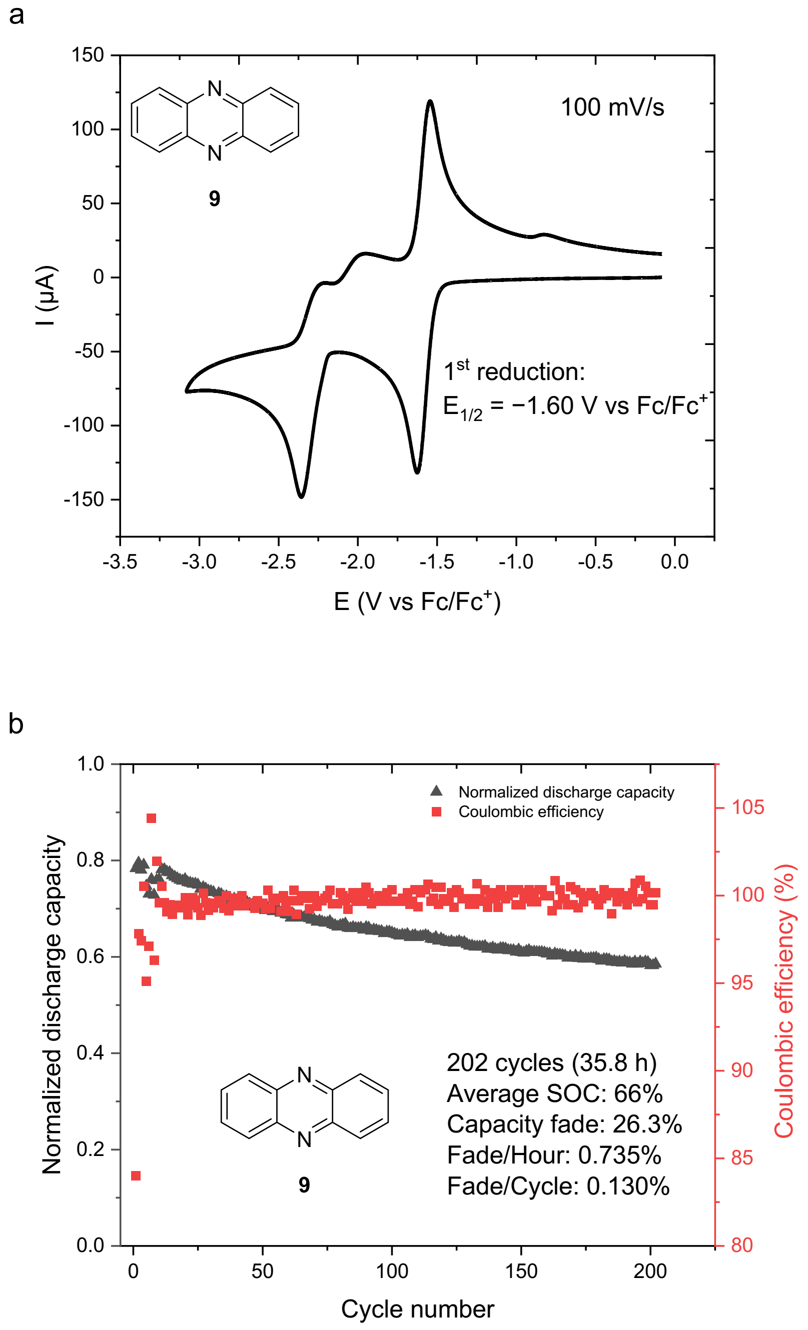

Figure SI3: (a). CV of **9** (5 mM) in 0.5 M $TBAPF_6$/MeCN solution with glassy carbon working electrode at a scan rate of 100 mV/s. (b) Normalized discharge capacity and Coulombic efficiency versus cycle number of **9**.

3-(*6H*-indolo[2,3-*b*]quinoxalin-6-yl)-*N,N,N*-trimethylpropan-1-aminium hexafluorophosphate(V) (5b):

a

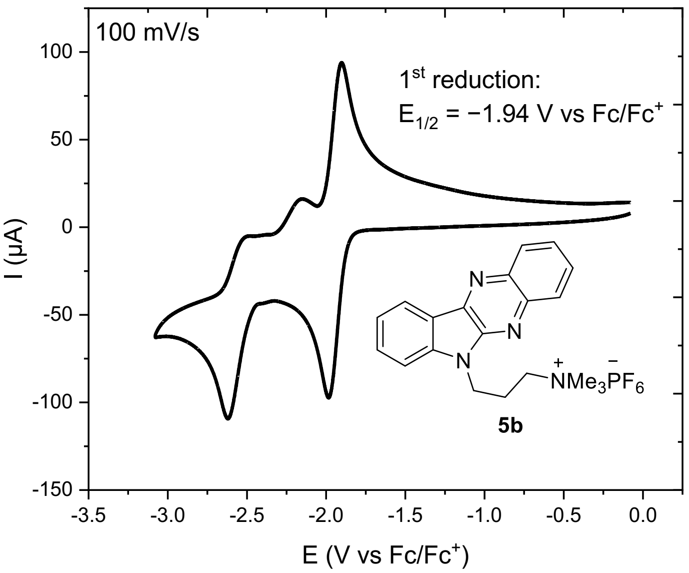

b

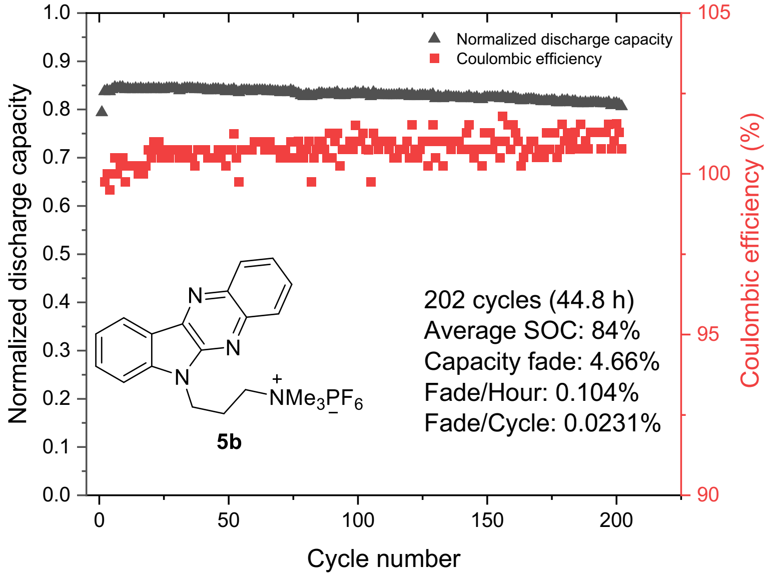

Figure SI4: (a). CV of **5b** (5 mM) in 0.5 M $TBAPF_6$/MeCN solution with glassy carbon working electrode at a scan rate of 100 mV/s. (b) Normalized discharge capacity and Coulombic efficiency versus cycle number of **5b**.

6-(2-methoxyethyl)-*6H*-indolo[2,3-*b*]quinoxaline (5c):

a

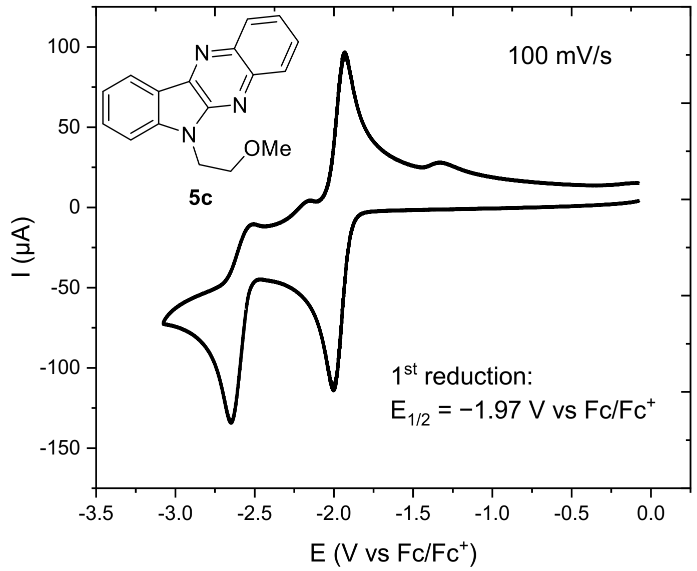

b

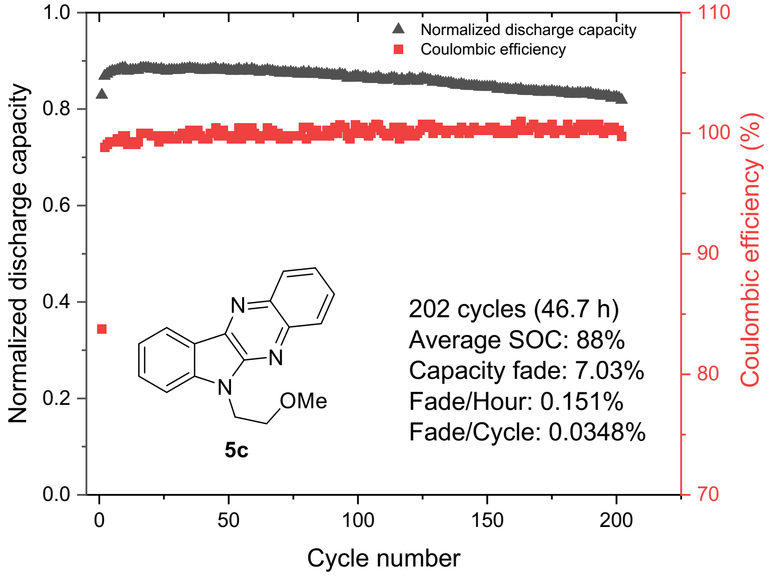

Figure SI5: (a). CV of **5c** (5 mM) in 0.5 M $TBAPF_6$/MeCN solution with glassy carbon working electrode at a scan rate of 100 mV/s. (b) Normalized discharge capacity and Coulombic efficiency versus cycle number of **5c**.

6-(2-(2-methoxyethoxy)ethyl)-*6H*-indolo[2,3-*b*]quinoxaline (5d):

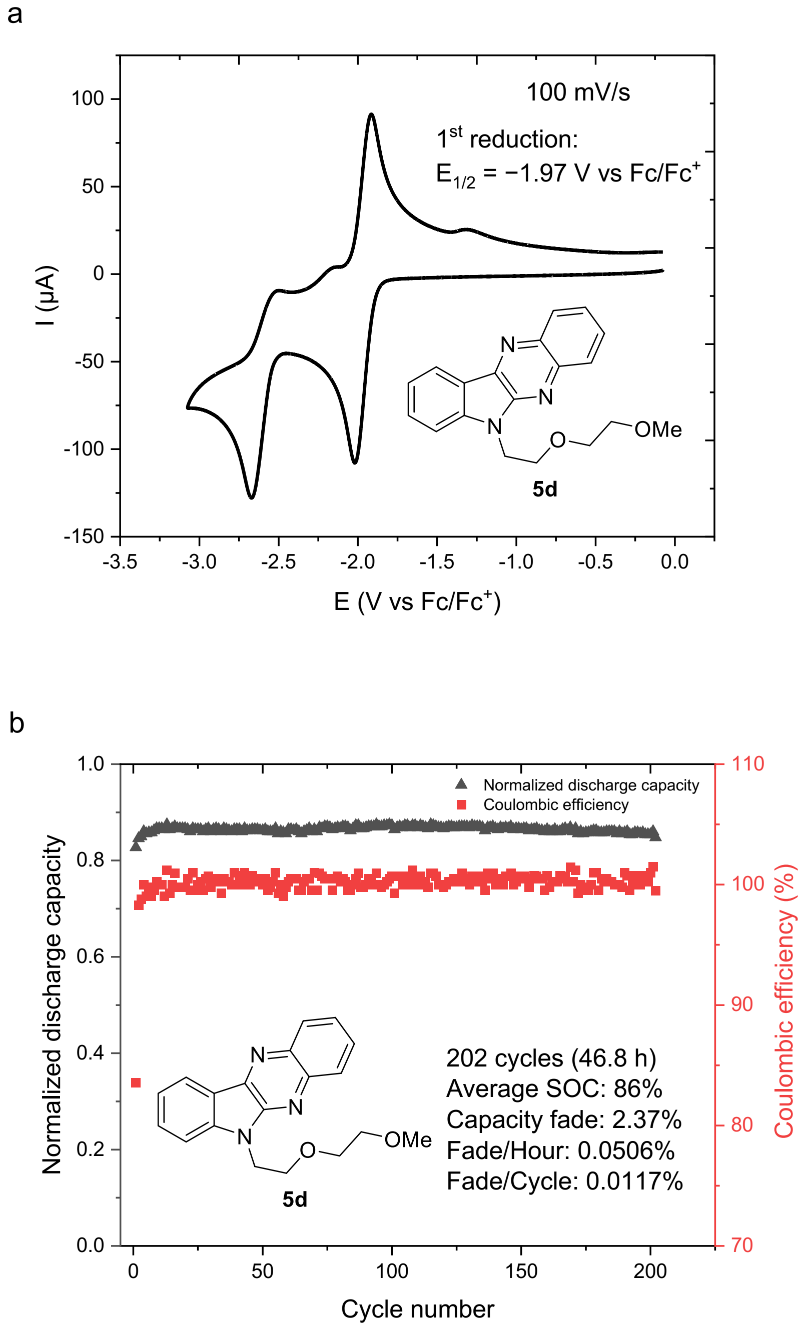

Figure SI6: (a). CV of **5d** (5 mM) in 0.5 M $TBAPF_6$/MeCN solution with glassy carbon working electrode at a scan rate of 100 mV/s. (b) Normalized discharge capacity and Coulombic efficiency versus cycle number of **5d**.

6-(2-(2-(2-methoxyethoxy)ethoxy)ethyl)-*6H*-indolo[2,3-*b*]quinoxaline (5e):

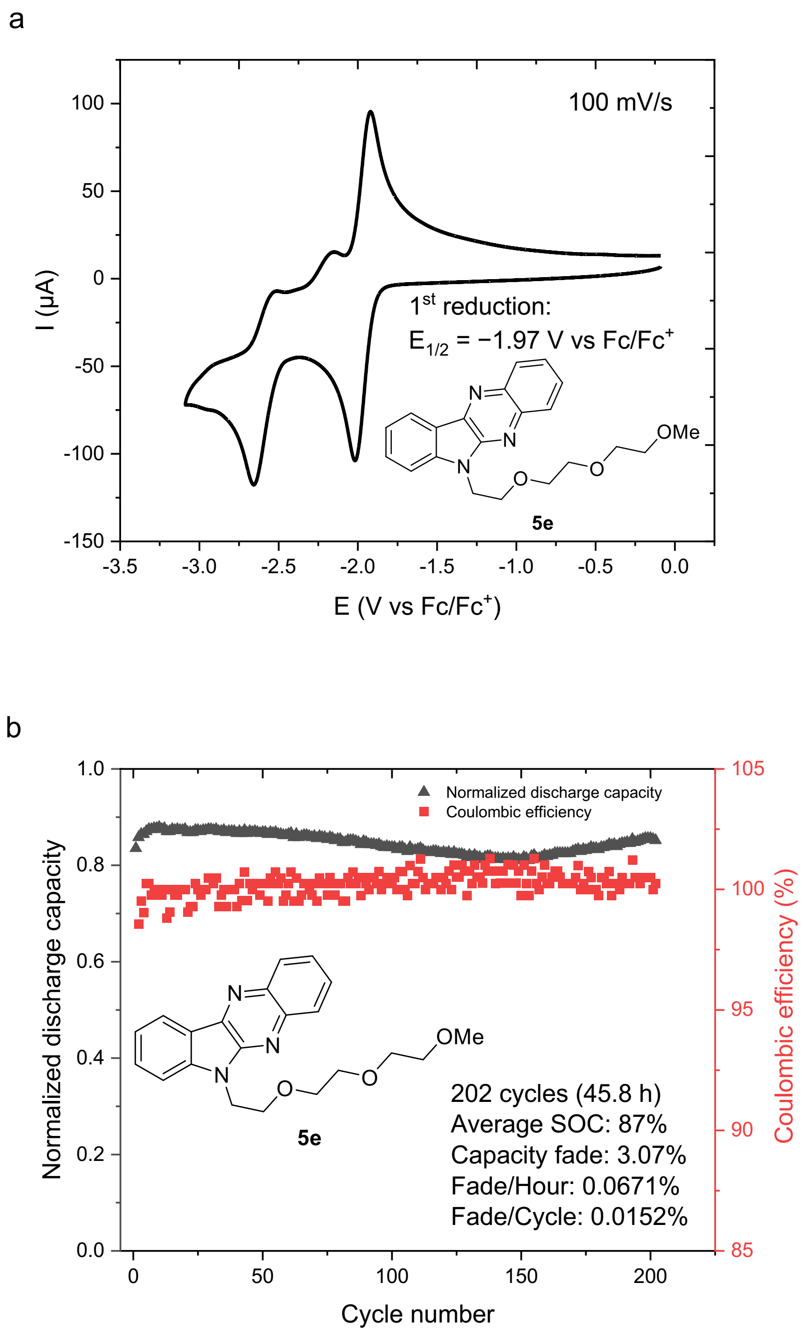

Figure SI7: (a). CV of **5e** (5 mM) in 0.5 M $TBAPF_6$/MeCN solution with glassy carbon working electrode at a scan rate of 100 mV/s. (b) Normalized discharge capacity and Coulombic efficiency versus cycle number of **5e**.

9-(*tert*-butyl)-6-methyl-6H-indolo[2,3-*b*]quinoxaline (5f):

a

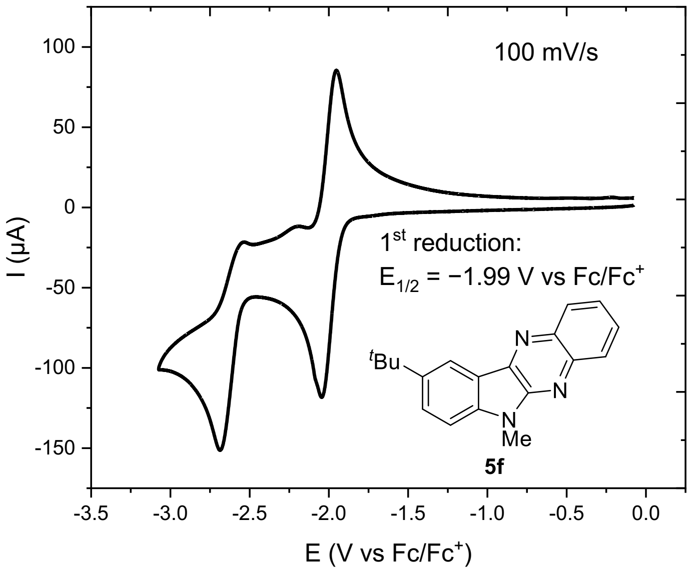

b

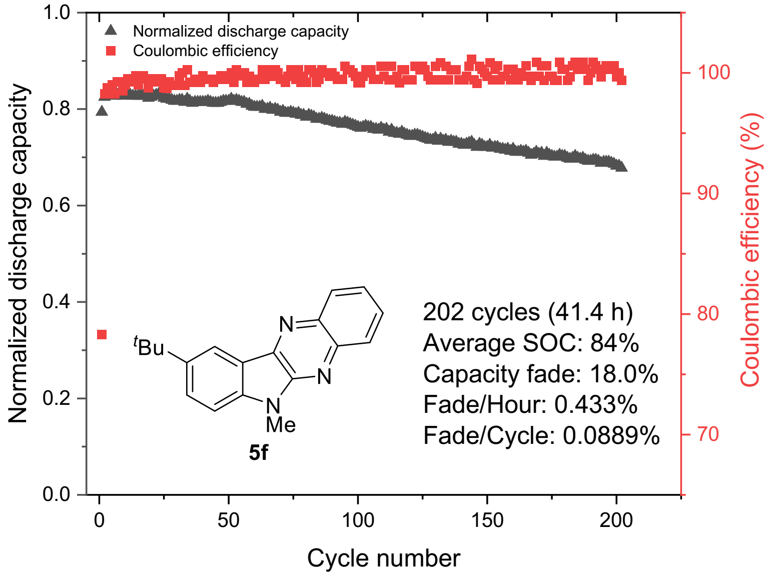

Figure SI8: (a). CV of **5f** (5 mM) in 0.5 M $TBAPF_6$/MeCN solution with glassy carbon working electrode at a scan rate of 100 mV/s. (b) Normalized discharge capacity and Coulombic efficiency versus cycle number of **5f**.

2/3-(*tert*-butyl)-6-methyl-*6H*-indolo[2,3-*b*]quinoxaline (5g):

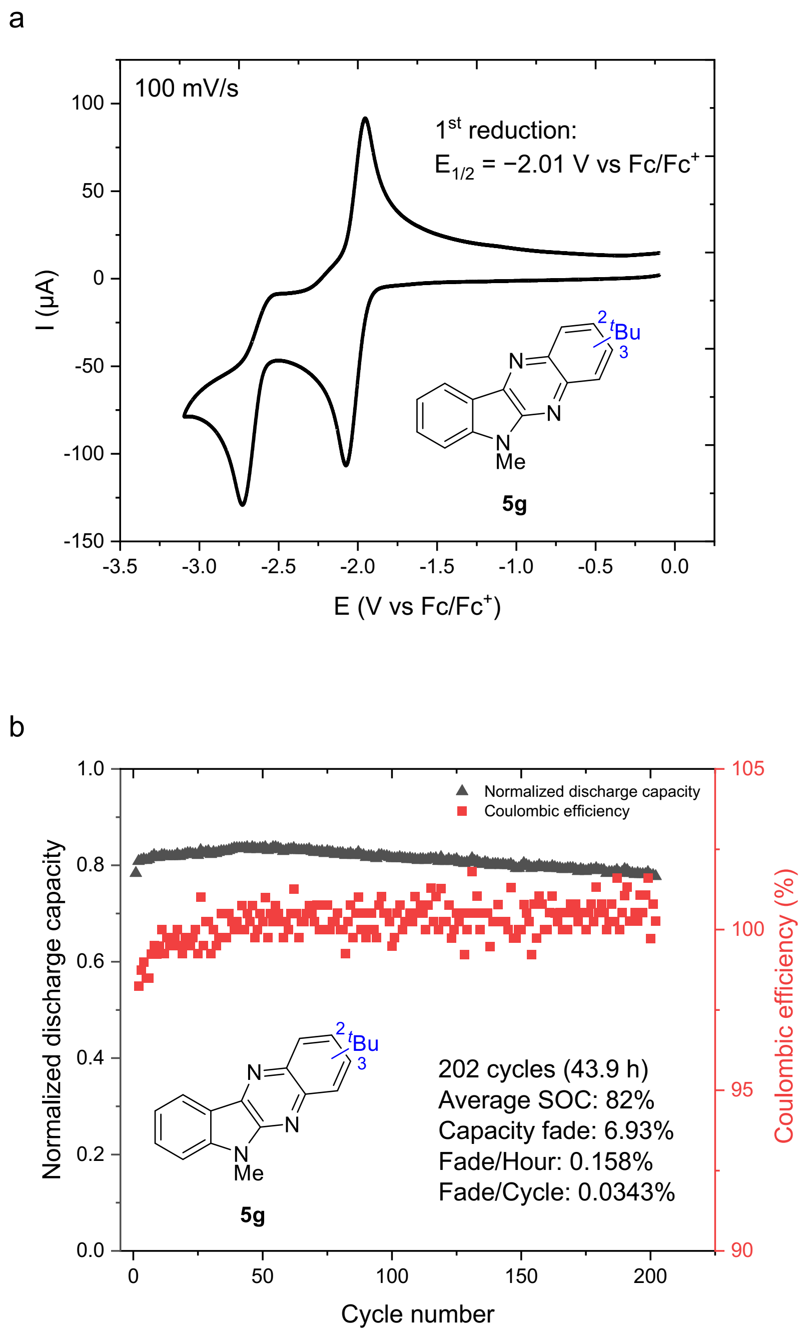

Figure SI9: (a). CV of **5g** (5 mM) in 0.5 M $TBAPF_6$/MeCN solution with glassy carbon working electrode at a scan rate of 100 mV/s. (b) Normalized discharge capacity and Coulombic efficiency versus cycle number of **5g**.

2/3-(*tert*-butyl)-6-(2-methoxyethyl)-*6H*-indolo[2,3-*b*]quinoxaline (5h):

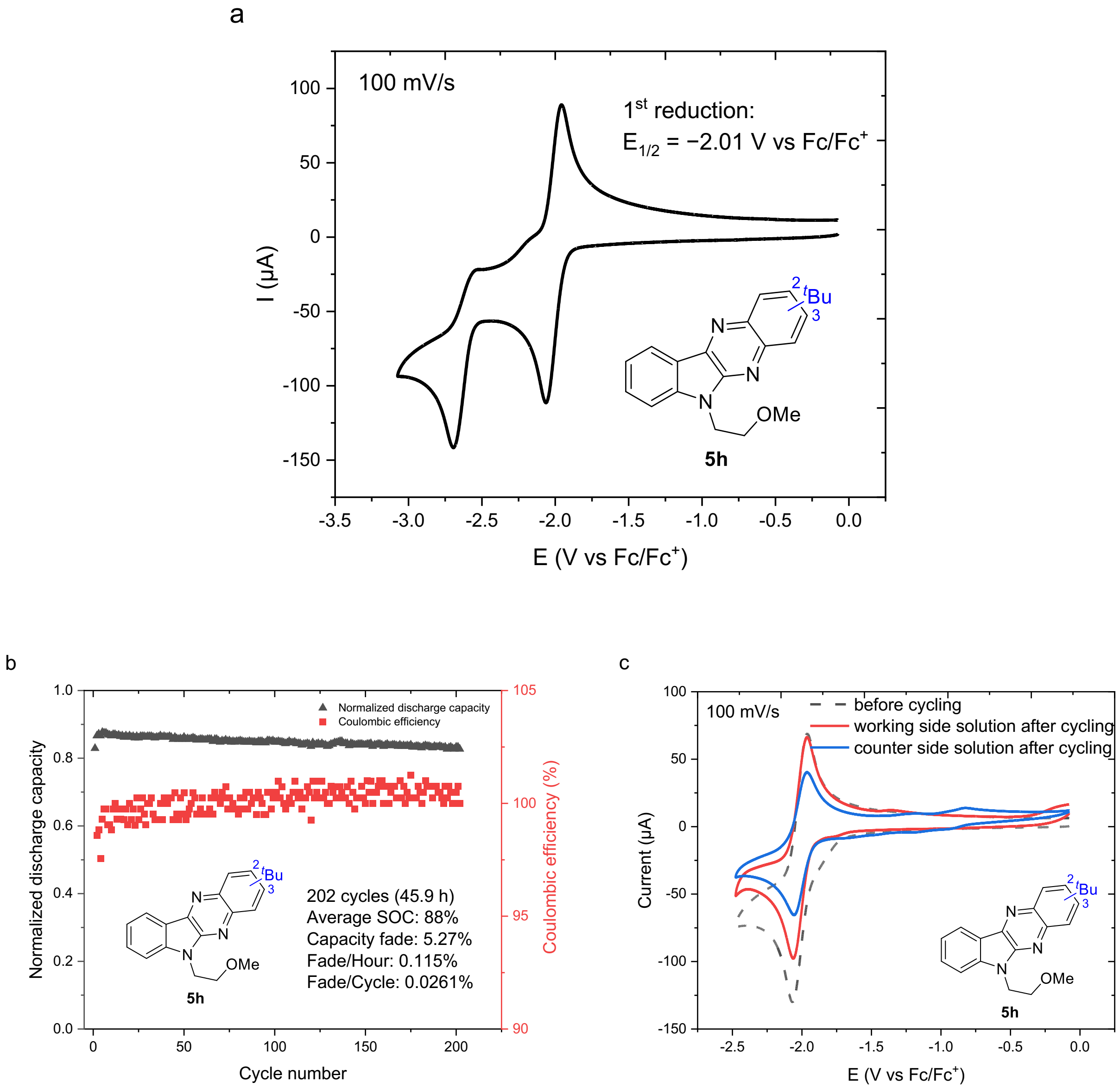

Figure SI10: (a). CV of **5h** (5 mM) in 0.5 M $TBAPF_6$/MeCN solution with glassy carbon working electrode at a scan rate of 100 mV/s. (b) Normalized discharge capacity and Coulombic efficiency versus cycle number of **5h**. (c). CV of working side solution and counter side solution before and after H-cell cycling in 0.5 M $TBAPF_6$/MeCN solution with glassy carbon working electrode at a scan rate of 100 mV/s.

2/3-(*tert*-butyl)-6-(2-(2-methoxyethoxy)ethyl)-*6H*-indolo[2,3-*b*]quinoxaline (5i):

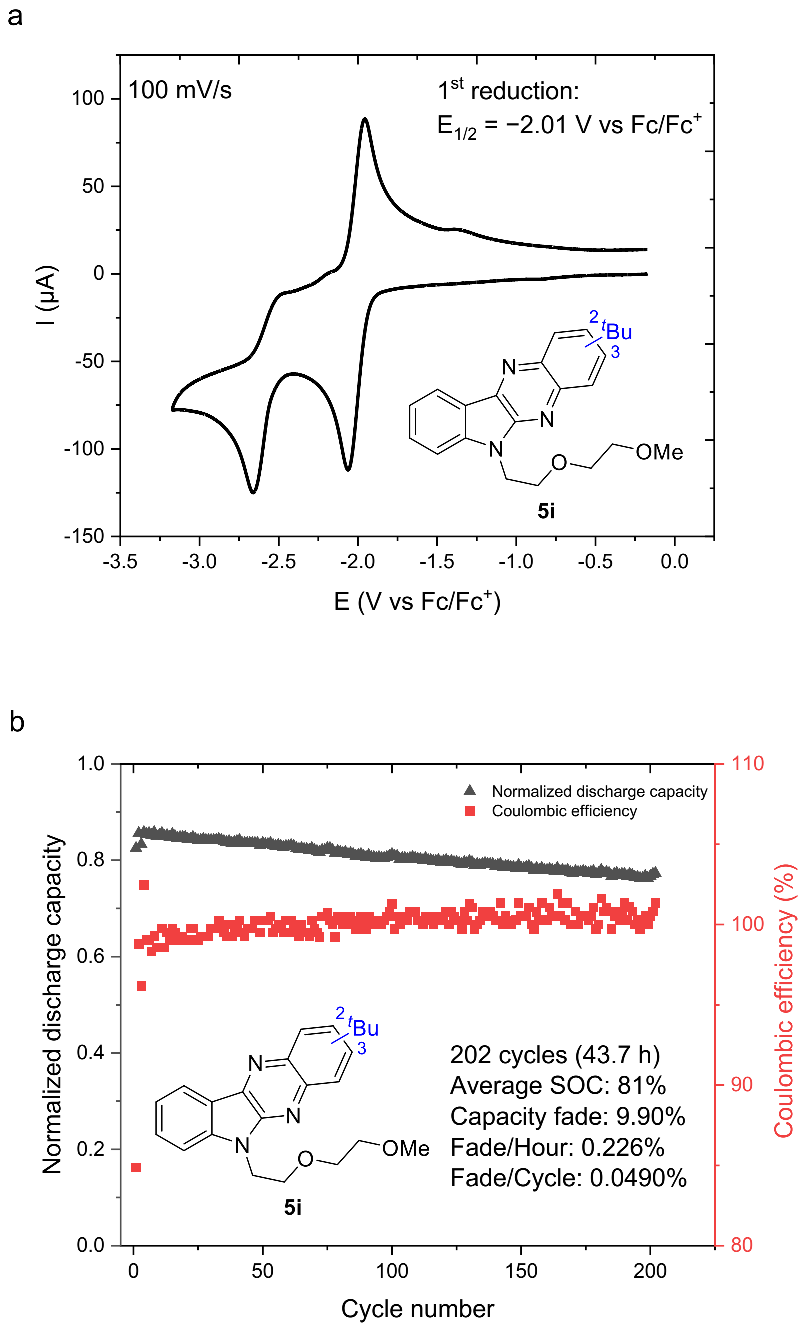

Figure SI11: (a). CV of **5i** (5 mM) in 0.5 M $TBAPF_6$/MeCN solution with glassy carbon working electrode at a scan rate of 100 mV/s. (b) Normalized discharge capacity and Coulombic efficiency versus cycle number of **5i**.

Mixed 5h (5 mM) and 10 (5 mM) solution as anolyte:

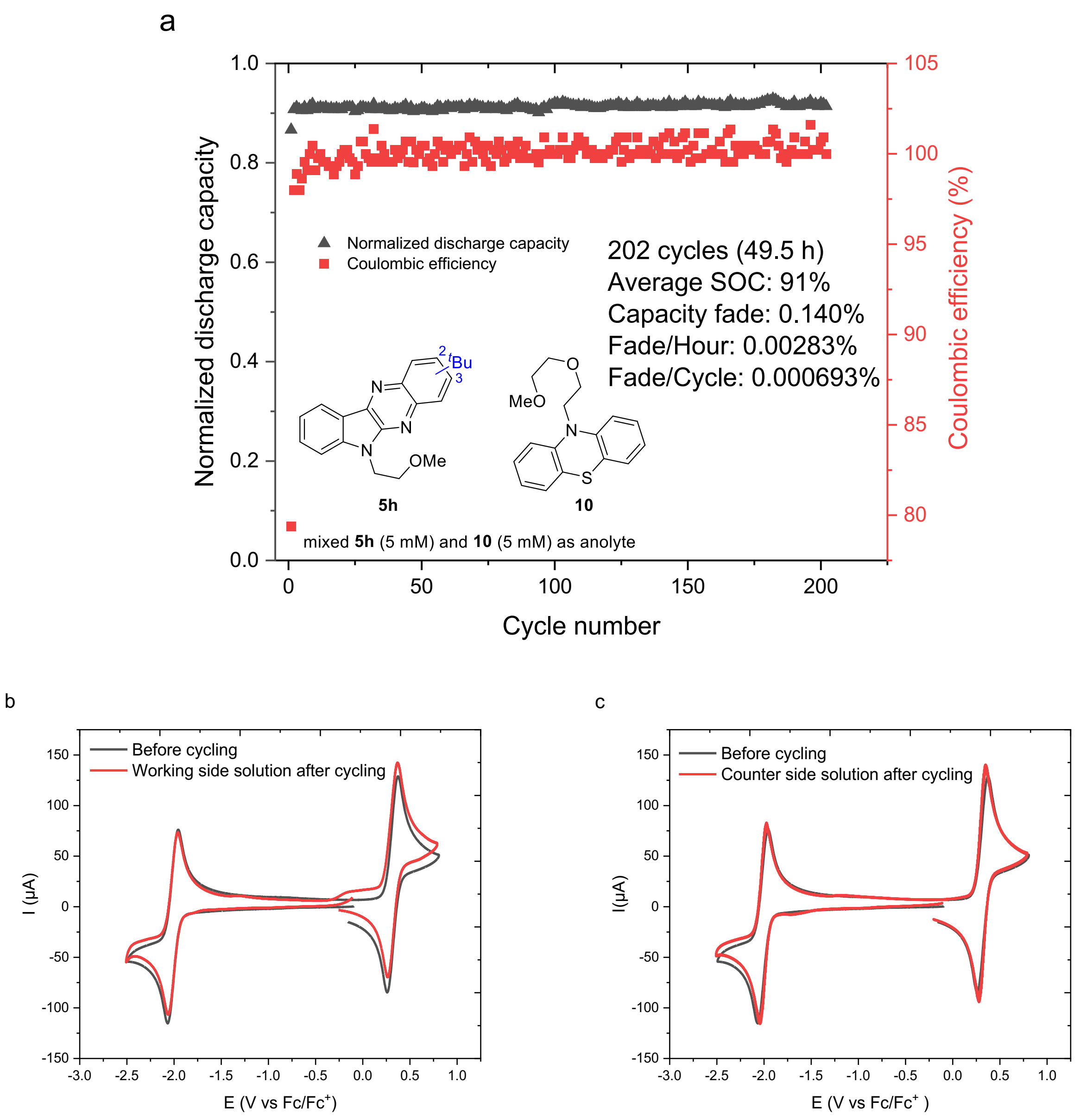

Figure SI12. (a) Normalized discharge capacity and Coulombic efficiency versus cycle number of mixed 5 mM **5h** and 5 mM **10** solution as anolyte (in 0.5 M $TBAPF_6$/MeCN) in a static H-cell cycling. CV of working side (b) and counter side (c) solution before and after H-cell cycling between **5h** and **5h**$^{\bullet-}$of mixed 5 mM **5h** and 5 mM **10** solution (5 mM in 0.5 M $TBAPF_6$/MeCN) with a glassy carbon working electrode at a scan rate of 100 mV/s.

Mixed 5h (5 mM) and 10 (5 mM) solution as catholyte:

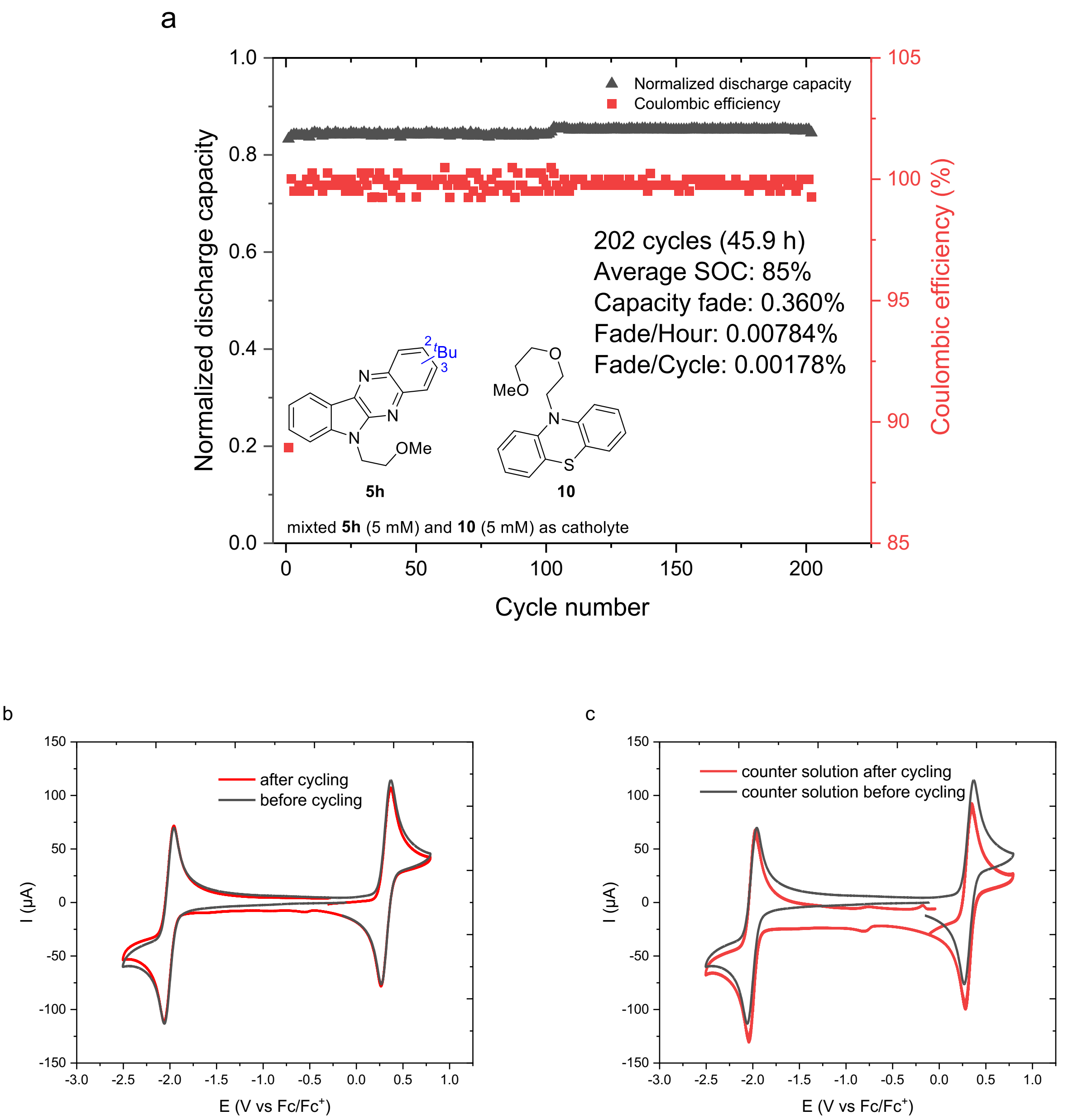

Figure SI13. (a) Discharge capacity and Coulombic efficiency versus cycle number of mixed 5 mM **5h** and 5 mM **10** solution as catholyte (in 0.5 M $TBAPF_6$/MeCN) in a static H-cell cycling. CV of working side (b) and counter side (c) solution before and after H-cell cycling between **5h** and **5h**$^{\bullet-}$ of mixed 5 mM **5h** and 5 mM **10** solution (5 mM in 0.5 M $TBAPF_6$/MeCN) with a glassy carbon working electrode at a scan rate of 100 mV/s.

0.05 M mixed 5h and 10 as electrolyte (Flow battery):

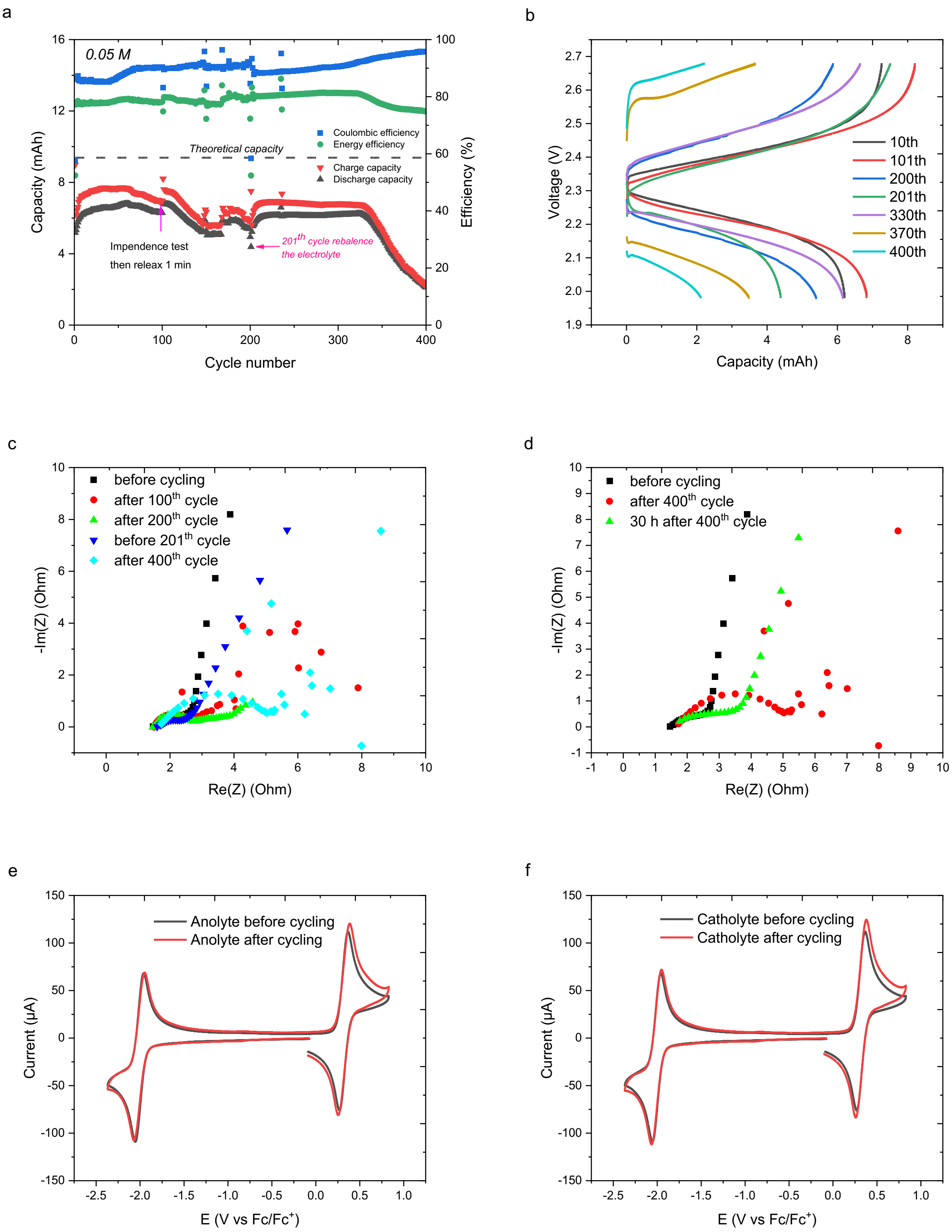

Figure SI14. (a) Charge and discharge capacity, Coulombic efficiency, and energy efficiency versus cycle number for flow cell cycling of 50 mM **5h** and 50 mM **10** in 0.5 M $TBAPF_6$/MeCN solution. (b) Charge-discharge curves of mixed **5h** (50 mM) and **10** (50 mM) at the current density of 10 mA cm^{-2}. (c)

Electrochemical impedance spectroscopy (EIS) on the flow cell before cycling and after 100th, and 200th, and 400th cycle. (d) Electrochemical impedance spectroscopy (EIS) on the flow cell before cycling, after 400th cycle, and relax for 30 h after 400th cycle. CVs of 5 mM diluted anolyte (e) and catholyte (f) before and after cycling the flow battery of 50 mM **5h** and 50 mM **10** in 0.5 M $TBAPF_6$/MeCN solution with a glassy carbon working electrode at a scan rate of 100 mV/s.

0.1 M mixed 5h and 10 as electrolyte (Flow battery):

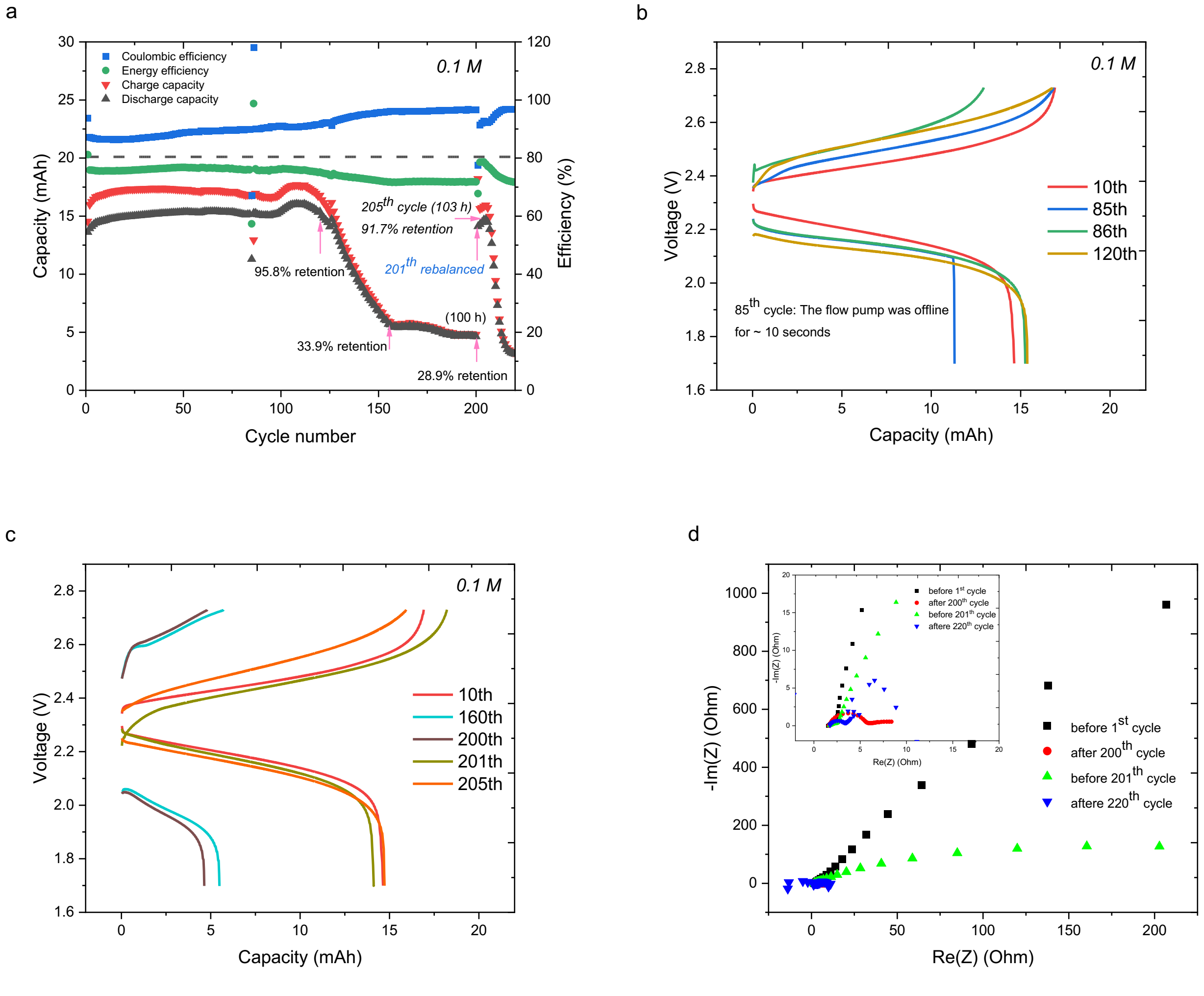

Figure SI15. (a) Charge and discharge capacity, Coulombic efficiency, and energy efficiency versus cycle number (1st to 220th) for flow cell cycling of 100 mM **5h** and 100 mM **10** in 0.5 M $TBAPF_6$/MeCN solution. (b) Charge-discharge curves of mixed **5h** (100 mM) and **10** (100 mM) for 10th, 85th, 86th and 120th cycles at the current density of 20 mA cm^{-2}. (c) Charge-discharge curves of mixed **5h** (100 mM) and **10** (100 mM) for 10th, 160th, 200th, 201th and 205th cycles at the current density of 20 mA cm^{-2}. (d)

Electrochemical impedance spectroscopy (EIS) on the flow cell before cycling and after 50th, and 75th cycles.

NMR analysis of post-cycling mixture:

The solvent was removed under vacuum from a mixture solution containing 0.1 M **5a** and 0.1 M **5h**. Subsequently, 30 mL of Et_2O and 10 mL of hexane were added to the mixture, which was vigorously stirred for 10 minutes before filtering. The filter cake was washed three times with a mixture of 8.0 mL Et_2O and hexane in a ratio of 3:1. The volatiles from the combined filtrate were removed under vacuum, and the resulting residue was directly subjected to ^1H NMR analysis. Please refer to S32 and S33 for ^1H NMR details.

0.25 M mixed 5h and 10 as electrolyte (Flow battery):

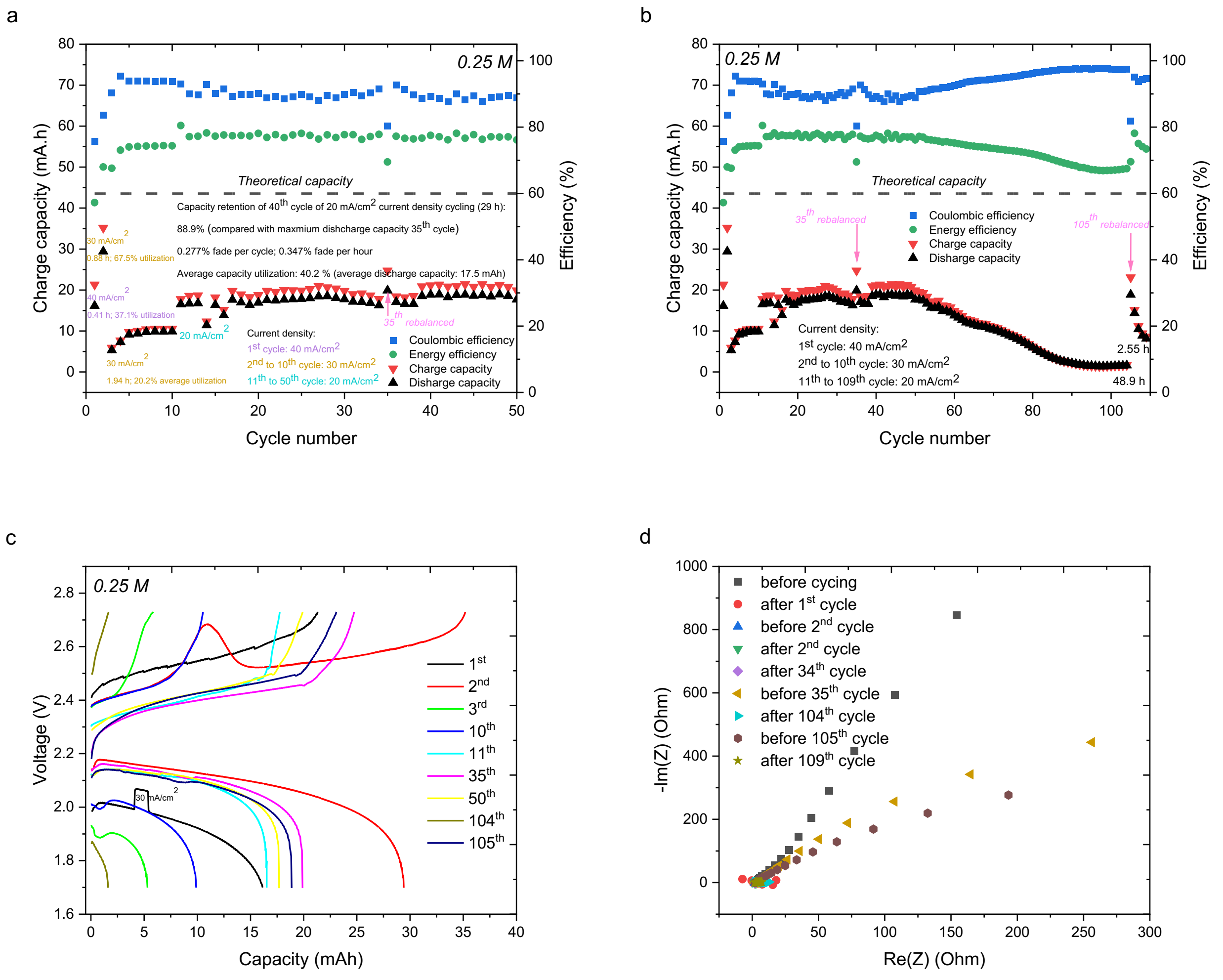

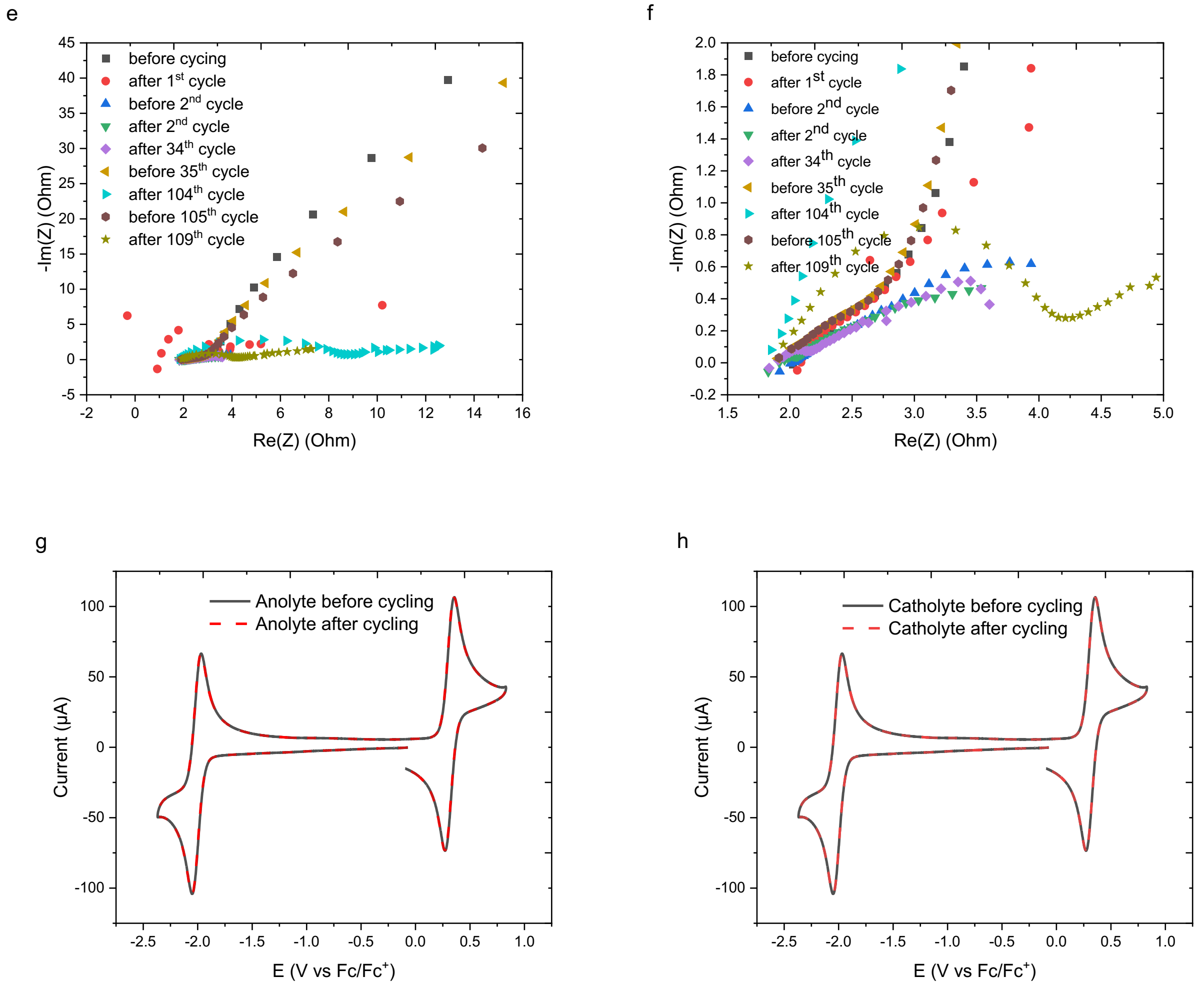

Figure SI16. Charge and discharge capacity, Coulombic efficiency, and energy efficiency versus cycle number (1st to 50th) (a) and (1st to 109th) (b) for flow cell cycling of 250 mM **5h** and 250 mM **10** in 0.5 M $TBAPF_6$/MeCN solution (6.5 mL anolyte/6.5 mL catholyte). (c) Charge-discharge curves of mixed **5h** (250 mM) and **10** (250 mM) for 1st (40 mA/cm^2); 2nd, 3rd, and 10th (30 mA/cm^2); 11th, 35th, 50th, 104th, and 105th (20 mA/cm^2) cycles. Electrochemical impedance spectroscopy (EIS) on the flow cell before and after cycling (d) and zoom in EIS (e and f). CVs of 5 mM diluted anolyte (g) and catholyte (h) before and after cycling the flow battery of 250 mM **5h** and 250 mM **10** in 0.5 M $TBAPF_6$/MeCN solution with a glassy carbon working electrode at a scan rate of 100 mV/s.

VI. Experiments Details and Methods of Electrochemical Kinetics Studies

Method A (through Cyclic Voltammetry Experiments):

Diffusion coefficients (D) for the neutral species were estimated using the Randles-Ševčík equation (eq. 1) by varying the scan rate of CV measurements between 25 and 500 mV/s. Plotting the cathodic and anodic current versus the square-root of the scan rate results in a linear relationship providing evidence for a diffusion limited chemically reversible process. The slope of this linear relation was used to estimate the diffusion coefficient for the neutral molecule and radical anion.

$$i_p = 0.4463\, nFAC\sqrt{\frac{nF\nu D}{RT}} \qquad \text{(eq. 1)}$$

In the Randles-Ševčík equation[2], i_p is the peak current in amps, n is the number of electrons transferred, F is Faraday constant (96485 C/mol e^-), A is the electrode area (0.071 cm^2), C is the bulk concentration of redox active species in mol/cm^3, R is the universal gas constant (8.3145 J/mol•K), T is the temperature in K, υ is the scan rate in V/s, and D is the diffusion coefficient in (cm^2/s).

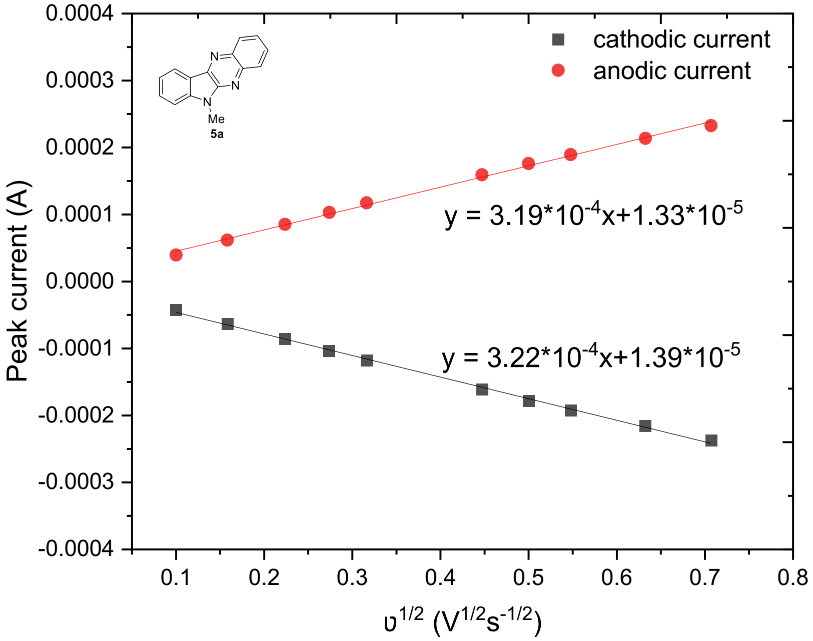

Figure SI17. Plots of anodic and cathodic peak current densities vs the square root of the sweep rate ($\nu^{1/2}$) for oxidation and reduction and linear fits used to determine diffusion coefficients of **5a** (5 mM).

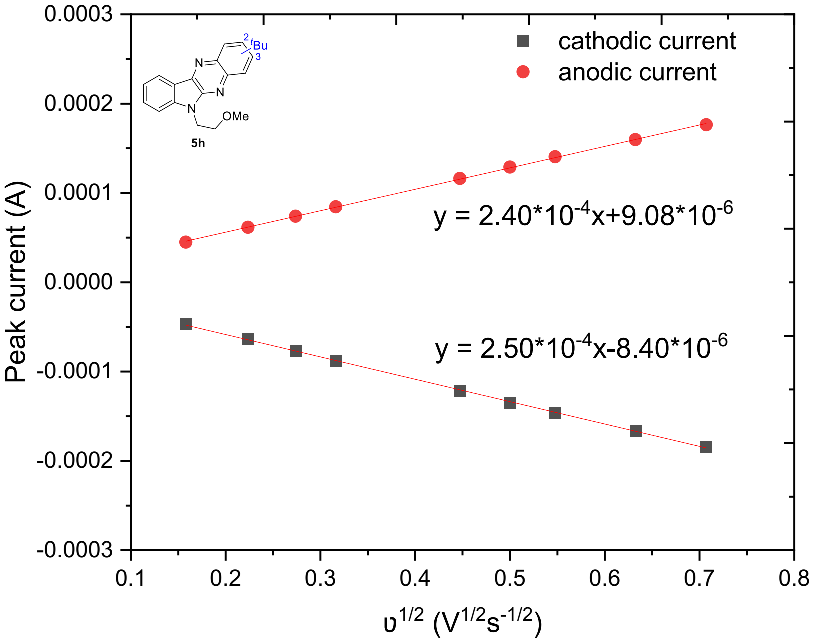

Figure SI18. Plots of anodic and cathodic peak current densities vs the square root of the sweep rate ($\nu^{1/2}$) for oxidation and reduction and linear fits used to determine diffusion coefficients of **5h** (5 mM).

The heterogeneous electron transfer rate constant (k_0) was calculated according to the Nicholson method[3] as modified by Magno and coworkers.[4] At each scan rate, the separation between the potential of the anodic and cathodic peaks, ΔEp, can be converted to a dimensionless number ψ using a "working curve" of the Nicholson paper (eq. 2).[3] The data is given in Table S1, S2. Plotting the resulting values of ψ versus the inverse square root of the scan rate (Figure S18, S19) gave a relationship from which the slope was used to determine the heterogeneous electron transfer rate constant k_0 according to eq 3.

$$\psi = \frac{(-0.6288+0.0021\Delta Ep)}{(1-0.017\ \Delta Ep)} \qquad \text{(eq. 2)}$$

$$ko = \psi\ (\frac{\pi DFnv}{RT})^{1/2} \qquad \text{(eq. 3)}$$

Where k_0 is the standard rate constant in cm s^{-1}, ψ is the Nicholson dimensionless parameter, π is a mathematical constant, D is the according diffusion coefficient in cm^2s^{-1}, n is the number of transferred electrons, F is the Faraday constant (96485 C mol^{-1}), □ν is the scan rate in V s^{-1}, R is the ideal gas constant (8.3145 J mol K^{-1}), T is the temperature in K and□ΔEp is the peak potential separation at the

according scan rate in mV. The electron-transfer rate constant was calculated from the slopes of the Nicholson parameter *vs* and eq.3

Table SI1. Cyclic Voltammetry Peak Separation Versus Scan Rate for 5a Nicholson Analysis

Entry	scan rate (V/s)	$\nu^{-1/2}$ ($V^{-1/2}s^{1/2}$)	Anodic peak (V)	Cathodic peak (V)	Peak separation (mV)	ψ
**1**	0.025	6.32456	-1.852	-1.943	91	0.80018
**2**	0.05	4.47214	-1.847	-1.948	101	0.58117
**3**	0.075	3.65148	-1.843	-1.954	111	0.44611
**4**	0.1	3.16228	-1.84	-1.956	116	0.3963
**5**	0.2	2.23607	-1.83	-1.965	135	0.26664
**6**	0.25	2	-1.828	-1.97	142	0.2338
**7**	0.3	1.82574	-1.823	-1.975	152	0.19545
**8**	0.4	1.58114	-1.818	-1.98	162	0.16454
**9**	0.5	1.41421	-1.816	-1.988	172	0.13909

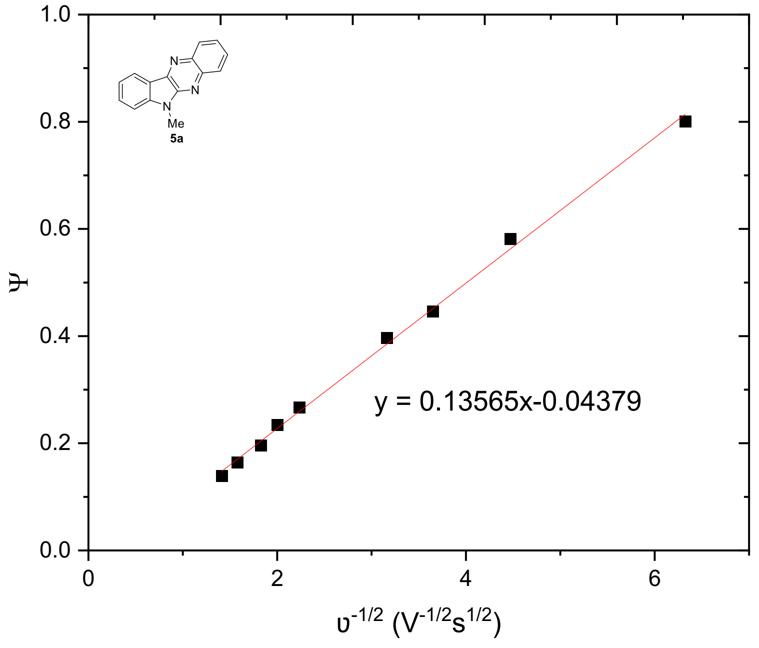

Figure SI19. Plot of Nicholson parameter ψ versus $\nu^{-1/2}$ of **5a** (5 mM). The slope was used to determine the heterogeneous electron transfer rate constant.

Table SI2. Cyclic Voltammetry Peak Separation Versus Scan Rate for 5h Nicholson Analysis

Entry	scan rate (V/s)	$\nu^{-1/2}$ ($V^{-1/2}s^{1/2}$)	Anodic peak (V)	Cathodic peak (V)	Peak separation (mV)	ψ
**1**	0.025	6.32456	-1.852	-1.934	82	1.15888
**2**	0.05	4.47214	-1.849	-1.937	88	0.89516
**3**	0.075	3.65148	-1.846	-1.939	93	0.74613
**4**	0.1	3.16228	-1.843	-1.941	98	0.63514
**5**	0.2	2.23607	-1.838	-1.95	112	0.4354
**6**	0.25	2	-1.834	-1.952	118	0.37873
**7**	0.3	1.82574	-1.834	-1.954	120	0.36231
**8**	0.4	1.58114	-1.829	-1.958	129	0.3
**9**	0.5	1.41421	-1.824	-1.964	140	0.24261

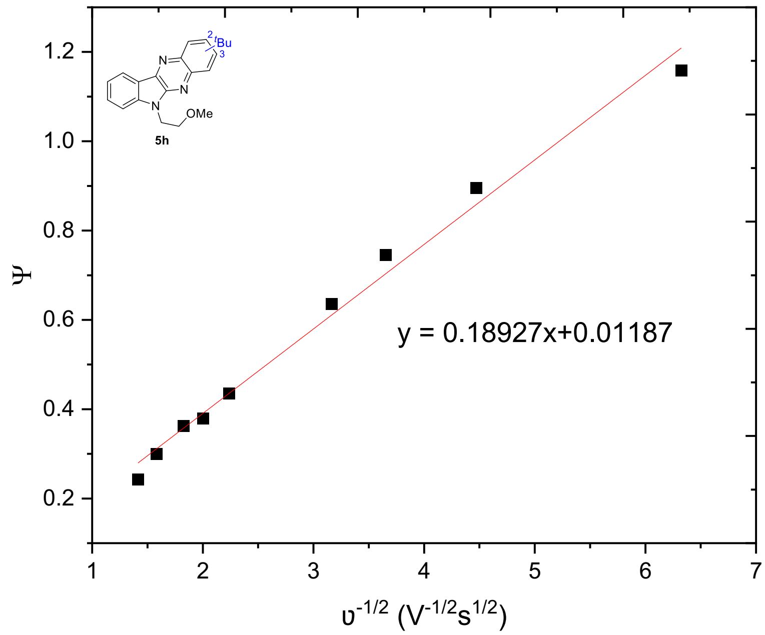

Figure SI20. Plot of Nicholson parameter ψ versus $\nu^{-1/2}$ of **5h** (5 mM). The slope was used to determine the heterogeneous electron transfer rate constant.

***Method B (through Linear Sweep Voltammetry Experiments)*:**

A solution of 2 mM **5a** or **5h** in 0.1 M $TBAPF_6$/MeCN was studied under positive Argon atmosphere. The rotation rates of RDE were 100, 225, 400, 625, 900, 1225, 1600, 2025 r.p.m. The diffusion coefficient (D) was calculated according to Levich equation (eq.4)[5]:

$$i_{\text{lim}} = 0.62nFAD^{2/3}\omega^{1/2}v^{-1/6}C \qquad \text{(eq. 4)}$$

where i_{lim} is limiting current, n is the number of electrons transferred (n = 1), F is the Faraday constant (*96485* C mol−1), A is the surface area of the working electrode (*0.19625* cm^2), C is molar concentration in 2×10^{-6} mol cm^{-3}, v is the kinetic viscosity in $cm^2\ s^{-1}$ (*0.00442* for 0.1 M $TBAPF_6$/MeCN)[6] and ω is the routing angular velocity in rad s^{-1}. The Koutecky–Levich plots at different overpotentials were extrapolated to get the kinetic current i_k according to the Koutecky–Levich equation (eq.5):

$$1/i = 1/i_k + 1/(0.62nFAD^{2/3}\omega^{1/2}v^{\ 1/6}C) \qquad \text{(eq. 5)}$$

The exchange current (i_0) can be obtained by fitting i_k to the Tafel plot at the overpotential of zero, from which the reaction rate constant (k_0) was determined according to the Butler–Volmer equation (eq.6):

$$i_0 = nFCk_0 \qquad \text{(eq. 6)}$$

Table SI3. Inverse Currents *1/i* for Angular Velocities, ω (rpm) and Potentials (V) Used to Construct the Koutecký-Levich Plot for 5a.

Potential (V)	100 rpm	225 rpm	400 rpm	625 rpm	900 rpm	1225 rpm	1600 rpm	2025 rpm
**-1.88**	-0.02196	-0.02765	-0.03325	-0.03848	-0.04267	-0.04656	-0.04869	-0.04882
**-1.92**	-0.06179	-0.079	-0.09573	-0.1103	-0.12305	-0.13351	-0.14129	-0.14449
**-1.93**	-0.0735	-0.09604	-0.11695	-0.1347	-0.15015	-0.16361	-0.17269	-0.17749
**-1.94**	-0.0857	-0.11259	-0.13775	-0.1595	-0.17945	-0.19411	-0.20689	-0.21259
**-1.95**	-0.09739	-0.12829	-0.15845	-0.1847	-0.20875	-0.22721	-0.24209	-0.24969
**-1.96**	-0.10589	-0.14399	-0.17845	-0.2098	-0.23595	-0.25901	-0.27729	-0.28839
**-1.97**	-0.11359	-0.15609	-0.19705	-0.2313	-0.26315	-0.28981	-0.31199	-0.32589
**-1.98**	-0.11959	-0.16719	-0.21175	-0.2501	-0.28615	-0.31881	-0.34409	-0.36339
**-1.99**	-0.12479	-0.17469	-0.22425	-0.2684	-0.30755	-0.34411	-0.37479	-0.39739

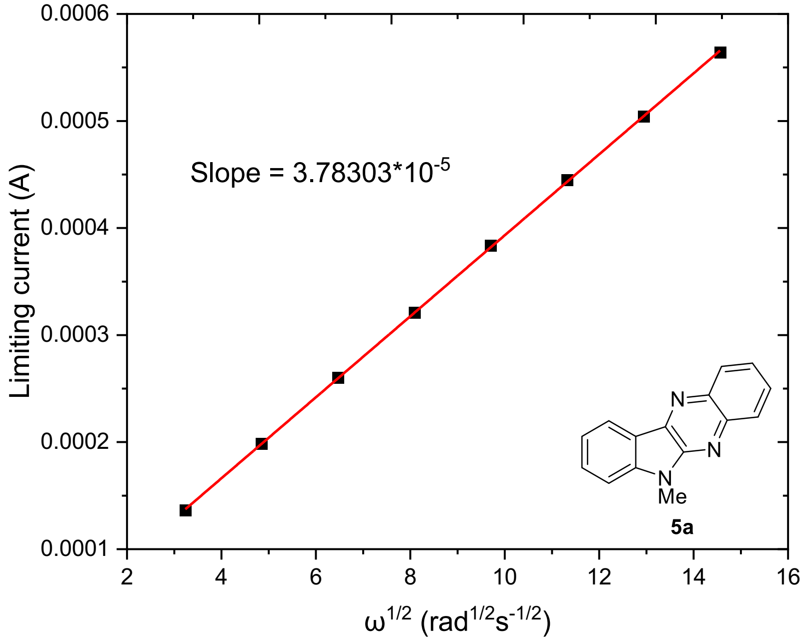

Figure SI21. Linearly fitted Levich plots of **5a** in 0.1 M $TBAPF_6$/MeCN.

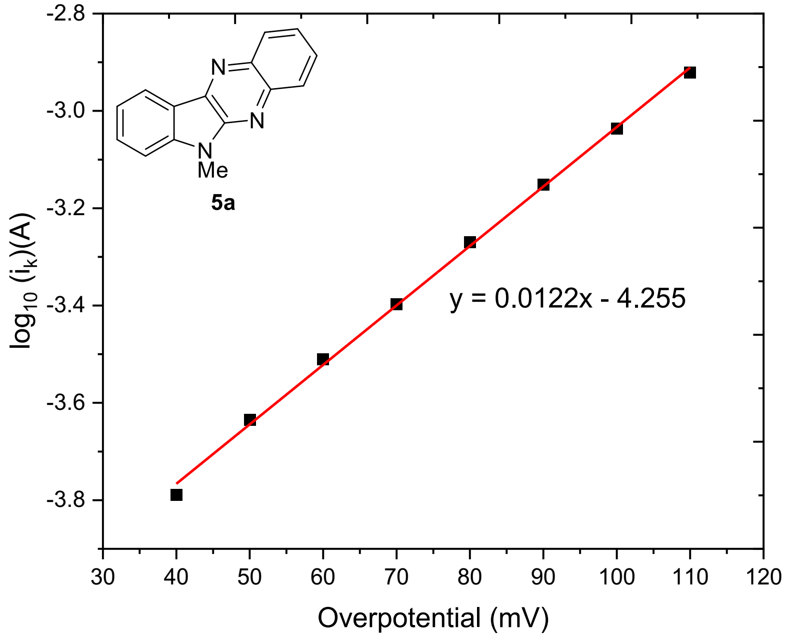

Figure SI22. Linearly fitted Tafel plots of **5a** based on Butler–Volmer equation as a function of overpotentials

Table SI4. Inverse Currents *1/i* for Angular Velocities, ω (rpm) and Potentials (V) Used to Construct the Koutecký-Levich Plot for 5h.

Potential (V)	100 rpm	225 rpm	400 rpm	625 rpm	900 rpm	1225 rpm	1600 rpm	2025 rpm
**-1.93**	-0.01757	-0.02137	-0.02476	-0.02576	-0.02657	-0.02892	-0.0346	-0.04959
**-1.97**	-0.05046	-0.06414	-0.07611	-0.08439	-0.0907	-0.09482	-0.1043	-0.12759
**-1.98**	-0.06008	-0.07752	-0.09321	-0.10419	-0.1129	-0.11972	-0.1298	-0.15609
**-1.99**	-0.06965	-0.09032	-0.11061	-0.12569	-0.1369	-0.14462	-0.1577	-0.18669
**-2.00**	-0.07734	-0.10362	-0.12811	-0.14729	-0.1599	-0.17172	-0.1868	-0.21749
**-2.01**	-0.08481	-0.11492	-0.14341	-0.16469	-0.1831	-0.19822	-0.2156	-0.24949
**-2.02**	-0.09042	-0.12432	-0.15671	-0.18329	-0.2063	-0.22382	-0.2442	-0.28139
**-2.03**	-0.09602	-0.13142	-0.16941	-0.19999	-0.2222	-0.24492	-0.2693	-0.31219
**-2.04**	-0.09862	-0.13762	-0.17851	-0.21069	-0.2411	-0.26612	-0.2946	-0.33959

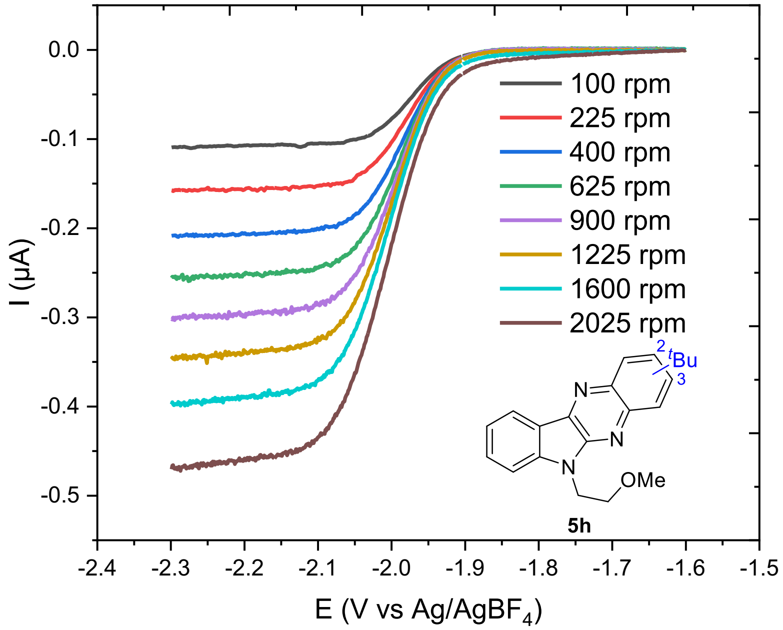

Figure SI23. Linear sweep voltammetry (LSV) of different rotation rates of the rotating disk electrode scans of 2 mM **5h** in 0.1 M $TBAPF_6$/MeCN.

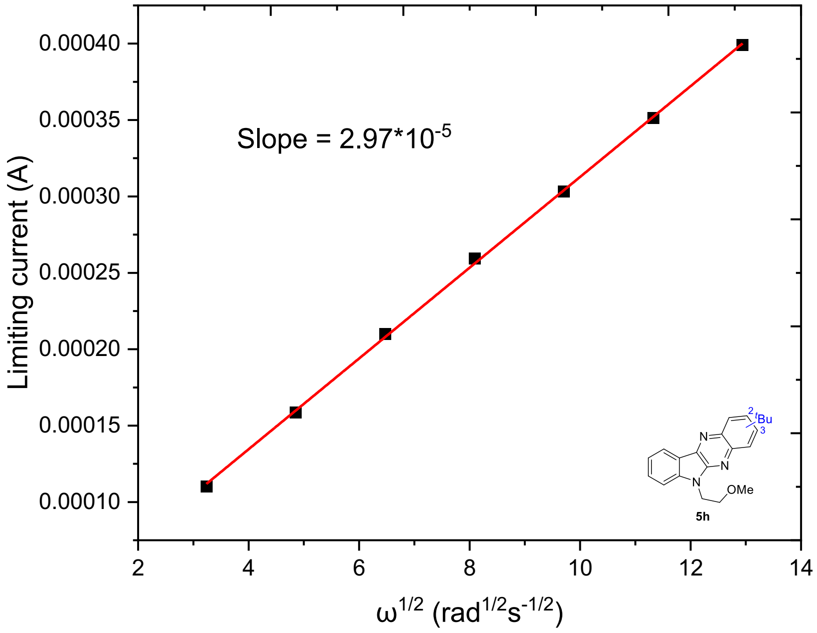

Figure SI24. Linearly fitted Levich plots of **5h** in 0.1 M $TBAPF_6$/MeCN.

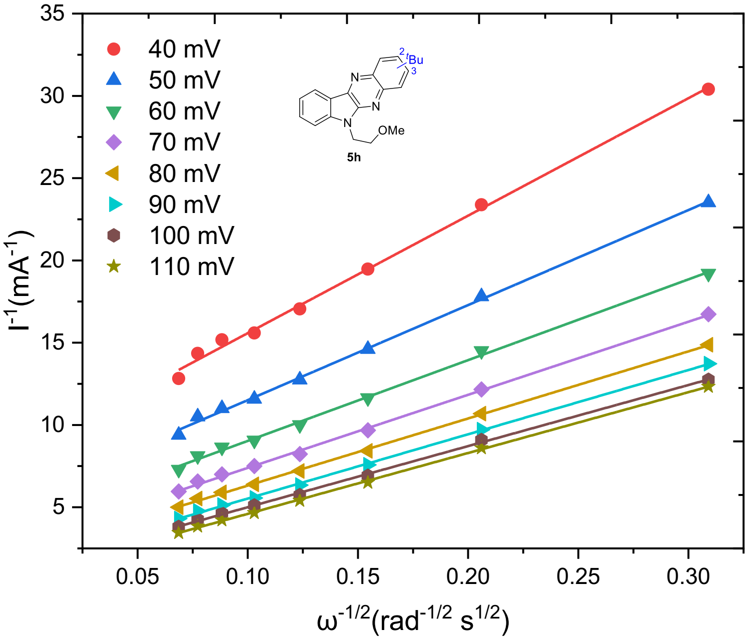

Figure SI25. Koutecky–Levich plots of **5h** in 0.1 M $TBAPF_6$/MeCN.

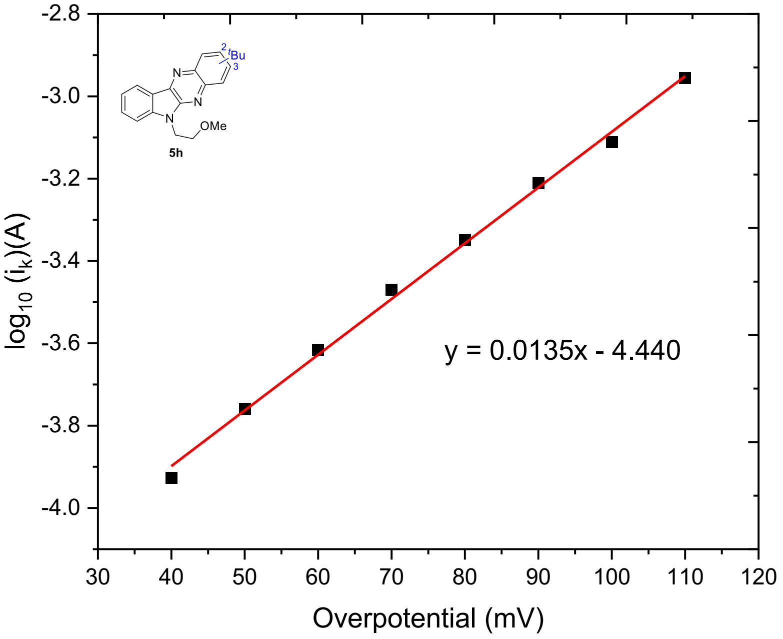

Figure SI26. Linearly fitted Tafel plots of **5h** based on Butler–Volmer equation as a function of overpotentials.

VII. Comparison of the Cell Performance of This Work with Other State-of-Art NARFBs Anolytes

Battery Type	Anolyte potential (electrolyte)/ cell potential	Capacity retention/% (cycles/time)	Capacity fade rate/% per cycle/per hour	Concentration/M	Solubility of anolyte/M (solvent)
Ref: 7<br>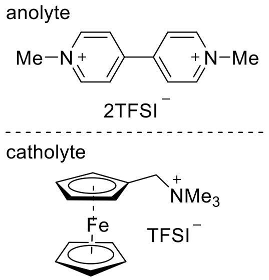	−0.79 V vs Fc/Fc$^+$<br>(1.0 M LiTFSI/MeCN)<br><br>1.05 V	91.8<br>(100/--)	0.082/--	0.1	0.98<br>(MeCN)
Ref: 8<br>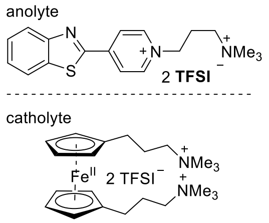	−1.19 V vs Fc/Fc$^+$<br>(0.1 M TBATFSI/MeCN)<br><br>1.16 V	65.7<br>(500/189 h)	0.0686/0.181	0.1	0.89<br>(MeCN)<br><br>0.34<br>(0.5 M TBATFSI/MeCN)
Ref: 9<br>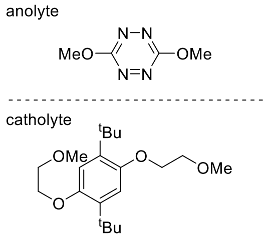	−1.20 V vs Fc/Fc$^+$<br>(0.5 M $TBAPF_6$/MeCN)<br><br>1.87 V	92<br>(50/17 h)	0.16/0.47	0.125	2.3<br>(MeCN)<br><br>0.79<br>(0.79 M $TBAPF_6$/MeCN)

Ref: 10<br>anolyte<br>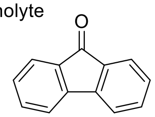<br>catholyte<br>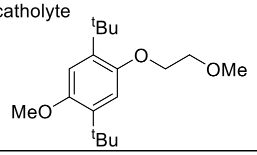	−1.64 V vs Ag/Ag^{+}<br>(1.0 M TEATFSI/MeCN)<br><br>2.37 V	20<br>(100/--)	0.8/--	0.5	2.0<br>(MeCN)<br><br>0.9<br>(1.2 M TEATFSI/MeCN)
Ref: 11<br>anolyte<br>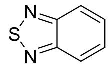<br>catholyte<br>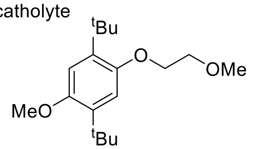	−1.58 V vs Ag/Ag^{+}<br>(1.0 M LiTFSI/MeCN)<br><br>2.36 V	83<br>(160/14 h)	0.11/1.2	0.1	5.7<br>(MeCN)<br><br>2.1<br>(2.1 M LiTFSI/MeCN)
Ref: 12<br>anolyte<br>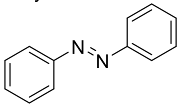<br>catholyte<br>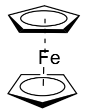	−1.79 V vs Ag/Ag^{+}<br>(0.5 M LiTFSI/DMF)<br><br>~1.8 V	86<br>(450/--)	0.031/--	0.1	>4.0<br>(MeCN)<br><br>>1.0<br>(1.0 M LiTFSI/DMF)

Ref: 13<br>anolyte<br>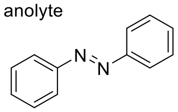<br>catholyte<br>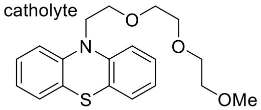	−1.76 V vs Fc/Fc^+<br>(0.5 M $TBAPF_6$/MeCN)<br><br>2.08 V	93.3<br>(50/--);<br><br>70.8<br>(80--)	0.13/--<br><br>0.75/--	0.1	--
Ref: 14<br>anolyte<br>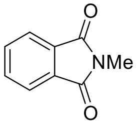<br>catholyte<br>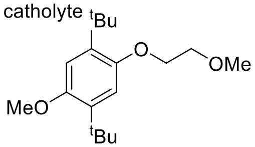	−1.79 V vs Ag/Ag^+<br>(1.0 M LiTFSI/DME)<br><br>2.30 V	80<br>(50/--)	0.24/--	0.3	~0.7<br>(MeCN)
Ref: 15<br>anolyte<br>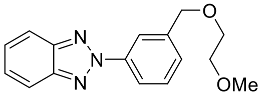<br>catholyte<br>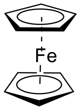	−2.30 V vs Fc/Fc^+<br>(0.25 M LiTFSI/DMF)<br><br>2.30 V	86<br>(50/17.5)	0.24/0.80	0.1	Miscible<br>(DMF)<br><br>Miscible<br>(0.5 M $TEABF_4$/DMF)

Ref: 16 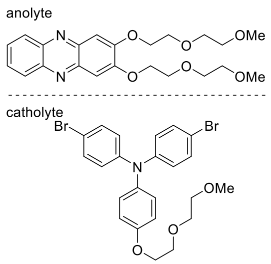	−1.72 V vs Ag/Ag$^+$<br>(0.1 M $TBABF_4$/MeCN)<br><br>2.30 V	65<br>(20/--)	1.75/--	0.01	2.5<br>(MeCN)<br><br>1.0<br>(0.5 M $TBABF_4$/MeCN)
Ref: 17 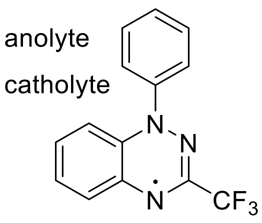	−1.03 V vs Fc/Fc$^+$<br>(0.1 M $TBAPF_6$/MeCN)<br><br>0.94 V	98<br>(100/32.3)	0.02/0.06	0.1	0.64<br>(MeCN)
Ref: 18 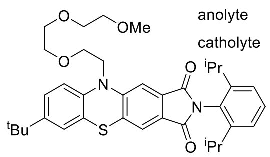	−1.83 V vs Fc/Fc$^+$<br>(0.5 M $TBAPF_6$/MeCN)<br><br>2.31 V	68.6<br>(275/~57)	0.114/~0.55	0.025	1.8<br>(MeCN)

Ref: 19<br>anolyte<br>catholyte<br>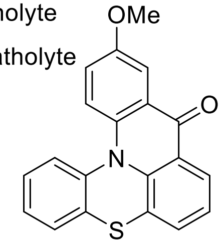	−2.04 V vs Ag/Ag$^+$<br>(0.1 M TBATFSI/MeCN)<br><br>2.76 V	73.8<br>(50/--)	0.524/--	0.025	--
This work<br>anolyte<br>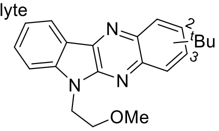<br>catholyte<br>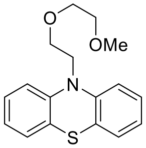	***−2.01 V vs Fc/Fc$^+$***<br>***(0.5 M TBAPF$_6$/MeCN)***<br><br>***2.33 V***	***95.8***<br>***(120/75.1)***	***0.035/0.056***	***0.1***	***>2.7***<br>***(MeCN)***<br><br>***>1.2***<br>***(0.5 M TBAPF$_6$/MeCN)***

VIII. Density Functional Theory Calculations

If not stated otherwise, the Q-Chem[20] program suite (version 6.0) was employed for all density functional theory calculations. Initial geometries for the neutral species were relaxed with the semiempirical method GFN2-xTB[21] using the XTB[22] program (version 6.4.1). For **5h** with a moderately flexible side chain, the lowest energy conformer was obtained with the Conformer–Rotamer Ensemble Sampling Tool (CREST[23]). Solvation contributions were implicitly included through the analytical linearized Poisson-Boltzmann (ALPB[24]) solvation model.

Molecular geometries were further optimized in both redox states with the ωB97X-D[25] functional in the def2-SVPD[26,27] basis set. The effects of acetonitrile solvent were approximately included via a polarizable continuum model, CPCM.[28] Cavities were constructed with atomic radii from the universal force field[29], and discretization was achieved using a high-quality Lebedev grid with 302 grid points. To verify that an optimized structure corresponds to a true minimum in orbital space, we performed a vibrational analysis based on a single-point Hessian calculation[30] at the GFN2-xTB/ALPB(MeCN) level of theory, and confirmed that there were no imaginary modes. Thermal contributions were obtained following Grimme's modified rigid-rotor-harmonic-oscillator (mRRHO[31]) approach.

Subsequent single-point calculations employed a variety of density functionals (B97-D3(BJ)[32–34], PBE-D4[35,36], TPSSh-D4[36,37], B3LYP-D4[36,38,39], PBE0-D4[36,40], PW6B95-D4[36,41], MPW1B95-D3(BJ)[33,36,42], MPWB1K-D4[36,42], PWB6K-D3(BJ)[33,34,41], ωB97M-V[43], ωB97X-V[44], ωB97X-D3[45], ωB97X-D[25]) and the def2-TZVPPD[26,27] basis set. In all Q-Chem calculations, numerical integration for the exchange-correlation functional utilized the SG-2 standard grid[46], whereas non-local correlation contributions were obtained using the SG-1 standard grid[47]. All molecular orbitals (MOs) were plotted using an isovalue of 0.05 Å^{-3}. Redox potentials are reported versus the *calculated* potential of the Fc/Fc^{+} redox couple to increase favorable error cancellation. Identical activity and diffusion coefficients for the oxidized and

reduced species are assumed, leading to the half-potential being formally identical to the standard redox potential obtained from the difference in Gibbs energies.[48] Population analysis based on natural bond orbitals[49] (NBOs) was performed by calling the NBO[50] package (version 5) incorporated in Q-Chem. In addition, Mulliken[51] charges and condensed spin densities, and CHELPG[52,53] charges were calculated.

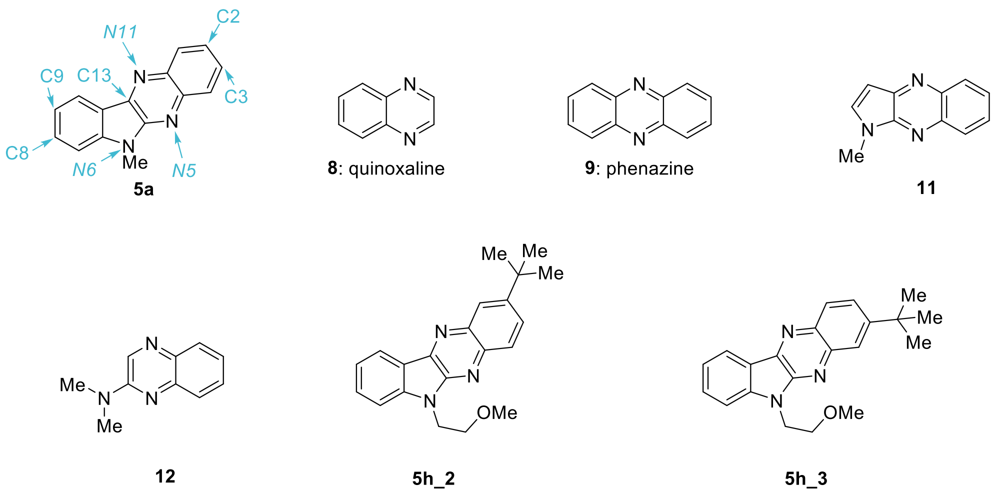

Figure SI27. Chemical structure of all computationally investigated species (**5a**, **8**, **9**, **11**, **12**, 2-*tert*-butyl-**5h** (**5h_2**), and 3-*tert*-butyl-**5h** (**5h_3**)).

To identify an appropriate density functional for predictive calculations, we first benchmarked the reduction potentials of **8**, **9**, and **5a** against the experimental half potentials (Table SI5). Multiple density functionals such as TPSSh-D4, PW6B95-D4, MPW1B95-D3(BJ), and the range-separated hybrid functionals adequately reproduce the experimental redox potentials. We chose to use the hybrid meta-GGA functional TPSSh-D4 in the following, given that TPSSh only contains one empirical parameter in the form of the admixture of exact exchange[37].

Table SI5. Calculated first reduction potential ($E_{1/2}$) of **8**, **9**, and **5a** versus the calculated Fc/Fc$^+$ redox couple in V for different density functionals. The deviation from the experimental reference ($\Delta E_{1/2}$) is also given. MAD is the mean absolute deviation ($N^{-1} \sum_{i=1}^{N} |\Delta E_{1/2,\,i}|$) and AMAX refers to the absolute maximal deviation observed.

Density Functional	$E_{1/2}$(**8**)	$\Delta E_{1/2}$(**8**)	$E_{1/2}$(**9**)	$\Delta E_{1/2}$(**9**)	$E_{1/2}$(**5a**)	$\Delta E_{1/2}$(**5a**)	MAD	AMAX
B97-D3(BJ)	-2.17	-0.10	-1.68	-0.08	-2.11	-0.14	0.11	0.14
PBE-D4	-2.23	-0.16	-1.73	-0.13	-2.16	-0.19	0.16	0.19
TPSSh-D4	-2.04	0.03	-1.54	0.06	-1.98	-0.01	0.03	0.06
B3LYP-D4	-1.93	0.14	-1.44	0.16	-1.88	0.09	0.13	0.16
PBE0-D4	-1.91	0.16	-1.39	0.21	-1.84	0.13	0.17	0.21
PW6B95-D4	-2.05	0.02	-1.53	0.07	-1.98	-0.01	0.03	0.07
MPW1B95-D3(BJ)	-2.06	0.01	-1.53	0.07	-1.98	-0.01	0.03	0.07
MPWB1K-D4	-1.96	0.11	-1.42	0.18	-1.88	0.09	0.13	0.18
PWB6K-D3(BJ)	-1.92	0.15	-1.38	0.22	-1.84	0.13	0.17	0.22
ωB97M-V	-2.11	-0.04	-1.57	0.03	-2.04	-0.07	0.05	0.07
ωB97X-V	-2.09	-0.02	-1.57	0.03	-2.03	-0.06	0.04	0.06
ωB97X-D3	-2.07	0.00	-1.55	0.05	-2.02	-0.05	0.04	0.05
ωB97X-D	-2.04	0.03	-1.52	0.08	-1.99	-0.02	0.04	0.08
experiment	-2.07	-	-1.60	-	-1.97	-	-	-

Table SI6. Calculated first reduction potentials ($E_{1/2}$) of all investigated compounds versus the calculated Fc/Fc$^+$ redox couple in V at the TPSSh-D4/def2-TZVPPD/CPCM(MeCN)// ωB97X-D/def2-SVPD/CPCM(MeCN) level of theory in comparison to experimental values.

Compound	$E_{1/2}$ (calculated)	$E_{1/2}$ (experimental)
8	-2.04	-2.07
9	-1.54	-1.60
5a	-1.98	-1.97
5h_2	-2.03	-2.01
5h_3	-2.06	-2.01
11	-2.13	n/a
12	-2.38	n/a

Having demonstrated that the computational protocol outlined above can predict reduction potentials to a very high accuracy vs experiment, we consider two new compounds, **11** and **12** (Figure SI27). The calculated potentials are -2.13 V and -2.38 V vs Fc/Fc$^+$, respectively, and are shown relative to the other structures in Table SI4. As outlined in the main text, extending the π-system, e.g., going from **8** to **9** or

from **11** to **5a**/**5h**, makes the reduction potential more positive due to the stabilization of the reduced species. On the other hand, introducing an electron-donating group such as a trivalent nitrogen atom into the π-system leads to a much lower potential (**8** to **12**) despite extending the π-system due to hyperconjugative effects and the lone pair situated at the nitrogen atom (*vide infra*). Interestingly, this shift is significantly larger going from **9** to **11**, presumably, since the size of the π-system is almost maintained.

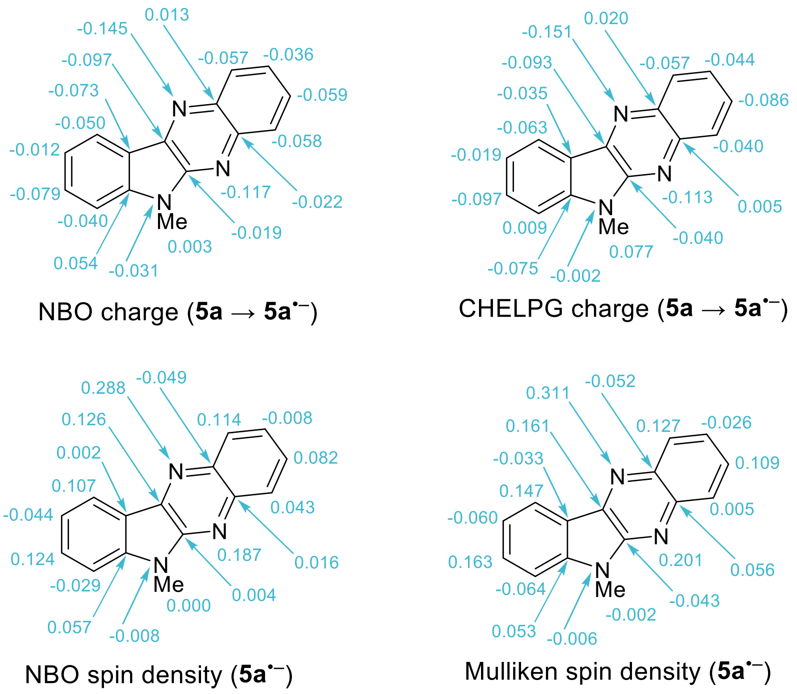

Figure SI 28. Charge and spin density changes upon reduction from 5a to 5a•−. Both NBO and CHELPG charges, as well as condensed NBO and Mulliken spin densities, yield a consistent picture where the excess electron is more localized on N5, N11, and C13.

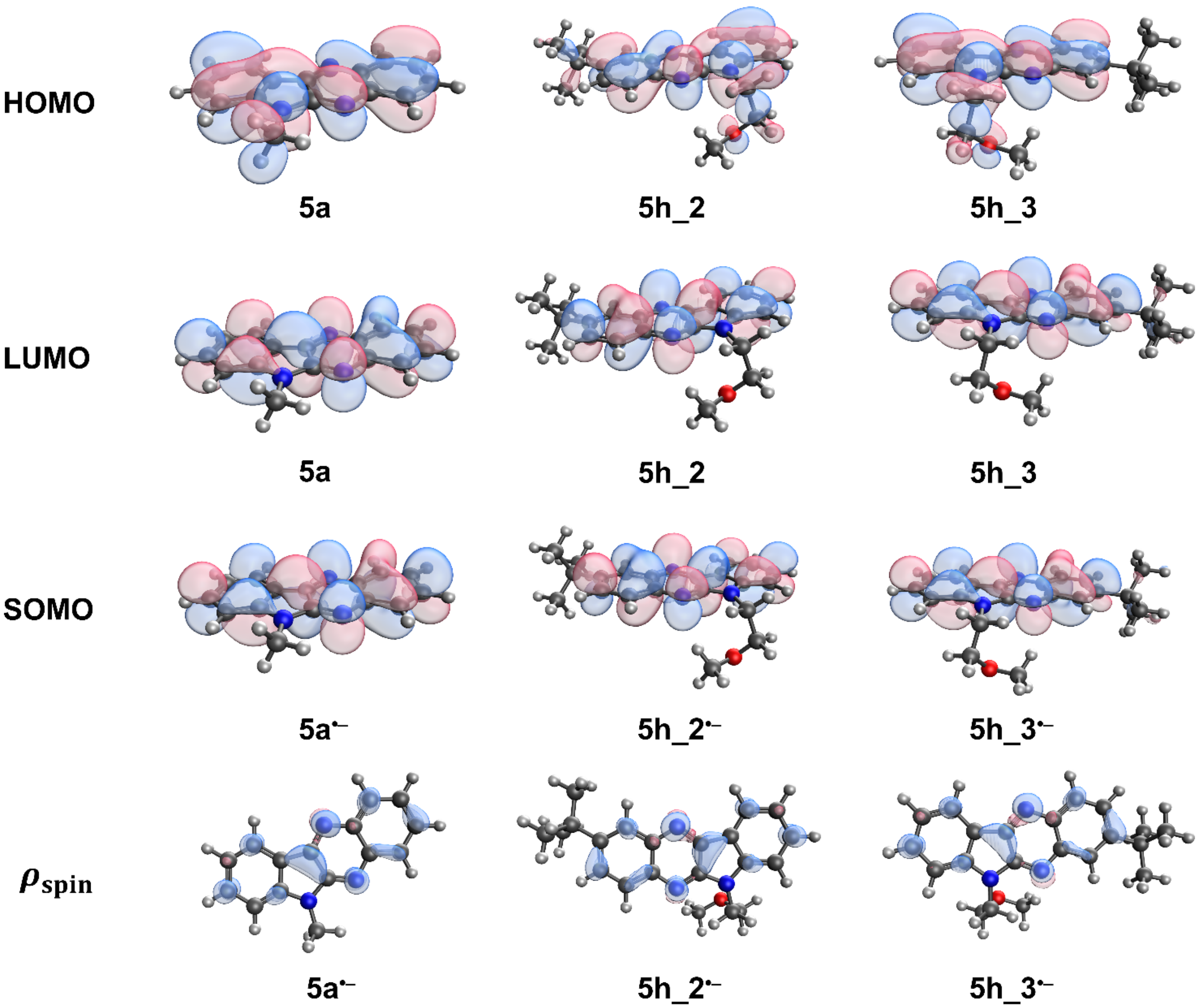

Figure SI 29. HOMO, LUMO, and SUMO for **5a**, **5h_2** and **5h_3**. ρ_{spin} is the spin density, the difference of spin-up electron density and spin-down electron density (positive: blue, negative: red). It shows that the excess electron is delocalized within the π-system.

IX. References

(1) Milshtein, J. D.; Kaur, A. P.; Casselman, M. D.; Kowalski, J. A.; Modekrutti, S.; Zhang, P. L.; Harsha Attanayake, N.; Elliott, C. F.; Parkin, S. R.; Risko, C.; Brushett, F. R.; Odom, S. A. High Current Density, Long Duration Cycling of Soluble Organic Active Species for Non-Aqueous Redox Flow Batteries. *Energy Environ. Sci.* **2016**, *9* (11), 3531–3543.

(2) Bard, A. J.; Faulkner, L. R. *Electrochemical Methods: Fundamentals and Applications*, 2nd ed.; Wiley: New York, 2001.

(3) Nicholson, R. S. Theory and Application of Cyclic Voltammetry for Measurement of Electrode Reaction Kinetics. *Anal. Chem.* **1965**, *37* (11), 1351–1355.

(4) Lavagnini, I.; Antiochia, R.; Magno, F. An Extended Method for the Practical Evaluation of the Standard Rate Constant from Cyclic Voltammetric Data. *Electroanalysis* **2004**, *16* (6), 505–506.

(5) Wang, H.; Sayed, S. Y.; Luber, E. J.; Olsen, B. C.; Shirurkar, S. M.; Venkatakrishnan, S.; Tefashe, U. M.; Farquhar, A. K.; Smotkin, E. S.; McCreery, R. L.; Buriak, J. M. Redox Flow Batteries: How to Determine Electrochemical Kinetic Parameters. *ACS Nano* **2020**, *14* (3), 2575−2584.

(6) Laoire, C. O.; Mukerjee, S.; Abraham, K. M.; Plichta, E. J.; Hendrickson, M. A. Elucidating the Mechanism of Oxygen Reduction for Lithium-Air Battery Applications. *J. Phys. Chem. C* **2009**, *113*, 20127−20134.

(7) Hu, B.; Liu, T. L. Two Electron Utilization of Methyl Viologen Anolyte in Nonaqueous Organic Redox Flow Battery. *J. Energy Chem.* **2018**, *27*, 1326−1332.

(8) Ahn, S.; Jang, J. H.; Kang, J.; Na, M.; Seo, J.; Singh, V.; Joo, J. M.; Byon, H. R. Systematic Designs of Dicationic Heteroarylpyridiniums as Negolytes for Nonaqueous Redox Flow Batteries. *ACS Energy Lett.* **2021**, *6*, 3390−3397.

(9) De La Garza, G. D.; Kaur, A. P.; Shkrob, I. A.; Robertson, L. A.; Odom, S. A.; McNeil, A. J. Soluble and Stable Symmetric Tetrazines as Anolytes in Redox Flow Batteries. *J. Mater. Chem. A* **2022**, *10*, 18745–18752.

(10) Wei, X.; Xu, W.; Huang, J.; Zhang, L.; Walter, E.; Lawrence, C.; Vijayakumar, M.; Henderson, W. A.; Liu, T.; Cosimbescu, L.; Li, B.; Sprenkle, V.; Wang, W. Radical Compatibility with Nonaqueous Electrolytes and Its Impact on an All-Organic Redox Flow Battery. *Angew. Chem., Int. Ed.* **2015**, *54*, 8684−8687.

(11) Duan, W.; Huang, J.; Kowalski, J. A.; Shkrob, I. A.; Vijayakumar, M.; Walter, E.; Pan, B.; Yang, Z.; Milshtein, J. D.; Li, B.; Liao, C.; Zhang, Z.; Wang, W.; Liu, J.; Moore, J. S.; Brushett, F. R.; Zhang, L.; Wei, X. Wine-Dark Sea" in an Organic Flow Battery: Storing Negative Charge in 2,1,3-Benzothiadiazole Radicals Leads to Improved Cyclability. *ACS Energy Lett.* **2017**, *2* (5), 1156−1161.

(12) Zhang, L.; Qian, Y.; Feng, R.; Ding, Y.; Zu, X.; Zhang, C.; Guo, X.; Wang, W.; Yu, G. Reversible Redox Chemistry in Azobenzene Based Organic Molecules for High-capacity and Long-life Nonaqueous Redox Flow Batteries. *Nat. Commun.* **2020**, *11*, 3843.

(13) Wang, X.; Chai, J.; Lashgari, A.; Jiang, J. J. Azobenzene-Based Low-Potential Anolyte for Nonaqueous Organic Redox Flow Batteries. *ChemElectroChem.* **2021**, *8*, 83−89.

(14) Wei, X.; Duan, W.; Huang, J.; Zhang, L.; Li, B.; Reed, D.; Xu, W.; Sprenkle, V.; Wang, W. A High-Current, Stable Nonaqueous Organic Redox Flow Battery. *ACS Energy Lett.* **2016**, *1*, 705−711.

(15) Yan, Y.; Zhang, L.; Walser-Kuntz, R.; Vogt, D. B.; Sigman, M. S.; Yu, G.; Sanford, M. S. Benzotriazoles as Low-Potential Anolytes for Non-Aqueous Redox Flow Batteries. *Chem. Mater.* **2022**, *34*, 10594−10605.

(16) Romadina, E. I.; Komarov, D. S.; Stevenson, K. J.; Troshin, P. A. New Phenazine Based Anolyte Material for High Voltage Organic Redox Flow Batteries. *Chem. Commun.* **2021**, *57*, 2986−2989.

(17) Steen, J. S.; Nuismer, J. L.; Eiva, V.; Wiglema, A. E. T.; Daub, N.; Hjelm, J.; Otten, E. Blatter Radicals as Bipolar Materials for Symmetrical Redox-Flow Batteries. *J. Am. Chem. Soc.* **2022**, *144*, 5051−5058.

(18) Tracy, J. S.; Horst, E. S.; Roytman, V. A.; Toste, F. D. Development of High-voltage Bipolar Redox-Active Organic Molecules Through the Electronic Coupling of Catholyte and Anolyte Structures. *Chem. Sci.* **2022**, *13*, 10806−10814.

(19) Liu, Y.; Dai, G.; Chen, Y.; Wang, R.; Li, H.; Shi, X.; Zhang, X.; Xu, Y.; Zhao, Y. Effective Design Strategy of Small Bipolar Molecules through Fused Conjugation toward 2.5 V Based Redox Flow Batteries. *ACS Energy Lett.* **2022**, *7,* 1274−1283.

(20) Epifanovsky, E.; Gilbert, A. T. B.; Feng, X.; Lee, J.; Mao, Y.; Mardirossian, N.; Pokhilko, P.; White, A. F.; Coons, M. P.; Dempwolff, A. L.; Gan, Z.; Hait, D.; Horn, P. R.; Jacobson, L. D.; Kaliman, I.; Kussmann, J.; Lange, A. W.; Lao, K. U.; Levine, D. S.; Liu, J.; McKenzie, S. C.; Morrison, A. F.; Nanda, K. D.; Plasser, F.; Rehn, D. R.; Vidal, M. L.; You, Z.-Q.; Zhu, Y.; Alam, B.; Albrecht, B. J.; Aldossary, A.; Alguire, E.; Andersen, J. H.; Athavale, V.; Barton, D.; Begam, K.; Behn, A.; Bellonzi, N.; Bernard, Y. A.; Berquist, E. J.; Burton, H. G. A.; Carreras, A.; Carter-Fenk, K.; Chakraborty, R.; Chien, A. D.; Closser, K. D.; Cofer-Shabica, V.; Dasgupta, S.; de Wergifosse, M.; Deng, J.; Diedenhofen, M.; Do, H.; Ehlert, S.; Fang, P.-T.; Fatehi, S.; Feng, Q.; Friedhoff, T.; Gayvert, J.; Ge, Q.; Gidofalvi, G.; Goldey, M.; Gomes, J.; González-Espinoza, C. E.; Gulania, S.; Gunina, A. O.; Hanson-Heine, M. W. D.; Harbach, P. H. P.; Hauser, A.; Herbst, M. F.; Hernández Vera, M.; Hodecker, M.; Holden, Z. C.; Houck, S.; Huang, X.; Hui, K.; Huynh, B. C.; Ivanov, M.; Jász, Á.; Ji, H.; Jiang, H.; Kaduk, B.; Kähler, S.; Khistyaev, K.; Kim, J.; Kis, G.; Klunzinger, P.; Koczor-Benda, Z.; Koh, J. H.; Kosenkov, D.; Koulias, L.; Kowalczyk, T.; Krauter, C. M.; Kue, K.; Kunitsa, A.; Kus, T.; Ladjánszki, I.; Landau, A.; Lawler, K. V.; Lefrancois, D.; Lehtola, S.; Li, R. R.; Li, Y.-P.; Liang, J.; Liebenthal, M.; Lin, H.-H.; Lin, Y.-S.; Liu, F.; Liu, K.-Y.; Loipersberger, M.; Luenser, A.; Manjanath, A.; Manohar, P.; Mansoor, E.; Manzer, S. F.; Mao, S.-P.; Marenich, A. V.; Markovich, T.; Mason, S.; Maurer, S. A.; McLaughlin, P. F.; Menger, M. F. S. J.; Mewes, J.-M.; Mewes, S. A.; Morgante, P.; Mullinax, J. W.; Oosterbaan, K. J.; Paran, G.; Paul, A. C.; Paul, S. K.; Pavošević, F.; Pei, Z.; Prager, S.; Proynov, E. I.; Rák, Á.; Ramos-Cordoba, E.; Rana, B.; Rask, A. E.; Rettig, A.; Richard, R. M.; Rob, F.; Rossomme, E.; Scheele, T.; Scheurer, M.; Schneider, M.; Sergueev, N.; Sharada, S. M.; Skomorowski, W.; Small, D. W.; Stein, C. J.; Su, Y.-C.; Sundstrom, E. J.; Tao, Z.; Thirman, J.; Tornai, G. J.; Tsuchimochi, T.; Tubman, N. M.; Veccham, S. P.; Vydrov, O.; Wenzel, J.; Witte, J.; Yamada, A.; Yao, K.; Yeganeh, S.; Yost, S. R.; Zech, A.; Zhang, I. Y.; Zhang, X.; Zhang, Y.; Zuev, D.; Aspuru-Guzik, A.; Bell, A. T.; Besley, N. A.; Bravaya, K. B.; Brooks, B. R.; Casanova, D.; Chai, J.-D.; Coriani, S.; Cramer, C. J.; Cserey, G.; DePrince, A. E.; DiStasio, R. A.; Dreuw, A.; Dunietz, B. D.; Furlani, T. R.; Goddard, W. A.; Hammes-Schiffer, S.; Head-Gordon, T.; Hehre, W. J.; Hsu, C.-P.; Jagau, T.-C.; Jung, Y.; Klamt, A.; Kong, J.; Lambrecht, D. S.; Liang, W.; Mayhall, N. J.; McCurdy, C. W.; Neaton, J. B.; Ochsenfeld, C.; Parkhill, J. A.; Peverati, R.; Rassolov, V. A.; Shao, Y.; Slipchenko, L. V.; Stauch, T.; Steele, R. P.; Subotnik, J. E.; Thom, A. J. W.; Tkatchenko, A.; Truhlar, D. G.; Van Voorhis, T.; Wesolowski, T. A.; Whaley, K. B.; Woodcock, H. L.; Zimmerman, P. M.; Faraji, S.; Gill, P. M. W.; Head-Gordon, M.; Herbert, J. M.; Krylov, A. I. Software for the Frontiers of Quantum Chemistry: An Overview of Developments in the Q-Chem 5 Package. *J. Chem. Phys.* **2021**, *155* (8), 084801.

(21) Bannwarth, C.; Ehlert, S.; Grimme, S. GFN2-XTB—An Accurate and Broadly Parametrized Self-Consistent Tight-Binding Quantum Chemical Method with Multipole Electrostatics and Density-Dependent Dispersion Contributions. *J. Chem. Theory Comput.* **2019**, *15* (3), 1652–1671.

(22) Bannwarth, C.; Caldeweyher, E.; Ehlert, S.; Hansen, A.; Pracht, P.; Seibert, J.; Spicher, S.; Grimme, S. Review GFNn-XTB. *WIREs Comput Mol Sci* **2021**, *11* (2), e1493.

(23) Pracht, P.; Bohle, F.; Grimme, S. Automated Exploration of the Low-Energy Chemical Space with Fast Quantum Chemical Methods. *Phys. Chem. Chem. Phys.* **2020**, *22* (14), 7169–7192.

(24) Ehlert, S.; Stahn, M.; Spicher, S.; Grimme, S. Robust and Efficient Implicit Solvation Model for Fast Semiempirical Methods. *J. Chem. Theory Comput.* **2021**, *17* (7), 4250–4261.

(25) Chai, J.-D.; Head-Gordon, M. Long-Range Corrected Hybrid Density Functionals with Damped Atom–Atom Dispersion Corrections. *Phys. Chem. Chem. Phys.* **2008**, *10* (44), 6615.

(26) Weigend, F.; Ahlrichs, R. Balanced Basis Sets of Split Valence, Triple Zeta Valence and Quadruple Zeta Valence Quality for H to Rn: Design and Assessment of Accuracy. *Phys. Chem. Chem. Phys.* **2005**, *7* (18), 3297.

(27) Rappoport, D.; Furche, F. Property-Optimized Gaussian Basis Sets for Molecular Response Calculations. *The Journal of Chemical Physics* **2010**, *133* (13), 134105.

(28) Cossi, M.; Rega, N.; Scalmani, G.; Barone, V. Energies, Structures, and Electronic Properties of Molecules in Solution with the C-PCM Solvation Model. *J. Comput. Chem.* **2003**, *24* (6), 669.
(29) Rappe, A. K.; Casewit, C. J.; Colwell, K. S.; Goddard, W. A.; Skiff, W. M. UFF, a Full Periodic Table Force Field for Molecular Mechanics and Molecular Dynamics Simulations. *J. Am. Chem. Soc.* **1992**, *114* (25), 10024.
(30) Spicher, S.; Grimme, S. Single-Point Hessian Calculations for Improved Vibrational Frequencies and Rigid-Rotor-Harmonic-Oscillator Thermodynamics. *J. Chem. Theory Comput.* **2021**, *17* (3), 1701–1714.
(31) Grimme, S. Supramolecular Binding Thermodynamics by Dispersion-Corrected Density Functional Theory. *Chem. Eur. J.* **2012**, *18* (32), 9955–9964.
(32) Grimme, S. Semiempirical GGA-Type Density Functional Constructed with a Long-Range Dispersion Correction. *J. Comput. Chem.* **2006**, *27* (15), 1787.
(33) Grimme, S.; Ehrlich, S.; Goerigk, L. Effect of the Damping Function in Dispersion Corrected Density Functional Theory. *J. Comput. Chem.* **2011**, *32* (7), 1456.
(34) Grimme, S.; Antony, J.; Ehrlich, S.; Krieg, H. A Consistent and Accurate *Ab Initio* Parametrization of Density Functional Dispersion Correction (DFT-D) for the 94 Elements H-Pu. *J. Chem. Phys.* **2010**, *132* (15), 154104.
(35) Perdew, J. P.; Burke, K.; Ernzerhof, M. Generalized Gradient Approximation Made Simple. *Phys. Rev. Lett.* **1996**, *77* (18), 3865–3868.
(36) Caldeweyher, E.; Bannwarth, C.; Grimme, S. Extension of the D3 Dispersion Coefficient Model. *J. Chem. Phys.* **2017**, *147* (3), 034112.
(37) Staroverov, V. N.; Scuseria, G. E.; Tao, J.; Perdew, J. P. Comparative Assessment of a New Nonempirical Density Functional: Molecules and Hydrogen-Bonded Complexes. *The Journal of Chemical Physics* **2003**, *119* (23), 12129–12137.
(38) Becke, A. D. Density-functional Thermochemistry. III. The Role of Exact Exchange. *The Journal of Chemical Physics* **1993**, *98* (7), 5648–5652.
(39) Stephens, P. J.; Devlin, F. J.; Chabalowski, C. F.; Frisch, M. J. Ab Initio Calculation of Vibrational Absorption and Circular Dichroism Spectra Using Density Functional Force Fields. *J. Phys. Chem.* **1994**, *98* (45), 11623–11627.
(40) Adamo, C.; Barone, V. Toward Reliable Density Functional Methods without Adjustable Parameters: The PBE0 Model. *The Journal of Chemical Physics* **1999**, *110* (13), 6158–6170.
(41) Zhao, Y.; Truhlar, D. G. Design of Density Functionals That Are Broadly Accurate for Thermochemistry, Thermochemical Kinetics, and Nonbonded Interactions. *J. Phys. Chem. A* **2005**, *109* (25), 5656–5667.
(42) Zhao, Y.; Truhlar, D. G. Hybrid Meta Density Functional Theory Methods for Thermochemistry, Thermochemical Kinetics, and Noncovalent Interactions: The MPW1B95 and MPWB1K Models and Comparative Assessments for Hydrogen Bonding and van Der Waals Interactions. *J. Phys. Chem. A* **2004**, *108* (33), 6908–6918.
(43) Mardirossian, N.; Head-Gordon, M. ω B97M-V: A Combinatorially Optimized, Range-Separated Hybrid, Meta-GGA Density Functional with VV10 Nonlocal Correlation. *The Journal of Chemical Physics* **2016**, *144* (21), 214110.
(44) Mardirossian, N.; Head-Gordon, M. ΩB97X-V: A 10-Parameter, Range-Separated Hybrid, Generalized Gradient Approximation Density Functional with Nonlocal Correlation, Designed by a Survival-of-the-Fittest Strategy. *Phys. Chem. Chem. Phys.* **2014**, *16* (21), 9904–9924.
(45) Lin, Y.-S.; Li, G.-D.; Mao, S.-P.; Chai, J.-D. Long-Range Corrected Hybrid Density Functionals with Improved Dispersion Corrections. *J. Chem. Theory Comput.* **2013**, *9* (1), 263–272.
(46) Dasgupta, S.; Herbert, J. M. Standard Grids for High-precision Integration of Modern Density Functionals: SG-2 and SG-3. *J. Comput. Chem.* **2017**, *38* (12), 869–882.
(47) Gill, P. M. W.; Johnson, B. G.; Pople, J. A. A Standard Grid for Density Functional Calculations. *Chem. Phys. Lett.* **1993**, *209* (5–6), 506.

(48) Ho, J.; Coote, M.; Cramer, C.; Truhlar, D. Theoretical Calculation of Reduction Potentials. In *Theoretical Calculation of Reduction Potentials*; Hammerich, O., Speiser, B., Eds.; CRC Press, Taylor and Francis Group, 2015; pp 229–259.
(49) Weinhold, F.; Landis, C. R.; Glendening, E. D. What Is NBO Analysis and How Is It Useful? *International Reviews in Physical Chemistry* **2016**, *35* (3), 399–440.
(50) Glendening, E. D.; Landis, C. R.; Weinhold, F. Natural Bond Orbital Methods. *WIREs Comput Mol Sci* **2012**, *2* (1), 1–42.
(51) Szabo, A.; Ostlund, N. S. *Modern Quantum Chemistry: Introduction to Advanced Electronic Structure Theory*; Dover Publications: Mineola, N.Y, 1996.
(52) Breneman, C. M.; Wiberg, K. B. Determining Atom-Centered Monopoles from Molecular Electrostatic Potentials. The Need for High Sampling Density in Formamide Conformational Analysis. *J. Comput. Chem.* **1990**, *11* (3), 361–373.
(53) Herbert, J. M.; Jacobson, L. D.; Un Lao, K.; Rohrdanz, M. A. Rapid Computation of Intermolecular Interactions in Molecular and Ionic Clusters: Self-Consistent Polarization plus Symmetry-Adapted Perturbation Theory. *Phys. Chem. Chem. Phys.* **2012**, *14* (21), 7679.
